# Supplementary material for: Comparative Efficacy and Safety of Tirbanibulin for Actinic Keratosis of the Face and Scalp in Europe: A Systematic Review and Network Meta-Analysis of Randomized Controlled Trials
Source: J Clin Med. 2022 Mar 16;11(6):1654. doi: 10.3390/jcm11061654 (PMC8952421; doi:10.3390/jcm11061654)
Supplement: Supplementary file 1 [file jcm-11-01654-s001.zip › 1.pdf]

# Comparative efficacy and safety of tirbanibulin for actinic keratosis of the face and scalp in Europe: A systematic review and network meta-analysis of randomised controlled trials

**Supplementary file**

# 1 Methods

## 1.1 Systematic review methods

### Supplementary Figure S1: Search strategies

**Source: MEDLINE(R) ALL**
Interface / URL: OvidSP
Database coverage dates: 1946 to June 22, 2020
Search date: 24/06/20
Retrieved records: 1210
Search strategy:

1 Keratosis, Actinic / (2048)
2 (actinic$ adj6 (keratos$ or keratoma$ or keratotic$ or keratopath$)).ti,ab,kf. (3649)
3 (actinic$ adj6 (cheilit$ or cheilos$)).ti,ab,kf. (339)
4 ((solar$ or senil$) adj6 (keratos$ or keratoma$ or keratotic$ or keratopath$)).ti,ab,kf. (647)
5 ((solar$ or senil$) adj6 (cheilit$ or cheilos$)).ti,ab,kf. (16)
6 (hyperkeratos$ or hyperkeratoma$ or hyperkeratotic$ or hyperkeratopath$).ti,ab,kf. (6790)
7 (keratinocytic intraepidermal neoplasia$ or keratinocytic intra-epidermal neoplasia$).ti,ab,kf. (13)
8 ((sailor$ or farmer$) adj (lip or lips)).ti,ab,kf. (3)
9 (ak or aks).ti,ab,kf. (7668)
10 (hak or haks).ti,ab,kf. (241)
11 (kin or kins).ti,ab,kf. (6501)
12 or / 1-11 (24881)
13 tirbanibulin$.ti,ab,kf,rn,nm. (2)
14 (alm-14789$2 or alm14789$2 or kx01$2 or kx-01$2 or kx2391$2 or kx2-391$2 or kx-2391$2 or kx-2-391$2).ti,ab,kf,rn,nm. (28)
15 (1080645-95-9 or 4v9848rs5g or 84ac3796wj or 897016-82-9).ti,ab,kf,rn,nm. (0)
16 Diclofenac / (7820)
17 diclofenac$.ti,ab,kf,rn,nm. (13203)
18 (abitren$2 or aceclofenac$2 or benfofen$2 or dealgic$2 or deflamat$2 or delphinac$2 or dichlofenal$2 or dicloflex$2 or diclomax$2 or diclonate$ or diclophenac$2 or diclophlogont$2 or dicloreum$2 or dicrofenac$2 or duravolten$2 or dyloject$2 or ecofenac$2 or effekton$2 or feloran$2 or gp45840$2 or gp-45840$2 or las41007$2 or las-41007$2 or lexobene$2 or neriodin$2 or novapirina$2 or orthofen$2 or orthophen$2 or ortofen$2 or pennsaid$2 or primofenac$2 or prophenatin$2 or rewodina$2 or rhumalgan$2 or solacutan$2 or solaraze$2 or sr-38$2 or sr38$2 or voldal$2 or voltaren$2 or voltarol$2 or xenid$2).ti,ab,kf,rn,nm. (1080)
19 (cataflam$2 or d11ax18 or m01ab05 or m02aa15 or s01bc03).ti,ab,kf,rn,nm. (18)
20 (15307-79-6 or 239-346-4 or qtg126297q or 144o8ql0l1 or 15307-86-5 or 239-348-5 or 15307-81-0 or l4d5ua6cb4).ti,ab,kf,rn,nm. (7831)
21 Fluorouracil / (41350)
22 (fluorouracil$ or fluoro-uracil$).ti,ab,kf,rn,nm. (54203)
23 (5fluoropyrimidin$2 or 5-fluoropyrimidin$2 or 5fluoropyrimidine$2 or 5-fluoropyrimidine$2 or 5fluorouracil$2 or 5fluoro-uracil$2 or 5fluoruracil$2 or 5fu$2 or 5-fu$2 or 5hu hexal$2 or 5-hu hexal$2 or accusite$2 or actikerall$2 or actikerell$2 or actino-hermal$2 or adrucil$2 or agicil$2 or arac$2 or carac$2 or cinkef-u$2 or effluderm$2 or efudex$2 or efudix$2 or efurix$2 or emtricitabine impurity f$2 or eurofluor$2 or f6627$2 or f-6627$2 or fivoflu$2 or flucytosine impurity a$2 or fluoroblastin$2 or fluoroplex$2 or fluorouricil$2 or fluoruracil$2 or fluouracil$2 or fluoxan$2 or flurablastin$2 or

fluracedyl$2 or fluracil$2 or fluracilium$2 or fluril$2 or fluroblastin$2 or fluroblastine$2 or fluro-uracil$2 or flurodex$2 or haemato-fu$2 or ifacil$2 or l01bc02$2 or neofluor$2 or nsc-18913$2 or nsc-19893$2 or nsc18913$2 or nsc19893$2 or oncofu$2 or onkofluor$2 or phthoruracil$2 or queroplex$2 or ribofluor$2 or ro-2-9757$2 or ro2-9757$2 or ro-29757$2 or ro29757$2 or tolak$2 or uflahex$2 or utoral$2 or verrumal$2).ti,ab,kf,rn,nm. (24747)
24 (10318-20-4 or 200-085-6 or 51-21-8 or 57050-04-1 or 57172-36-8 or 68021-61-4 or u3p01618rt or 15fs54053u or 56177-80-1 or 260-029-1 or q0e5j9oq9o or 766-63-2 or pm476l7o8g or 14787-18-9).ti,ab,kf,rn,nm. (41353)
25 Imiquimod / (2485)
26 imiquimod$.ti,ab,kf,rn,nm. (3745)
27 (aldara$2 or d06bb10 or imunocare$2 or r-837$2 or r837$2 or s-26308$2 or s26308$2 or zartra$2 or zyclara$2).ti,ab,kf,rn,nm. (341)
28 (99011-02-6 or p1qw714r7m).ti,ab,kf,rn,nm. (2485)
29 ingenol$ mebutate$.ti,ab,kf,rn,nm. (314)
30 (agn-204332$2 or agn204332$2 or d06bx02 or ingenol-3-angelate$ or ingenol-3-mebutate$ or ingenol$ angelate$ or pep-005$2 or pep005$2 or picato$2).ti,ab,kf,rn,nm. (81)
31 (75567-37-2 or 7686s50jah).ti,ab,kf,rn,nm. (0)
32 or / 13-31 (77448)
33 Cryotherapy / (5083)
34 Cryosurgery / (12887)
35 (cryotherap$ or cryo-therap$ or cryotreat$ or cryo-treat$).ti,ab,kf. (7587)
36 (cryoablat$ or cryo-ablat$ or cryoapplication$ or cryo-application$ or cryoblat$ or cryo-blat$ or cryocoag$ or cryo-coag$ or cryodestr$ or cryo-destr$ or cryoenerg$ or cryo-energ$ or cryogen$ or cryo-gen$ or cryoisolat$ or cryo-isolat$ or cryosurg$ or cryo-surg$ or cryotherm$ or cryo-therm$).ti,ab,kf. (16768)
37 (freez$ or froz$).ti,ab,kf. (136808)
38 (liquid adj (nitrogen or n2)).ti,ab,kf. (8553)
39 ln2.ti,ab,kf. (662)
40 (cold adj (treatment$ or therap$)).ti,ab,kf. (1563)
41 or / 33-40 (169803)
42 phototherapy / or intense pulsed light therapy / or low-level light therapy / or exp photochemotherapy / (33672)
43 Photosensitizing Agents / (15934)
44 (photodynamic$ or photo-dynamic$).ti,ab,kf. (25233)
45 (photochemotherap$ or photo-chemotherap$ or photoradiat$ or photo-radiat$ or photosensiti$ or photo-sensiti$ or phototherap$ or photo-therap$).ti,ab,kf. (39838)
46 chemophototherap$.ti,ab,kf. (31)
47 (light adj (treatment$ or therap$)).ti,ab,kf. (4577)
48 pdt.ti,ab,kf. (13630)
49 or / 42-48 (74403)
50 12 and (32 or 41 or 49) (2289)
51 randomized controlled trial.pt. (508066)
52 controlled clinical trial.pt. (93724)
53 randomized.ab. (483747)
54 placebo.ab. (208801)
55 drug therapy.fs. (2213019)
56 randomly.ab. (335722)
57 trial.ab. (509998)
58 groups.ab. (2060491)
59 51 or 52 or 53 or 54 or 55 or 56 or 57 or 58 (4731122)
60 50 and 59 (1469)

61 exp Animals / not Humans / (4709505)
62 (news or case reports).pt. or case report.ti. (2350019)
63 60 not (61 or 62) (1213)
64 remove duplicates from 63 (1210)

Key to Ovid symbols and commands

| | |
|---|---|
| $ | Unlimited right-hand truncation symbol |
| $N | Limited right-hand truncation - restricts the number of characters following the word to N |
| ti,ab,kf,rn,nm | Searches are restricted to the Title (ti), Abstract (ab), Keyword Heading Word (kf), Registry Number / Name of Substance (rn) and Name of Substance Word (nm) fields |
| adj | Retrieves records that contain terms next to each other (in the shown order) |
| adjN | Retrieves records that contain terms (in any order) within a specified number (N) of words of each other |
| / | Searches are restricted to the Subject Heading field |
| exp | The subject heading is exploded |
| pt. | Search is restricted to the publication type field |
| or / 1-11 | Combines sets 1 to 11 using OR |
| .fs. | Term is searched as a floating subheading |

**Source: Embase**
Interface / URL: OvidSP
Database coverage dates: 1974 to 2020 June 24
Search date: 25/06/20
Retrieved records: 881
Search strategy:

1 Keratosis, Actinic/ (4505)
2 (actinic$ adj6 (keratos$ or keratoma$ or keratotic$ or keratopath$)).ti,ab,kw,dq. (5358)
3 (actinic$ adj6 (cheilit$ or cheilos$)).ti,ab,kw,dq. (444)
4 ((solar$ or senil$) adj6 (keratos$ or keratoma$ or keratotic$ or keratopath$)).ti,ab,kw,dq. (783)
5 ((solar$ or senil$) adj6 (cheilit$ or cheilos$)).ti,ab,kw,dq. (21)
6 (hyperkeratos$ or hyperkeratoma$ or hyperkeratotic$ or hyperkeratopath$).ti,ab,kw,dq. (9732)
7 (keratinocytic intraepidermal neoplasia$ or keratinocytic intra-epidermal neoplasia$).ti,ab,kw,dq. (14)
8 ((sailor$ or farmer$) adj (lip or lips)).ti,ab,kw,dq. (2)
9 (ak or aks).ti,ab,kw,dq. (7341)
10 (hak or haks).ti,ab,kw,dq. (249)
11 (kin or kins).ti,ab,kw,dq. (7821)
12 or/1-11 (30960)
13 tirbanibulin/ (7)
14 tirbanibulin$.ti,ab,kw,rn,tn,dq,dy. (7)
15 (alm-14789$2 or alm14789$2 or kx01$2 or kx-01$2 or kx2391$2 or kx2-391$2 or kx-2391$2 or kx-2-391$2).ti,ab,kw,rn,tn,dq,dy. (95)
16 (1080645-95-9 or 4v9848rs5g or 84ac3796wj or 897016-82-9).ti,ab,kw,rn,tn,dq,dy. (7)
17 diclofenac/ (39993)
18 diclofenac$.ti,ab,kw,rn,tn,dq,dy. (41834)
19 (abitren$2 or aceclofenac$2 or benfofen$2 or dealgic$2 or deflamat$2 or delphinac$2 or dichlofenal$2 or dicloflex$2 or diclomax$2 or diclonate$ or diclophenac$2 or diclophlogont$2 or

dicloreum$2 or dicrofenac$2 or duravolten$2 or dyloject$2 or ecofenac$2 or effekton$2 or feloran$2 or gp45840$2 or gp-45840$2 or las41007$2 or las-41007$2 or lexobene$2 or neriodin$2 or novapirina$2 or orthofen$2 or orthophen$2 or ortofen$2 or pennsaid$2 or primofenac$2 or prophenatin$2 or rewodina$2 or rhumalgan$2 or solacutan$2 or solaraze$2 or sr-38$2 or sr38$2 or voldal$2 or voltaren$2 or voltarol$2 or xenid$2).ti,ab,kw,rn,tn,dq,dy. (5551)
20 (cataflam$2 or d11ax18 or m01ab05 or m02aa15 or s01bc03).ti,ab,kw,rn,tn,dq,dy. (231)
21 (15307-79-6 or 239-346-4 or qtg126297q or 144o8ql0l1 or 15307-86-5 or 239-348-5 or 15307-81-0 or l4d5ua6cb4).ti,ab,kw,rn,tn,dq,dy. (38312)
22 fluorouracil/ (137520)
23 fluorouracil plus salicylic acid/ (17)
24 (fluorouracil$ or fluoro-uracil$).ti,ab,kw,rn,tn,dq,dy. (142589)
25 (5fluoropyrimidin$2 or 5-fluoropyrimidin$2 or 5fluoropyrimidine$2 or 5-fluoropyrimidine$2 or 5fluorouracil$2 or 5fluoro-uracil$2 or 5fluoruracil$2 or 5fu$2 or 5-fu$2 or 5hu hexal$2 or 5-hu hexal$2 or accusite$2 or actikerall$2 or actikerell$2 or actino-hermal$2 or adrucil$2 or agicil$2 or arac$2 or carac$2 or cinkef-u$2 or effluderm$2 or efudex$2 or efudix$2 or efurix$2 or emtricitabine impurity f$2 or eurofluor$2 or f6627$2 or f-6627$2 or fivoflu$2 or flucytosine impurity a$2 or fluoroblastin$2 or fluoroplex$2 or fluorouricil$2 or fluoruracil$2 or fluouracil$2 or fluoxan$2 or flurablastin$2 or fluracedyl$2 or fluracil$2 or fluracilium$2 or fluril$2 or fluroblastin$2 or fluroblastine$2 or fluro-uracil$2 or flurodex$2 or haemato-fu$2 or ifacil$2 or l01bc02$2 or neofluor$2 or nsc-18913$2 or nsc-19893$2 or nsc18913$2 or nsc19893$2 or oncofu$2 or onkofluor$2 or phthoruracil$2 or queroplex$2 or ribofluor$2 or ro-2-9757$2 or ro2-9757$2 or ro-29757$2 or ro29757$2 or tolak$2 or uflahex$2 or utoral$2 or verrumal$2).ti,ab,kw,rn,tn,dq,dy. (39041)
26 (10318-20-4 or 200-085-6 or 51-21-8 or 57050-04-1 or 57172-36-8 or 68021-61-4 or u3p01618rt or 15fs54053u or 56177-80-1 or 260-029-1 or q0e5j9oq9o or 766-63-2 or pm476l7o8g or 14787-18-9).ti,ab,kw,rn,tn,dq,dy. (128723)
27 imiquimod/ (8934)
28 imiquimod$.ti,ab,kw,rn,tn,dq,dy. (9445)
29 (aldara$2 or d06bb10 or imunocare$2 or r-837$2 or r837$2 or s-26308$2 or s26308$2 or zartra$2 or zyclara$2).ti,ab,kw,rn,tn,dq,dy. (1514)
30 (99011-02-6 or p1qw714r7m).ti,ab,kw,rn,tn,dq,dy. (7849)
31 ingenol mebutate/ (680)
32 ingenol$ mebutate$.ti,ab,kw,rn,tn,dq,dy. (734)
33 (agn-204332$2 or agn204332$2 or d06bx02 or ingenol-3-angelate$ or ingenol-3-mebutate$ or ingenol$ angelate$ or pep-005$2 or pep005$2 or picato$2).ti,ab,kw,rn,tn,dq,dy. (254)
34 (75567-37-2 or 7686s50jah).ti,ab,kw,rn,tn,dq,dy. (553)
35 or/13-34 (200585)
36 exp cryotherapy/ (32896)
37 cryotherapy device/ (51)
38 (cryotherap$ or cryo-therap$ or cryotreat$ or cryo-treat$).ti,ab,kw,dv,dq,my. (10941)
39 (cryoablat$ or cryo-ablat$ or cryoapplication$ or cryo-application$ or cryoblat$ or cryo-blat$ or cryocoag$ or cryo-coag$ or cryodestr$ or cryo-destr$ or cryoenerg$ or cryo-energ$ or cryogen$ or cryo-gen$ or cryoisolat$ or cryo-isolat$ or cryosurg$ or cryo-surg$ or cryotherm$ or cryo-therm$).ti,ab,kw,dv,dq,my. (19633)
40 (freez$ or froz$).ti,ab,kw,dv,dq,my. (176928)
41 liquid nitrogen/ (7227)
42 (liquid adj (nitrogen or n2)).ti,ab,kw,dv,dq,my. (11605)
43 ln2.ti,ab,kw,dv,dq,my. (937)
44 (cold adj (treatment$ or therap$)).ti,ab,kw,dv,dq,my. (1403)
45 or/36-44 (223483)
46 exp photochemotherapy/ or phototherapy/ or intense pulsed light therapy/ or low level laser therapy/ (85428)

47 photosensitizing agent/ (14828)
48 (photodynamic$ or photo-dynamic$).ti,ab,kw,dv,dq,my. (30228)
49 (photochemotherap$ or photo-chemotherap$ or photoradiat$ or photo-radiat$ or photosensiti$ or photo-sensiti$ or phototherap$ or photo-therap$).ti,ab,kw,dv,dq,my. (49018)
50 chemophototherap$.ti,ab,kw,dv,dq,my. (34)
51 (light adj (treatment$ or therap$)).ti,ab,kw,dv,dq,my. (5591)
52 pdt.ti,ab,kw,dv,dq,my. (17017)
53 or/46-52 (120852)
54 12 and (35 or 45 or 53) (4561)
55 Randomized controlled trial/ (607969)
56 Controlled clinical study/ (463992)
57 Random$.ti,ab. (1544012)
58 randomization/ (87071)
59 intermethod comparison/ (260580)
60 placebo.ti,ab. (307111)
61 (compare or compared or comparison).ti. (510393)
62 ((evaluated or evaluate or evaluating or assessed or assess) and (compare or compared or comparing or comparison)).ab. (2121426)
63 (open adj label).ti,ab. (79627)
64 ((double or single or doubly or singly) adj (blind or blinded or blindly)).ti,ab. (232697)
65 double blind procedure/ (173401)
66 parallel group$1.ti,ab. (25605)
67 (crossover or cross over).ti,ab. (105255)
68 ((assign$ or match or matched or allocation) adj5 (alternate or group$1 or intervention$1 or patient$1 or subject$1 or participant$1)).ti,ab. (330911)
69 (assigned or allocated).ti,ab. (389265)
70 (controlled adj7 (study or design or trial)).ti,ab. (349827)
71 (volunteer or volunteers).ti,ab. (246980)
72 human experiment/ (500451)
73 trial.ti. (301581)
74 or/55-73 (5029155)
75 (random$ adj sampl$ adj7 ("cross section$" or questionnaire$1 or survey$ or database$1)).ti,ab. not (comparative study/ or controlled study/ or randomi?ed controlled.ti,ab. or randomly assigned.ti,ab.) (8079)
76 Cross-sectional study/ not (randomized controlled trial/ or controlled clinical study/ or controlled study/ or randomi?ed controlled.ti,ab. or control group$1.ti,ab.) (237333)
77 (((case adj control$) and random$) not randomi?ed controlled).ti,ab. (17276)
78 (Systematic review not (trial or study)).ti. (144841)
79 (nonrandom$ not random$).ti,ab. (16145)
80 "Random field$".ti,ab. (2310)
81 (random cluster adj3 sampl$).ti,ab. (1278)
82 (review.ab. and review.pt.) not trial.ti. (803210)
83 "we searched".ab. and (review.ti. or review.pt.) (32091)
84 "update review".ab. (104)
85 (databases adj4 searched).ab. (35906)
86 (rat or rats or mouse or mice or swine or porcine or murine or sheep or lambs or pigs or piglets or rabbit or rabbits or cat or cats or dog or dogs or cattle or bovine or monkey or monkeys or trout or marmoset$1).ti. and animal experiment/ (1064694)
87 Animal experiment/ not (human experiment/ or human/) (2246558)
88 or/75-87 (3474234)
89 74 not 88 (4477050)

90 54 and 89 (913)
91 (animal/ or animal experiment/ or animal model/ or animal tissue/ or nonhuman/) not exp human/ (6019117)
92 case report.ti. (292163)
93 90 not (91 or 92) (900)
94 remove duplicates from 93 (881)

**Source: Cochrane Central Register of Controlled Trials**
Interface / URL: Cochrane Library / Wiley
Database coverage dates: Information not found. Issue searched: Issue 6 of 12, June 2020
Search date: 25/06/20
Retrieved records: 822
Search strategy:

#1 [mh ^"Keratosis, Actinic"] 389
#2 (actinic* near/6 (keratos* or keratoma* or keratotic* or keratopath*)) 1006
#3 (actinic* near/6 (cheilit* or cheilos*)) 20
#4 ((solar* or senil*) near/6 (keratos* or keratoma* or keratotic* or keratopath*)) 55
#5 ((solar* or senil*) near/6 (cheilit* or cheilos*)) 2
#6 (hyperkeratos* or hyperkeratoma* or hyperkeratotic* or hyperkeratopath*) 293
#7 (keratinocytic next intraepidermal next neoplasia* or keratinocytic next intra next epidermal next neoplasia*) 1
#8 ((sailor* or farmer*) next (lip or lips)) 1
#9 (ak or aks) not (ak or aks):au 1267
#10 (hak or haks) 149
#11 (kin or kins) 550
#12 #1 or #2 or #3 or #4 or #5 or #6 or #7 or #8 or #9 or #10 or #11 2664
#13 tirbanibulin* 0
#14 ("alm-14789" or "alm-14789r" or "alm-14789tm" or alm14789* or kx01* or "kx-01" or "kx-01r" or "kx-01tm" or kx2391* or "kx2-391" or "kx2-391r" or "kx2-391tm" or "kx-2391" or "kx-2391r" or "kx-2391tm" or "kx-2-391" or "kx-2-391r" or "kx-2-391tm") 1
#15 ("1080645-95-9" or 4v9848rs5g or 84ac3796wj or "897016-82-9") 0
#16 [mh ^Diclofenac] 1916
#17 diclofenac* 5300
#18 (abitren* or aceclofenac* or benfofen* or dealgic* or deflamat* or delphinac* or dichlofenal* or dicloflex* or diclomax* or diclonate* or diclophenac* or diclophlogont* or dicloreum* or dicrofenac* or duravolten* or dyloject* or ecofenac* or effekton* or feloran* or gp45840* or "gp-45840" or "gp-45840r" or "gp-45840tm" or las41007* or "las-41007" or "las-41007r" or "las-41007tm" or lexobene* or neriodin* or novapirina* or orthofen* or orthophen* or ortofen* or pennsaid* or primofenac* or prophenatin* or rewodina* or rhumalgan* or solacutan* or solaraze* or "sr-38" or "sr-38r" or "sr-38tm" or sr38* or voldal* or voltaren* or voltarol* or xenid*) 686
#19 (cataflam* or d11ax18 or m01ab05 or m02aa15 or s01bc03) 25
#20 ("15307-79-6" or "239-346-4" or qtg126297q or 144o8ql0l1 or "15307-86-5" or "239-348-5" or "15307-81-0" or l4d5ua6cb4) 0
#21 [mh ^Fluorouracil] 4881
#22 (fluorouracil* or fluoro next uracil*) 10464
#23 (5fluoropyrimidin* or 5 next fluoropyrimidin* or 5fluoropyrimidine* or 5 next fluoropyrimidine* or 5fluorouracil* or 5fluoro next uracil* or 5fluoruracil* or 5fu* or 5 next fu* or 5hu next hexal* or 5 next hu next hexal* or accusite* or actikerall* or actikerell* or actino next hermal* or adrucil* or agicil* or arac* or carac* or cinkef or effluderm* or efudex* or efudix* or efurix* or emtricitabine next impurity or eurofluor* or f6627* or "f-6627" or "f-6627r" or "f-6627tm" or fivoflu* or flucytosine next impurity or

fluoroblastin* or fluoroplex* or fluorouricil* or fluoruracil* or fluouracil* or fluoxan* or flurablastin* or fluracedyl* or fluracil* or fluracilium* or fluril* or fluroblastin* or fluroblastine* or fluro next uracil* or flurodex* or "haemato fu" or "haemato fur" or "haemato futm" or ifacil* or l01bc02* or neofluor* or "nsc-18913" or "nsc-18913r" or "nsc-18913tm" or "nsc-19893" or "nsc-19893r" or "nsc-19893tm" or nsc18913* or nsc19893* or oncofu* or onkofluor* or phthoruracil* or queroplex* or ribofluor* or "ro-2-9757" or "ro-2-9757r" or "ro-2-9757tm" or "ro2-9757" or "ro2-9757r" or "ro2-9757tm" or "ro-29757" or "ro-29757r" or "ro-29757tm" or ro29757* or tolak* or uflahex* or utoral* or verrumal*) 14274
#24 ("10318-20-4" or "200-085-6" or "51-21-8" or "57050-04-1" or "57172-36-8" or "68021-61-4" or u3p01618rt or 15fs54053u or "56177-80-1" or "260-029-1" or q0e5j9oq9o or "766-63-2" or pm476l7o8g or "14787-18-9") 0
#25 [mh ^Imiquimod] 244
#26 imiquimod* 550
#27 (aldara* or d06bb10 or imunocare* or "r-837" or "r-837r" or "r-837tm" or r837* or "s-26308" or "s-26308r" or "s-26308tm" or s26308* or zartra* or zyclara*) 97
#28 ("99011-02-6" or p1qw714r7m) 0
#29 ingenol* next mebutate* 124
#30 ("agn-204332" or "agn-204332r" or "agn-204332tm" or agn204332* or d06bx02 or ingenol next 3 next angelate* or ingenol next 3 next mebutate* or ingenol* next angelate* or "pep-005" or "pep-005r" or "pep-005tm" or pep005* or picato*) 35
#31 ("75567-37-2" or 7686s50jah) 0
#32 #13 or #14 or #15 or #16 or #17 or #18 or #19 or #20 or #21 or #22 or #23 or #24 or #25 or #26 or #27 or #28 or #29 or #30 or #31 27245
#33 [mh ^Cryotherapy] 661
#34 [mh "Cryosurgery"] 337
#35 (cryotherap* or cryo next therap* or cryotreat* or cryo next treat*) 2210
#36 (cryoablat* or cryo next ablat* or cryoapplication* or cryo next application* or cryoblat* or cryo next blat* or cryocoag* or cryo next coag* or cryodestr* or cryo next destr* or cryoenerg* or cryo next energ* or cryogen* or cryo next gen* or cryoisolat* or cryo next isolat* or cryosurg* or cryo next surg* or cryotherm* or cryo next therm*) 970
#37 (freez* or froz*) 6662
#38 (liquid next (nitrogen or n2)) 351
#39 ln2 22
#40 (cold next (treatment* or therap*)) 255
#41 #33 or #34 or #35 or #36 or #37 or #38 or #39 or #40 9604
#42 [mh ^phototherapy] or [mh ^"intense pulsed light therapy"] or [mh ^"low level light therapy"] or [mh photochemotherapy] 2552
#43 [mh ^"Photosensitizing Agents"]677
#44 (photodynamic* or photo next dynamic*) 2330
#45 (photochemotherap* or photo next chemotherap* or photoradiat* or photo next radiat* or photosensiti* or photo next sensiti* or phototherap* or photo next therap*) 5171
#46 chemophototherap* 6
#47 (light next (treatment* or therap*)) 2242
#48 pdt 1734
#49 #42 or #43 or #44 or #45 or #46 or #47 or #48 8254
#50 #12 and (#32 or #41 or #49) 1014
#51 #50 in Trials 822

**Source: Cochrane Database of Systematic Reviews**
Interface / URL: Cochrane Library / Wiley
Database coverage dates: Information not found. Issue 6 of 12, June 2020
Search date: 25/06/20

Retrieved records: 11
Search strategy:

#1 [mh ^"Keratosis, Actinic"] 389
#2 (actinic* near/6 (keratos* or keratoma* or keratotic* or keratopath*)):ti,ab,kw 980
#3 (actinic* near/6 (cheilit* or cheilos*)):ti,ab,kw 18
#4 ((solar* or senil*) near/6 (keratos* or keratoma* or keratotic* or keratopath*)):ti,ab,kw 45
#5 ((solar* or senil*) near/6 (cheilit* or cheilos*)):ti,ab,kw 0
#6 (hyperkeratos* or hyperkeratoma* or hyperkeratotic* or hyperkeratopath*):ti,ab,kw 260
#7 (keratinocytic next intraepidermal next neoplasia* or keratinocytic next intra next epidermal next neoplasia*):ti,ab,kw 0
#8 ((sailor* or farmer*) next (lip or lips)):ti,ab,kw 0
#9 (ak or aks):ti,ab,kw 803
#10 (hak or haks):ti,ab,kw 6
#11 (kin or kins):ti,ab,kw 397
#12 #1 or #2 or #3 or #4 or #5 or #6 or #7 or #8 or #9 or #10 or #11 1878
#13 tirbanibulin*:ti,ab,kw 0
#14 ("alm-14789" or "alm-14789r" or "alm-14789tm" or alm14789* or kx01* or "kx-01" or "kx-01r" or "kx-01tm" or kx2391* or "kx2-391" or "kx2-391r" or "kx2-391tm" or "kx-2391" or "kx-2391r" or "kx-2391tm" or "kx-2-391" or "kx-2-391r" or "kx-2-391tm"):ti,ab,kw 1
#15 ("1080645-95-9" or 4v9848rs5g or 84ac3796wj or "897016-82-9"):ti,ab,kw 0
#16 [mh ^Diclofenac] 1916
#17 diclofenac*:ti,ab,kw 5099
#18 (abitren* or aceclofenac* or benfofen* or dealgic* or deflamat* or delphinac* or dichlofenal* or dicloflex* or diclomax* or diclonate* or diclophenac* or diclophlogont* or dicloreum* or dicrofenac* or duravolten* or dyloject* or ecofenac* or effekton* or feloran* or gp45840* or "gp-45840" or "gp-45840r" or "gp-45840tm" or las41007* or "las-41007" or "las-41007r" or "las-41007tm" or lexobene* or neriodin* or novapirina* or orthofen* or orthophen* or ortofen* or pennsaid* or primofenac* or prophenatin* or rewodina* or rhumalgan* or solacutan* or solaraze* or "sr-38" or "sr-38r" or "sr-38tm" or sr38* or voldal* or voltaren* or voltarol* or xenid*):ti,ab,kw 639
#19 (cataflam* or d11ax18 or m01ab05 or m02aa15 or s01bc03):ti,ab,kw 22
#20 ("15307-79-6" or "239-346-4" or qtg126297q or 144o8ql0l1 or "15307-86-5" or "239-348-5" or "15307-81-0" or l4d5ua6cb4):ti,ab,kw 0
#21 [mh ^Fluorouracil] 4881
#22 (fluorouracil* or fluoro next uracil*):ti,ab,kw 10276
#23 (5fluoropyrimidin* or 5 next fluoropyrimidin* or 5fluoropyrimidine* or 5 next fluoropyrimidine* or 5fluorouracil* or 5fluoro next uracil* or 5fluoruracil* or 5fu* or 5 next fu* or 5hu next hexal* or 5 next hu next hexal* or accusite* or actikerall* or actikerell* or actino next hermal* or adrucil* or agicil* or arac* or carac* or cinkef or effluderm* or efudex* or efudix* or efurix* or emtricitabine next impurity or eurofluor* or f6627* or "f-6627" or "f-6627r" or "f-6627tm" or fivoflu* or flucytosine next impurity or fluoroblastin* or fluoroplex* or fluorouricil* or fluoruracil* or fluouracil* or fluoxan* or flurablastin* or fluracedyl* or fluracil* or fluracilium* or fluril* or fluroblastin* or fluroblastine* or fluro next uracil* or flurodex* or "haemato fu" or "haemato fur" or "haemato futm" or ifacil* or l01bc02* or neofluor* or "nsc-18913" or "nsc-18913r" or "nsc-18913tm" or "nsc-19893" or "nsc-19893r" or "nsc-19893tm" or nsc18913* or nsc19893* or oncofu* or onkofluor* or phthoruracil* or queroplex* or ribofluor* or "ro-2-9757" or "ro-2-9757r" or "ro-2-9757tm" or "ro2-9757" or "ro2-9757r" or "ro2-9757tm" or "ro-29757" or "ro-29757r" or "ro-29757tm" or ro29757* or tolak* or uflahex* or utoral* or verrumal*):ti,ab,kw 12230
#24 ("10318-20-4" or "200-085-6" or "51-21-8" or "57050-04-1" or "57172-36-8" or "68021-61-4" or u3p01618rt or 15fs54053u or "56177-80-1" or "260-029-1" or q0e5j9oq9o or "766-63-2" or pm476l7o8g or "14787-18-9"):ti,ab,kw 0
#25 [mh ^Imiquimod] 244

#26 imiquimod*:ti,ab,kw 523
#27 (aldara* or d06bb10 or imunocare* or "r-837" or "r-837r" or "r-837tm" or r837* or "s-26308" or "s-26308r" or "s-26308tm" or s26308* or zartra* or zyclara*):ti,ab,kw 82
#28 ("99011-02-6" or p1qw714r7m):ti,ab,kw 0
#29 (ingenol* next mebutate*):ti,ab,kw 114
#30 ("agn-204332" or "agn-204332r" or "agn-204332tm" or agn204332* or d06bx02 or ingenol next 3 next angelate* or ingenol next 3 next mebutate* or ingenol* next angelate* or "pep-005" or "pep-005r" or "pep-005tm" or pep005* or picato*):ti,ab,kw 35
#31 ("75567-37-2" or 7686s50jah):ti,ab,kw 0
#32 #13 or #14 or #15 or #16 or #17 or #18 or #19 or #20 or #21 or #22 or #23 or #24 or #25 or #26 or #27 or #28 or #29 or #30 or #31 25062
#33 [mh ^Cryotherapy] 661
#34 [mh "Cryosurgery"] 337
#35 (cryotherap* or cryo next therap* or cryotreat* or cryo next treat*):ti,ab,kw 2074
#36 (cryoablat* or cryo next ablat* or cryoapplication* or cryo next application* or cryoblat* or cryo next blat* or cryocoag* or cryo next coag* or cryodestr* or cryo next destr* or cryoenerg* or cryo next energ* or cryogen* or cryo next gen* or cryoisolat* or cryo next isolat* or cryosurg* or cryo next surg* or cryotherm* or cryo next therm*):ti,ab,kw909
#37 (freez* or froz*):ti,ab,kw 6273
#38 (liquid next (nitrogen or n2)):ti,ab,kw 328
#39 ln2:ti,ab,kw 20
#40 (cold next (treatment* or therap*)):ti,ab,kw 254
#41 #33 or #34 or #35 or #36 or #37 or #38 or #39 or #40 9084
#42 [mh ^phototherapy] or [mh ^"intense pulsed light therapy"] or [mh ^"low level light therapy"] or [mh photochemotherapy] 2552
#43 [mh ^"Photosensitizing Agents"]677
#44 (photodynamic* or photo next dynamic*):ti,ab,kw 2203
#45 (photochemotherap* or photo next chemotherap* or photoradiat* or photo next radiat* or photosensiti* or photo next sensiti* or phototherap* or photo next therap*):ti,ab,kw 4985
#46 chemophototherap*:ti,ab,kw 6
#47 (light next (treatment* or therap*)):ti,ab,kw 2165
#48 pdt:ti,ab,kw 1671
#49 #42 or #43 or #44 or #45 or #46 or #47 or #48 7947
#50 #12 and (#32 or #41 or #49) 822
#51 #50 in Cochrane Reviews, Cochrane Protocols 11

**Source: Health Technology Assessment Database (HTA Database)**
Interface / URL: https://www.crd.york.ac.uk/CRDWeb
Database coverage dates: Information not found. From 31 March 2018, the HTA database remains available, but CRD are no longer adding new records to it.
Search date: 25/06/20
Retrieved records: 18
Search strategy:

1 MeSH DESCRIPTOR Keratosis, Actinic 20
2 (actinic*) 35
3 (((solar* OR senil*) NEAR6 (keratos* OR keratoma* OR keratotic* OR keratopath*))) 4
4 (((keratos* OR keratoma* OR keratotic* OR keratopath*) NEAR6 (solar* OR senil*))) 1
5 (((solar* OR senil*) NEAR6 (cheilit* OR cheilos*))) 0
6 (((cheilit* OR cheilos*) NEAR6 (solar* OR senil*))) 0
7 ((hyperkeratos* OR hyperkeratoma* OR hyperkeratotic* OR hyperkeratopath*)) 8

| | | |
|---|---|---|
| 8 | ((keratinocytic intraepidermal neoplasia* OR keratinocytic intra-epidermal neoplasia*)) | 0 |
| 9 | (((sailor* OR farmer*) NEAR0 (lip OR lips))) | 0 |
| 10 | ((ak OR aks)) NOT ((ak OR aks)):AU | 47 |
| 11 | ((hak OR haks)) NOT ((hak OR haks)):AU | 0 |
| 12 | ((kin OR kins)) | 7 |
| 13 | #1 OR #2 OR #3 OR #4 OR #5 OR #6 OR #7 OR #8 OR #9 OR #10 OR #11 OR #12 | 86 |
| 14 | (#13) IN DARE | 27 |
| 15 | (#13) IN HTA | 18 |

**Source: Database of Abstracts of Reviews of Effects (DARE)**
Interface / URL: https://www.crd.york.ac.uk/CRDWeb
Database coverage dates: Information not found. Bibliographic records were published on DARE until 31st March 2015. Searches of MEDLINE, Embase, CINAHL, PsycINFO and PubMed were continued until the end of the 2014.
Search date: 25/06/20
Retrieved records: 27
Search strategy:

| | | |
|---|---|---|
| 1 | MeSH DESCRIPTOR Keratosis, Actinic | 20 |
| 2 | (actinic*) | 35 |
| 3 | (((solar* OR senil*) NEAR6 (keratos* OR keratoma* OR keratotic* OR keratopath*))) | 4 |
| 4 | (((keratos* OR keratoma* OR keratotic* OR keratopath*) NEAR6 (solar* OR senil*))) | 1 |
| 5 | (((solar* OR senil*) NEAR6 (cheilit* OR cheilos*))) | 0 |
| 6 | (((cheilit* OR cheilos*) NEAR6 (solar* OR senil*))) | 0 |
| 7 | ((hyperkeratos* OR hyperkeratoma* OR hyperkeratotic* OR hyperkeratopath*)) | 8 |
| 8 | ((keratinocytic intraepidermal neoplasia* OR keratinocytic intra-epidermal neoplasia*)) | 0 |
| 9 | (((sailor* OR farmer*) NEAR0 (lip OR lips))) | 0 |
| 10 | ((ak OR aks)) NOT ((ak OR aks)):AU | 47 |
| 11 | ((hak OR haks)) NOT ((hak OR haks)):AU | 0 |
| 12 | ((kin OR kins)) | 7 |
| 13 | #1 OR #2 OR #3 OR #4 OR #5 OR #6 OR #7 OR #8 OR #9 OR #10 OR #11 OR #12 | 86 |
| 14 | (#13) IN DARE | 27 |

**Source: PubMed**
Interface / URL: https://pubmed.ncbi.nlm.nih.gov/
Database coverage dates: Information not found
Search date: 26/06/20
Retrieved records: 60
Search strategy:

| | | |
|---|---|---|
| 65 | #63 NOT #64 | 60 |
| 64 | medline[sb] | 26,901,869 |
| 63 | #60 NOT (#61 OR #62) | 1,207 |
| 62 | ("news"[pt:noexp] OR "case reports"[pt:noexp]) OR case report[ti] | 2,348,994 |
| 61 | "animals"[mh] NOT "humans"[mh:noexp] | 4,711,847 |
| 60 | #50 AND #59 | 1,469 |
| 59 | #51 OR #52 OR #53 OR #54 OR #55 OR #56 OR #57 OR #58 | 4,793,879 |
| 58 | groups [tiab] | 2,085,731 |
| 57 | trial [tiab] | 600,325 |
| 56 | randomly [tiab] | 335,764 |
| 55 | drug therapy [sh] | 2,214,815 |

| | | |
|---|---|---|
| 54 | placebo [tiab] | 214,265 |
| 53 | randomized [tiab] | 523,207 |
| 52 | controlled clinical trial [pt] | 598,209 |
| 51 | randomized controlled trial [pt] | 509,336 |
| 50 | #12 AND (#32 OR #41 OR #49) | 2,312 |
| 49 | #42 OR #43 OR #44 OR #45 OR #46 OR #47 OR #48 | 74,295 |
| 48 | pdt[tiab] | 13,626 |
| 47 | (light treatment*[tiab] OR light therap*[tiab]) | 4,553 |
| 46 | chemophototherap*[tiab] | 81 |
| 45 | (photochemotherap*[tiab] OR photo-chemotherap*[tiab] OR photoradiat*[tiab] OR photo-radiat*[tiab] OR photosensiti*[tiab] OR photo-sensiti*[tiab] OR phototherap*[tiab] OR photo-therap*[tiab]) | 39,757 |
| 44 | (photodynamic*[tiab] OR photo-dynamic*[tiab]) | 25,159 |
| 43 | "Photosensitizing Agents"[mesh:noexp] | 15,945 |
| 42 | "phototherapy"[mesh:noexp] OR "intense pulsed light therapy"[mesh:noexp] OR "low-level light therapy"[mesh:noexp] OR "photochemotherapy"[mesh] | 33,699 |
| 41 | #33 OR #34 OR #35 OR #36 OR #37 OR #38 OR #39 OR #40 | 169,615 |
| 40 | (cold treatment*[tiab] OR cold therap*[tiab]) | 1,561 |
| 39 | ln2[tiab] | 804 |
| 38 | (liquid nitrogen[tiab] OR liquid n2[tiab]) | 8,523 |
| 37 | (freez*[tiab] OR froz*[tiab]) | 136,624 |
| 36 | (cryoablat*[tiab] OR cryo-ablat*[tiab] OR cryoapplication*[tiab] OR cryo-application*[tiab] OR cryoblat*[tiab] OR cryo-blat*[tiab] OR cryocoag*[tiab] OR cryo-coag*[tiab] OR cryodestr*[tiab] OR cryo-destr*[tiab] OR cryoenerg*[tiab] OR cryo-energ*[tiab] OR cryogen*[tiab] OR cryo-gen*[tiab] OR cryoisolat*[tiab] OR cryo-isolat*[tiab] OR cryosurg*[tiab] OR cryo-surg*[tiab] OR cryotherm*[tiab] OR cryo-therm*[tiab]) | 16,727 |
| 35 | (cryotherap*[tiab] OR cryo-therap*[tiab] OR cryotreat*[tiab] OR cryo-treat*[tiab]) | 7,565 |
| 34 | "Cryosurgery"[mesh:noexp] | 12,896 |
| 33 | "Cryotherapy"[mesh:noexp] | 5,085 |
| 32 | #13 OR #14 OR #15 OR #16 OR #17 OR #18 OR #19 OR #20 OR #21 OR #22 OR #23 OR #24 OR #25 OR #26 OR #27 OR #28 OR #29 OR #30 OR #31 | 154,978 |
| 31 | ((75567-37-2[tiab] OR 75567-37-2[rn] OR 75567-37-2[nm]) OR (7686s50jah[tiab] OR 7686s50jah[rn] OR 7686s50jah[nm])) | 0 |
| 30 | ((agn-204332*[tiab] OR agn-204332*[rn] OR agn-204332*[nm]) OR (agn204332*[tiab] OR agn204332*[rn] OR agn204332*[nm]) OR (d06bx02[tiab] OR d06bx02[rn] OR d06bx02[nm]) OR ((ingenol*[tiab] OR ingenol*[rn] OR ingenol*[nm]) AND (angelate*[tiab] OR angelate*[rn] OR angelate*[nm])) OR (pep-005[tiab] OR pep-005[rn] OR pep-005[nm]) OR (pep-005r[tiab] OR pep-005r[rn] OR pep-005r[nm]) OR (pep-005tm[tiab] OR pep-005tm[rn] OR pep-005tm[nm]) OR (pep005*[tiab] OR pep005*[rn] OR pep005*[nm]) OR (picato*[tiab] OR picato*[rn] OR picato*[nm])) | 258 |
| 29 | ((ingenol*[tiab] OR ingenol*[rn] OR ingenol*[nm]) AND (mebutate*[tiab] OR mebutate*[rn] OR mebutate*[nm])) | 316 |
| 28 | ((99011-02-6[tiab] OR 99011-02-6[rn] OR 99011-02-6[nm]) OR (p1qw714r7m[tiab] OR p1qw714r7m[rn] OR p1qw714r7m[nm])) | 2,486 |
| 27 | ((aldara*[tiab] OR aldara*[rn] OR aldara*[nm]) OR (d06bb10[tiab] OR d06bb10[rn] OR d06bb10[nm]) OR (imunocare*[tiab] OR imunocare*[rn] OR imunocare*[nm]) OR (r-837[tiab] OR r-837[rn] OR r-837[nm]) OR (r-837r[tiab] OR r-837r[rn] OR r-837r[nm]) OR (r-837tm[tiab] OR r-837tm[rn] OR r-837tm[nm]) OR (r837*[tiab] OR r837*[rn] OR r837*[nm]) OR (s-26308*[tiab] OR s-26308*[rn] OR s-26308*[nm]) OR (s26308*[tiab] OR s26308*[rn] OR s26308*[nm]) OR (zartra*[tiab] OR zartra*[rn] OR zartra*[nm]) OR (zyclara*[tiab] OR zyclara*[rn] OR zyclara*[nm])) | 349 |
| 26 | (imiquimod*[tiab] OR imiquimod*[rn] OR imiquimod*[nm]) | 3,741 |
| 25 | "Imiquimod"[mesh:noexp] | 2,486 |

24 ((10318-20-4[tiab] OR 10318-20-4[rn] OR 10318-20-4[nm]) OR (200-085-6[tiab] OR 200-085-6[rn] OR 200-085-6[nm]) OR (51-21-8[tiab] OR 51-21-8[rn] OR 51-21-8[nm]) OR (57050-04-1[tiab] OR 57050-04-1[rn] OR 57050-04-1[nm]) OR (57172-36-8[tiab] OR 57172-36-8[rn] OR 57172-36-8[nm]) OR (68021-61-4[tiab] OR 68021-61-4[rn] OR 68021-61-4[nm]) OR (u3p01618rt[tiab] OR u3p01618rt[rn] OR u3p01618rt[nm]) OR (15fs54053u[tiab] OR 15fs54053u[rn] OR 15fs54053u[nm]) OR (56177-80-1[tiab] OR 56177-80-1[rn] OR 56177-80-1[nm]) OR (260-029-1[tiab] OR 260-029-1[rn] OR 260-029-1[nm]) OR (q0e5j9oq9o[tiab] OR q0e5j9oq9o[rn] OR q0e5j9oq9o[nm]) OR (766-63-2[tiab] OR 766-63-2[rn] OR 766-63-2[nm]) OR (pm476l7o8g[tiab] OR pm476l7o8g[rn] OR pm476l7o8g[nm]) OR (14787-18-9[tiab] OR 14787-18-9[rn] OR 14787-18-9[nm])) 41,367

23 ((5fluoropyrimidin*[tiab] OR 5fluoropyrimidin*[rn] OR 5fluoropyrimidin*[nm]) OR (5-fluoropyrimidin*[tiab] OR 5-fluoropyrimidin*[rn] OR 5-fluoropyrimidin*[nm]) OR (5fluoropyrimidine*[tiab] OR 5fluoropyrimidine*[rn] OR 5fluoropyrimidine*[nm]) OR (5-fluoropyrimidine*[tiab] OR 5-fluoropyrimidine*[rn] OR 5-fluoropyrimidine*[nm]) OR (5fluorouracil*[tiab] OR 5fluorouracil*[rn] OR 5fluorouracil*[nm]) OR (5fluoro-uracil*[tiab] OR 5fluoro-uracil*[rn] OR 5fluoro-uracil*[nm]) OR (5fluoruracil*[tiab] OR 5fluoruracil*[rn] OR 5fluoruracil*[nm]) OR (5fu[tiab] OR 5fu[rn] OR 5fu[nm]) OR (5fur[tiab] OR 5fur[rn] OR 5fur[nm]) OR (5futm[tiab] OR 5futm[rn] OR 5futm[nm]) OR (5-fu[tiab] OR 5-fu[rn] OR 5-fu[nm]) OR (5-fur[tiab] OR 5-fur[rn] OR 5-fur[nm]) OR (5-futm[tiab] OR 5-futm[rn] OR 5-futm[nm]) OR (5hu[tiab] OR 5hu[rn] OR 5hu[nm]) OR (5-hu[tiab] OR 5-hu[rn] OR 5-hu[nm]) OR (accusite*[tiab] OR accusite*[rn] OR accusite*[nm]) OR (actikerall*[tiab] OR actikerall*[rn] OR actikerall*[nm]) OR (actikerell*[tiab] OR actikerell*[rn] OR actikerell*[nm]) OR (actino-hermal*[tiab] OR actino-hermal*[rn] OR actino-hermal*[nm]) OR (adrucil*[tiab] OR adrucil*[rn] OR adrucil*[nm]) OR (agicil*[tiab] OR agicil*[rn] OR agicil*[nm]) OR (arac*[tiab] OR arac*[rn] OR arac*[nm]) OR (carac*[tiab] OR carac*[rn] OR carac*[nm]) OR (cinkef[tiab] OR cinkef[rn] OR cinkef[nm]) OR (effluderm*[tiab] OR effluderm*[rn] OR effluderm*[nm]) OR (efudex*[tiab] OR efudex*[rn] OR efudex*[nm]) OR (efudix*[tiab] OR efudix*[rn] OR efudix*[nm]) OR (efurix*[tiab] OR efurix*[rn] OR efurix*[nm]) OR ((emtricitabine[tiab] OR emtricitabine[rn] OR emtricitabine[nm]) AND (impurity[tiab] OR impurity[rn] OR impurity[nm])) OR (eurofluor*[tiab] OR eurofluor*[rn] OR eurofluor*[nm]) OR (f6627*[tiab] OR f6627*[rn] OR f6627*[nm]) OR (f-6627*[tiab] OR f-6627*[rn] OR f-6627*[nm]) OR (fivoflu*[tiab] OR fivoflu*[rn] OR fivoflu*[nm]) OR ((flucytosine[tiab] OR flucytosine[rn] OR flucytosine[nm]) AND (impurity[tiab] OR impurity[rn] OR impurity[nm])) OR (fluoroblastin*[tiab] OR fluoroblastin*[rn] OR fluoroblastin*[nm]) OR (fluoroplex*[tiab] OR fluoroplex*[rn] OR fluoroplex*[nm]) OR (fluorouricil*[tiab] OR fluorouricil*[rn] OR fluorouricil*[nm]) OR (fluoruracil*[tiab] OR fluoruracil*[rn] OR fluoruracil*[nm]) OR (fluouracil*[tiab] OR fluouracil*[rn] OR fluouracil*[nm]) OR (fluoxan*[tiab] OR fluoxan*[rn] OR fluoxan*[nm]) OR (flurablastin*[tiab] OR flurablastin*[rn] OR flurablastin*[nm]) OR (fluracedyl*[tiab] OR fluracedyl*[rn] OR fluracedyl*[nm]) OR (fluracil*[tiab] OR fluracil*[rn] OR fluracil*[nm]) OR (fluracilium*[tiab] OR fluracilium*[rn] OR fluracilium*[nm]) OR (fluril*[tiab] OR fluril*[rn] OR fluril*[nm]) OR (fluroblastin*[tiab] OR fluroblastin*[rn] OR fluroblastin*[nm]) OR (fluroblastine*[tiab] OR fluroblastine*[rn] OR fluroblastine*[nm]) OR (fluro-uracil*[tiab] OR fluro-uracil*[rn] OR fluro-uracil*[nm]) OR (flurodex*[tiab] OR flurodex*[rn] OR flurodex*[nm]) OR (haemato-fu[tiab] OR haemato-fu[rn] OR haemato-fu[nm]) OR (haemato-fur[tiab] OR haemato-fur[rn] OR haemato-fur[nm]) OR (haemato-futm[tiab] OR haemato-futm[rn] OR haemato-futm[nm]) OR (ifacil*[tiab] OR ifacil*[rn] OR ifacil*[nm]) OR (l01bc02*[tiab] OR l01bc02*[rn] OR l01bc02*[nm]) OR (neofluor*[tiab] OR neofluor*[rn] OR neofluor*[nm]) OR (nsc-18913*[tiab] OR nsc-18913*[rn] OR nsc-18913*[nm]) OR (nsc-19893*[tiab] OR nsc-19893*[rn] OR nsc-19893*[nm]) OR (nsc18913*[tiab] OR nsc18913*[rn] OR nsc18913*[nm]) OR (nsc19893*[tiab] OR nsc19893*[rn] OR nsc19893*[nm]) OR (oncofu*[tiab] OR oncofu*[rn] OR oncofu*[nm]) OR (onkofluor*[tiab] OR onkofluor*[rn] OR onkofluor*[nm]) OR (phthoruracil*[tiab] OR phthoruracil*[rn] OR phthoruracil*[nm]) OR (queroplex*[tiab] OR queroplex*[rn] OR queroplex*[nm]) OR (ribofluor*[tiab] OR ribofluor*[rn] OR ribofluor*[nm]) OR (ro-2-9757*[tiab] OR ro-2-9757*[rn] OR ro-2-9757*[nm]) OR (ro2-9757*[tiab] OR ro2-9757*[rn] OR ro2-9757*[nm]) OR (ro-29757*[tiab] OR ro-29757*[rn] OR ro-29757*[nm]) OR (ro29757*[tiab] OR ro29757*[rn] OR ro29757*[nm]) OR (tolak*[tiab] OR tolak*[rn] OR

tolak*[nm]) OR (uflahex*[tiab] OR uflahex*[rn] OR uflahex*[nm]) OR (utoral*[tiab] OR utoral*[rn] OR utoral*[nm]) OR (verrumal*[tiab] OR verrumal*[rn] OR verrumal*[nm])) 119,134
22 ((fluorouracil*[tiab] OR fluorouracil*[rn] OR fluorouracil*[nm]) OR (fluoro-uracil*[tiab] OR fluoro-uracil*[rn] OR fluoro-uracil*[nm])) 54,144
21 "Fluorouracil"[mesh:noexp] 41,364
20 ((15307-79-6[tiab] OR 15307-79-6[rn] OR 15307-79-6[nm]) OR (239-346-4[tiab] OR 239-346-4[rn] OR 239-346-4[nm]) OR (qtg126297q[tiab] OR qtg126297q[rn] OR qtg126297q[nm]) OR (144o8ql0l1[tiab] OR 144o8ql0l1[rn] OR 144o8ql0l1[nm]) OR (15307-86-5[tiab] OR 15307-86-5[rn] OR 15307-86-5[nm]) OR (239-348-5[tiab] OR 239-348-5[rn] OR 239-348-5[nm]) OR (15307-81-0[tiab] OR 15307-81-0[rn] OR 15307-81-0[nm]) OR (l4d5ua6cb4[tiab] OR l4d5ua6cb4[rn] OR l4d5ua6cb4[nm])) 7,831
19 ((cataflam*[tiab] OR cataflam*[rn] OR cataflam*[nm]) OR (d11ax18[tiab] OR d11ax18[rn] OR d11ax18[nm]) OR (m01ab05[tiab] OR m01ab05[rn] OR m01ab05[nm]) OR (m02aa15[tiab] OR m02aa15[rn] OR m02aa15[nm]) OR (s01bc03[tiab] OR s01bc03[rn] OR s01bc03[nm])) 17
18 ((abitren*[tiab] OR abitren*[rn] OR abitren*[nm]) OR (aceclofenac*[tiab] OR aceclofenac*[rn] OR aceclofenac*[nm]) OR (benfofen*[tiab] OR benfofen*[rn] OR benfofen*[nm]) OR (dealgic*[tiab] OR dealgic*[rn] OR dealgic*[nm]) OR (deflamat*[tiab] OR deflamat*[rn] OR deflamat*[nm]) OR (delphinac*[tiab] OR delphinac*[rn] OR delphinac*[nm]) OR (dichlofenal*[tiab] OR dichlofenal*[rn] OR dichlofenal*[nm]) OR (dicloflex*[tiab] OR dicloflex*[rn] OR dicloflex*[nm]) OR (diclomax*[tiab] OR diclomax*[rn] OR diclomax*[nm]) OR (diclonate*[tiab] OR diclonate*[rn] OR diclonate*[nm]) OR (diclophenac*[tiab] OR diclophenac*[rn] OR diclophenac*[nm]) OR (diclophlogont*[tiab] OR diclophlogont*[rn] OR diclophlogont*[nm]) OR (dicloreum*[tiab] OR dicloreum*[rn] OR dicloreum*[nm]) OR (dicrofenac*[tiab] OR dicrofenac*[rn] OR dicrofenac*[nm]) OR (duravolten*[tiab] OR duravolten*[rn] OR duravolten*[nm]) OR (dyloject*[tiab] OR dyloject*[rn] OR dyloject*[nm]) OR (ecofenac*[tiab] OR ecofenac*[rn] OR ecofenac*[nm]) OR (effekton*[tiab] OR effekton*[rn] OR effekton*[nm]) OR (feloran*[tiab] OR feloran*[rn] OR feloran*[nm]) OR (gp45840*[tiab] OR gp45840*[rn] OR gp45840*[nm]) OR (gp-45840*[tiab] OR gp-45840*[rn] OR gp-45840*[nm]) OR (las41007*[tiab] OR las41007*[rn] OR las41007*[nm]) OR (las-41007*[tiab] OR las-41007*[rn] OR las-41007*[nm]) OR (lexobene*[tiab] OR lexobene*[rn] OR lexobene*[nm]) OR (neriodin*[tiab] OR neriodin*[rn] OR neriodin*[nm]) OR (novapirina*[tiab] OR novapirina*[rn] OR novapirina*[nm]) OR (orthofen*[tiab] OR orthofen*[rn] OR orthofen*[nm]) OR (orthophen*[tiab] OR orthophen*[rn] OR orthophen*[nm]) OR (ortofen*[tiab] OR ortofen*[rn] OR ortofen*[nm]) OR (pennsaid*[tiab] OR pennsaid*[rn] OR pennsaid*[nm]) OR (primofenac*[tiab] OR primofenac*[rn] OR primofenac*[nm]) OR (prophenatin*[tiab] OR prophenatin*[rn] OR prophenatin*[nm]) OR (rewodina*[tiab] OR rewodina*[rn] OR rewodina*[nm]) OR (rhumalgan*[tiab] OR rhumalgan*[rn] OR rhumalgan*[nm]) OR (solacutan*[tiab] OR solacutan*[rn] OR solacutan*[nm]) OR (solaraze*[tiab] OR solaraze*[rn] OR solaraze*[nm]) OR (sr-38[tiab] OR sr-38[rn] OR sr-38[nm]) OR (sr-38r[tiab] OR sr-38r[rn] OR sr-38r[nm]) OR (sr-38tm[tiab] OR sr-38tm[rn] OR sr-38tm[nm]) OR (sr38*[tiab] OR sr38*[rn] OR sr38*[nm]) OR (voldal*[tiab] OR voldal*[rn] OR voldal*[nm]) OR (voltaren*[tiab] OR voltaren*[rn] OR voltaren*[nm]) OR (voltarol*[tiab] OR voltarol*[rn] OR voltarol*[nm]) OR (xenid*[tiab] OR xenid*[rn] OR xenid*[nm])) 1,572
17 (diclofenac*[tiab] OR diclofenac*[rn] OR diclofenac*[nm]) 13,155
16 "Diclofenac"[mesh:noexp] 7,820
15 ((1080645-95-9[tiab] OR 1080645-95-9[rn] OR 1080645-95-9[nm]) OR (4v9848rs5g[tiab] OR 4v9848rs5g[rn] OR 4v9848rs5g[nm]) OR (84ac3796wj[tiab] OR 84ac3796wj[rn] OR 84ac3796wj[nm]) OR (897016-82-9[tiab] OR 897016-82-9[rn] OR 897016-82-9[nm])) 0
14 ((alm-14789*[tiab] OR alm-14789*[rn] OR alm-14789*[nm]) OR (alm14789*[tiab] OR alm14789*[rn] OR alm14789*[nm]) OR (kx01*[tiab] OR kx01*[rn] OR kx01*[nm]) OR (kx-01[tiab] OR kx-01[rn] OR kx-01[nm]) OR (kx-01r[tiab] OR kx-01r[rn] OR kx-01r[nm]) OR (kx-01tm[tiab] OR kx-01tm[rn] OR kx-01tm[nm]) OR (kx2391*[tiab] OR kx2391*[rn] OR kx2391*[nm]) OR (kx2-391[tiab] OR kx2-391[rn] OR kx2-391[nm]) OR (kx2-391r[tiab] OR kx2-391r[rn] OR kx2-391r[nm]) OR (kx2-391tm[tiab] OR kx2-391tm[rn] OR kx2-391tm[nm]) OR (kx-2391*[tiab] OR kx-2391*[rn] OR kx-2391*[nm]) OR (kx-2-391[tiab]

OR kx-2-391[rn] OR kx-2-391[nm]) OR (kx-2-391r[tiab] OR kx-2-391r[rn] OR kx-2-391r[nm]) OR (kx-2-391tm[tiab] OR kx-2-391tm[rn] OR kx-2-391tm[nm])) 32
13 (tirbanibulin*[tiab] OR tirbanibulin*[rn] OR tirbanibulin*[nm]) 2
12 #1 OR #2 OR #3 OR #4 OR #5 OR #6 OR #7 OR #8 OR #9 OR #10 OR #11 23,379
11 (kin[tiab] or kins[tiab]) 6,658
10 (hak[tiab] or haks[tiab]) 206
9 (ak[tiab] OR aks[tiab]) 5,852
8 ((sailor*[tiab] OR farmer*[tiab]) AND (lip[tiab] OR lips[tiab])) 79
7 (keratinocytic[tiab] AND neoplasia*[tiab]) 27
6 (hyperkeratos*[tiab] OR hyperkeratoma*[tiab] OR hyperkeratotic*[tiab] OR hyperkeratopath*[tiab]) 6,778
5 ((solar*[tiab] OR senil*[tiab]) AND (cheilit*[tiab] OR cheilos*[tiab])) 42
4 ((solar*[tiab] OR senil*[tiab]) AND (keratos*[tiab] OR keratoma*[tiab] OR keratotic*[tiab] OR keratopath*[tiab])) 847
3 (actinic*[tiab] AND (cheilit*[tiab] OR cheilos*[tiab])) 353
2 (actinic*[tiab] AND (keratos*[tiab] OR keratoma*[tiab] OR keratotic*[tiab] OR keratopath*[tiab])) 3,714
1 "Keratosis, Actinic"[mesh:noexp] 2,050

**Source: Epistemonikos**
Interface / URL: https://www.epistemonikos.org/
Database coverage dates: Information not found.
Search date: 26/06/20
Retrieved records: 181
Search strategy:

The following two searches were entered into the search box at: https://www.epistemonikos.org/en/advanced_search. Search 1 was not limited by field

The searches were restricted by category to systematic reviews.

All results were imported into an empty EndNote Library (212 records in total). Records were then de-duplicated using EndNote default deduplication settings. 31 records were identified as duplicates and removed. The remaining 181 records were retrieved.

1. ((actinic* OR solar* OR senil*) AND (keratos* OR keratoma* OR keratotic* OR keratopath* OR cheilit* OR cheilos*)) OR (hyperkeratos* OR hyperkeratoma* OR hyperkeratotic* OR hyperkeratopath* OR keratinocytic) OR ((sailor* OR farmer*) AND (lip OR lips)) = 122

2. (title:(ak OR aks OR hak OR haks OR kin OR kins) OR abstract:(ak OR aks OR hak OR haks OR kin OR kins)) = 90

**Source: ClinicalTrials.gov**
Interface / URL: https://clinicaltrials.gov/ct2/home
Database coverage dates: Information not found. ClinicalTrials.gov was created as a result of the Food and Drug Administration Modernization Act of 1997 (FDAMA). The site was made available to the public in February 2000.
Search date: 06/07/20
Retrieved records: 418
Search strategy:

The following three searches were conducted separately. All search terms were entered using the Expert search interface.

The three sets of results were imported into an empty EndNote library (470 records) and deduplicated using EndNote default de-duplication settings. 52 records were identified as duplicates and removed from the EndNote library. The remaining 418 records were retrieved for assessment.

1. (actinic OR solar OR senile) AND (keratosis OR keratoses OR keratoma OR keratomas OR keratotic OR keratopathy OR keratopathies OR keratopathic OR cheilitis OR cheilosis OR cheiloses OR hyperkeratosis OR hyperkeratoses OR hyperkeratoma OR hyperkeratomas OR hyperkeratotic OR hyperkeratopathy OR hyperkeratopathies OR hyperkeratopathic OR "keratinocytic intraepidermal neoplasia" OR "keratinocytic intraepidermal neoplasias" OR "keratinocytic intra-epidermal neoplasia" OR "keratinocytic intra-epidermal neoplasias") = 336

2. ("sailor lip" OR "sailors lip" OR "sailor lips" OR "sailors lips" OR "farmer lip" OR "farmers lip" OR "farmer lips" OR "farmers lips") = 0

3. AREA[ConditionSearch] (ak OR aks OR hak OR haks OR kin OR kins) = 134

Search note:

- in clinicaltrials.gov, a search on "keratinocytic intraepidermal neoplasias" also searches on the term keratosis (the latter is identified by the interface as a synonym). This search for this term (and variants) was therefore restricted to records which also explicitly referred to the actinic, solar or senile context.

- in clinicaltrials.gov, a search on hyperkeratosis also searches on the term keratosis (the latter is identified by the interface as a synonym). This search for this term was therefore restricted to records which also explicitly referred to the actinic, solar or senile context.

- Abbreviation such as 'AK' are frequently found in non-relevant contexts in clinicaltrials.gov records. The search for the following terms was therefore restricted to the 'Condition or disease' field: ak OR aks OR hak OR haks OR kin OR kins

**Source: EU Clinical Trials Register**
Interface / URL: https://www.clinicaltrialsregister.eu/
Database coverage dates: The EU Clinical Trials Register contains information on interventional clinical trials on medicines conducted in the European Union (EU), or the European Economic Area (EEA) which started after 1 May 2004.
Search date: 08/07/20
Retrieved records: 448 (246 trials were identified and downloaded in 'Full Trial Details' format. Files in this format contain full details on each clinical trial selected for download. Where multi-state trials have been downloaded full information for each of the member states/countries involved in the trial are included separately. When imported into EndNote, records are created for each multi-state trial. When details for the 246 trials were imported into EndNote, 448 records were created. The figure has been used for the 'number of retrieved records' figure, and for informing PRISMA)
Search strategy:

The following terms were entered using the search interface at https://www.clinicaltrialsregister.eu/ctr-search/search:

((actinic OR solar OR senile) AND (keratosis OR keratoses OR keratoma OR keratomas OR keratotic OR keratopathy OR keratopathies OR keratopathic OR cheilitis OR cheilosis OR cheiloses)) OR (hyperkeratosis OR hyperkeratoses OR hyperkeratoma OR hyperkeratomas OR hyperkeratotic OR hyperkeratopathy OR hyperkeratopathies OR hyperkeratopathic OR "keratinocytic intraepidermal neoplasia" OR "keratinocytic intraepidermal neoplasias" OR "keratinocytic intra-epidermal neoplasia" OR "keratinocytic intra-epidermal neoplasias" OR "sailor lip" OR "sailors lip" OR "sailor lips" OR "sailors lips" OR "farmer lip" OR "farmers lip" OR "farmer lips" OR "farmers lips" OR ak OR aks OR hak OR haks OR kin OR kins) = 246 trials found (246 trials with a EudraCT protocol found. 0 paediatric studies in scope of Art45 of the Paediatric Regulation found).

**Source: Drugs@FDA**
Interface / URL: https://www.accessdata.fda.gov/scripts/cder/daf/index.cfm
Database coverage dates: 1938 to present
Search date: 08/07/20
Retrieved records: 32
Search strategy:

Note: Drugs@FDA does not allow searching by indication.

The following documents were sought for the drugs of interest in AK: Medical Reviews, Statistical Reviews, Other Reviews.

The drugs of interest are:

- Tirbanibulin 1%
- Diclofenac 3%
- Imiquimod 3.75% or 5%
- Ingenol mebutate 0.015%
- Salicylic acid + 5-fluorouracil 0.5%
- 5-fluorouracil 5%
- 5-fluorouracil 4%
- aminolevulinic acid (as used in photodynamic therapy (PDT))
- methyl aminolevulinate (as used in PDT)

The search interface at the following URL was used:
https://www.accessdata.fda.gov/scripts/cder/daf/index.cfm

Separate searches were conducted on each drug name shown below.

For each search:
- Each returned drug name was selected to expand the result
- Each of the drugs shown on expansion was selected to display details
- The link to 'Approval Date(s) and History, Letters, Labels, Reviews' was selected (if available) to view documents
- Documents were checked to ensure the results was in the correct indication and contained at least one of the relevant reviews listed above
- PDFs for Medical reviews, Statistical Reviews or Other Reviews on the correct indication were retrieved for further assessment

Injectable and oral treatments were excluded.

1. tirbanibulin = 0 results returned
2. diclofenac = 5 documents retrieved
3. imiquimod = 7 documents downloaded
4. ingenol mebutate = 4 documents downloaded
5. salicylic acid = 0 documents retrieved
6. fluorouracil = 8 documents downloaded
7. 5-fu = 0 results returned
8. 5fu = 0 results returned
9. aminolevulinate = 2 documents downloaded
10. aminolaevulinate = 0 results returned
11. aminolaevulinic = 0 results returned
12. aminolevulinic = 6 documents downloaded

32 documents were retrieved

**Source: European Medicines Agency (EMA) medicines webpages**
Interface / URL: https://www.ema.europa.eu/en
Database coverage dates: n/a
Search date: 14/07/20
Retrieved records: 8
Search strategy:

The following document was sought for the interventions of interest in AK: 'EPAR - Public Assessment Report'. 'Assessment Report' documents with variant titles were also retrieved (e.g. 'EPAR - Assessment Report', 'EPAR - Assessment Report – Variation', 'EPAR – Refusal Assessment Report' and 'EPAR - Assessment Report – Article [#]', 'EPAR - Assessment Report – Extension' etc.)
Documents found under both 'Initial marketing-authorisation documents' and ' Changes since initial authorisation of medicine' were retrieved.

The search interface at the following URL was used: https://www.ema.europa.eu/en/medicines. The following searches were conducted separately:

actinic* AND kerato* = 21 results returned
actinic* AND cheil* = 1 result returned
solar* AND kerato* = 7 results returned
solar* AND cheil* = 3 results returned
senil* AND kerato* = 1 result returned
senil* AND cheil* = 0 results returned
hyperkerato* = 58 results returned
"keratinocytic intraepidermal neoplasia" = 0 results returned
"keratinocytic intraepidermal neoplasias" = 0 results returned
"keratinocytic intra-epidermal neoplasia" = 0 results returned
"keratinocytic intra-epidermal neoplasias" = 0 results returned

Results were restricted by applying the following filters (where available):
Categories: Human
Medicine: European public assessment reports (EPAR).

Each result was assessed for correct drug and correct indication.

For relevant results, documents under 'Assessment history' were checked to identify 'EPAR - Public Assessment Report' documents. PDFs for relevant EPARs were retrieved for further assessment. Duplicate documents were not retrieved.

8 documents were retrieved

**Source: National Institute for Health and Care Excellence (NICE) webpages**
Interface / URL: https://www.nice.org.uk/
Database coverage dates: n/a
Search date: 14/07/20
Retrieved records: 0
Search strategy:

The following documents were sought for the interventions of interest in AK: Company Submissions, Final Appraisal Determination Documents and Assessment Reports (the latter only if the NICE technology appraisal was a MTA - multiple technology appraisal).

The site-wide search interface was used at: https://www.nice.org.uk/. Separate searches were conducted on the terms below.

Results were filtered by 'Document Type' to 'Guidance', then by 'Guidance Category' to 'Technology appraisal guidance', 'Interventional procedures guidance' or 'Medical technologies guidance'.

Results were scanned to identify technology appraisals, interventional procedures guidance or medical technologies guidance for the correct interventions in the correct indication. Associated documents under the 'History' tab were viewed to check for Company Submissions, Final Appraisal Determination Documents and Assessment Reports.

PDFs for relevant documents were retrieved for further assessment. Duplicate documents were not retrieved.

1. keratosis OR keratoses OR keratoma OR keratomas = 2 results returned
2. keratotic OR keratopathy OR keratopathies OR keratopathic = 0 results returned
3. cheilitis OR cheilosis OR cheiloses = 0 results returned
4. hyperkeratosis OR hyperkeratoses OR hyperkeratoma OR hyperkeratomas = 2 results returned
5. hyperkeratotic OR hyperkeratopathy OR hyperkeratopathies OR hyperkeratopathic = 1 result returned
6. keratinocytic = 0 results returned

0 documents were retrieved

**Source: Canadian Agency for Drugs and Technologies in Health (CADTH) webpages**
Interface / URL: https://www.cadth.ca/
Database coverage dates: n/a
Search date: 15/07/20
Retrieved records: 4
Search strategy:

The following documents were sought for the drugs of interest in AK: CADTH clinical guidance and CADTH final recommendations.

The site-wide search interface was used at: https://www.cadth.ca/. Separate searches were conducted on the search terms shown below.

Results were assessed for correct intervention and correct indication. Relevant results were checked for document types of interest.

PDFs of relevant documents were retrieved for further assessment. Duplicate documents were not retrieved.

1. keratosis = 20 results returned
2. keratoses = 11 results returned
3. keratoma = 0 results returned
4. keratomas = 0 results returned
5. keratotic = 1 result returned
6. keratopathy = 6 results returned
7. keratopathies = 1 result returned
8. keratopathic = 0 results returned
9. cheilitis = 4 results returned
10. cheilosis = 0 results returned
11. cheiloses = 0 results returned
12. hyperkeratosis = 8 results returned
13. hyperkeratoses = 0 results returned
14. hyperkeratoma = 0 results returned
15. hyperkeratomas = 0 results returned
16. hyperkeratotic = 0 results returned
17. hyperkeratopathy = 0 results returned
18. hyperkeratopathies = 0 results returned
19. hyperkeratopathic = 0 results returned
20. keratinocytic = 0 results returned

4 documents were retrieved

**Source: Institute for Clinical and Economic Review webpages**
Interface / URL: https://icer-review.org/
Database coverage dates: n/a
Search date: 15/07/20
Retrieved records: 0
Search strategy:

The following were sought for the drugs of interest in weight loss: Final Evidence Report (or Draft Evidence Report if Final was not available) and the Evidence Presentation (if available).

'Past topics' were located at the following URL: https://icer-review.org/topics/. The "Filter by Topic" drop down was used to identify reviews in AK. No relevant reviews were identified

0 documents were retrieved

**Source: European Association of Dermato-Oncology (EADO) Congress 2018**
Interface / URL: n/a
Database coverage dates: n/a
Search date: 17/07/20

Retrieved records: 0
Search strategy:

A link to the 2018 14th EADO Congress was identified at: https://www.eado.org/activities/eado-congress/8. The link was: http://www.congresseado-melanomacenters2018.com/. The link led to a blank page.

Abstracts could not be identified freely available online.

EADO were contacted (info@eado.org) to enquire if abstracts were available to be freely viewed online anywhere or, if not, whether a PDF of the abstracts could be sent. EADO confirmed that the abstracts are available only to congress participants.

Almirall were asked if they could provide access to the abstracts. Almirall investigated, but were unable to access the EADO 2018 abstracts.

The decision was made by research team that the EADO 2018 abstracts were unobtainable

**Source: European Association of Dermato-Oncology (EADO) Congress 2019**
Interface / URL: n/a
Database coverage dates: n/a
Search date: 02/09/20
Retrieved records: 0
Search strategy:

Abstracts could not be identified freely available online.

EADO were contacted (info@eado.org) to enquire if abstracts were available to be freely viewed online anywhere or, if not, whether a PDF of the abstracts could be sent. EADO confirmed that the abstracts are available only to congress participants.

Almirall were asked if they could provide access to the abstracts. Almirall investigated and a PDF of the abstract book for EADO 2019 was provided.

Ctrl+F was used to search on the following terms across the 15th EADO Congress Abstract Book. Each term was searched on individually. Abstracts including the term were assessed by the Information Specialist for relevance. Potentially relevant abstracts were retrieved for further consideration:

actinic = 0 abstracts retrieved
solar = 0 abstracts retrieved
senil = 0 abstracts retrieved
hyperkerato = 0 abstracts retrieved
keratinocytic = 0 abstracts retrieved
sailor = 0 abstracts retrieved
farmer = 0 abstracts retrieved
ak = 0 abstracts retrieved
kin = 0 abstracts retrieved

0 abstracts were retrieved for further consideration

**Source: European Academy of Dermatology and Venereology (EADV) Congress 2017**
Interface / URL: n/a
Database coverage dates: n/a
Search date: 05/08/20
Retrieved records: 6
Search strategy:

The list of EADV Congresses was identified at: https://www.eadv.org/congress/show-congress. No link was given for a 2017 Congress webpage, and no link could be found elsewhere on the website. Abstracts could not be identified freely available online.

The conference organisers were contacted (congress@eadv.org) to enquire if abstracts were available to be freely viewed online anywhere or, if not, whether a PDF of the abstracts could be sent. EADV confirmed that abstracts submitted for previous EADV events are freely accessible only to EADV members.

Almirall were asked if they could provide access to the abstracts. Almirall investigated, and abstracts were sent.

Two routes of access were provided:

1. EADV Abstracts.exe application. This route only facilitated search by topic browsing, or by title, author or abstract id search – no abstract search was available. The topic browsing and title-only search options were not considered to be robust enough search approaches, so this application was not used.

2. A 'data' folder containing 2493 individual abstracts as PDF files

The following process was used to search across the PDF files contained in the 'data' folder:

1. The first PDF file in the 'data' folder was opened in Acrobat Reader
2. Shift+Ctrl+F was used to to open the Search panel
3. The option to search 'All PDF documents in' was selected.
4. The dropdown option was used to select 'Browse for Location'
5. Navigated to the 'data folder' containing the conference abstract PDFs and clicked OK
6. The following terms were entered into 'What word or phrase would you like to search for?' box: actinic solar senil keratinocytic sailor farmer
7. 'Show more options' was selected
8. From the dropdown options, 'Match Any of the words' was selected
9. Clicked on 'Search'.
10. 112 PDFs were found containing the terms
11. Each displayed PDF result was clicked on to open the PDF. The PDF was assessed for relevance by the Information Specialist. PDFs for RCTs or systematic reviews on treatment of AK with the interventions of interest were retrieved for further consideration.
12. Following assessment, 6 / 112 PDFs were retrieved for further consideration
13. 'New search' was selected
14. The following terms were entered into 'What word or phrase would you like to search for?' box: ak aks hak haks kin kins
15. 'Whole words only' was selected
16. Clicked on 'Search'
17. 25 PDFs were found containing the terms

18. Each displayed PDF result was clicked on to open the PDF. The PDF was assessed for relevance by the Information Specialist. PDFs for RCTs or systematic reviews on treatment of AK with the interventions of interest were retrieved for further consideration. Duplicates were not retrieved.
19. Following assessment, 0 / 25 PDFs were retrieved for further consideration

Search note on hyperkeratosis terms:

Search functionality was very limited and did not facilitate efficient search and assessment methods. In this context, and given that the population of interest was AK rather than non-specific hyperkeratosis, in relation to hyperkeratosis terms the search approach used was designed to only retrieve records containing hyperkeratosis terms (and variants including hyperkeratoma, hyperkeratotic, hyperkeratopathy) if the terms were found in the context of 'actinic', 'solar' or 'senile'.

**Source: European Academy of Dermatology and Venereology (EADV) Congress 2018**
Interface / URL: n/a
Database coverage dates: n/a
Search date: 06/08/20
Retrieved records: 2
Search strategy:

The list of EADV Congresses was identified at: https://www.eadv.org/congress/show-congress

A link was found for a 2018 Congress webpage (https://eadvparis2018.org/) but the link did not lead to any congress information. No link could be found elsewhere on the website. Abstracts could not be identified freely available online.

The conference organisers were contacted (congress@eadv.org) to enquire if abstracts were available to be freely viewed online anywhere or, if not, whether a PDF of the abstracts could be sent. EADV confirmed that abstracts submitted for previous EADV events are freely accessible only to EADV members.

Almirall were asked if they could provide access to the abstracts. Almirall investigated, and abstracts were sent.

Two routes of access were provided:

1. EADV Abstracts.exe application. This route only facilitated search by topic browsing, or by title, author or abstract id search – no abstract search was available. The topic browsing and title-only search options were not considered to be robust enough search approaches, so this application was not used.

2. A 'data' folder containing 2543 individual abstracts as PDF files

The following process was used to search across the PDF files contained in the 'data' folder:

1. The first PDF file in the 'data' folder was opened in Acrobat Reader
2. Shift+Ctrl+F was used to to open the Search panel
3. The option to search 'All PDF documents in' was selected.
4. The dropdown option was used to select 'Browse for Location'
5. Navigated to the 'data folder' containing the conference abstract PDFs and clicked OK
6. The following terms were entered into 'What word or phrase would you like to search for?' box:
actinic solar senil keratinocytic sailor farmer

7. 'Show more options' was selected
8. From the dropdown options, 'Match Any of the words' was selected
9. Clicked on 'Search'.
10. 117 PDFs were found containing the terms
11. Each displayed PDF result was clicked on to open the PDF. The PDF was assessed for relevance by the Information Specialist. PDFs for RCTs or systematic reviews on treatment of AK with the interventions of interest were retrieved for further consideration.
12. Following assessment, 2 / 117 PDFs were retrieved for further consideration
13. 'New search' was selected
14. The following terms were entered into 'What word or phrase would you like to search for?' box: ak aks hak haks kin kins
15. 'Whole words only' was selected
16. Clicked on 'Search'
17. 20 PDFs were found containing the terms
18. Each displayed PDF result was clicked on to open the PDF. The PDF was assessed for relevance by the Information Specialist. PDFs for RCTs or systematic reviews on treatment of AK with the interventions of interest were retrieved for further consideration. Duplicates were not retrieved.
19. Following assessment, 0 / 20 PDFs were retrieved for further consideration

Search note on hyperkeratosis terms:

Search functionality was very limited and did not facilitate efficient search and assessment methods. In this context, and given that the population of interest was AK rather than non-specific hyperkeratosis, in relation to hyperkeratosis terms the search approach used was designed to only retrieve records containing hyperkeratosis terms (and variants including hyperkeratoma, hyperkeratotic, hyperkeratopathy) if the terms were found in the context of 'actinic', 'solar' or 'senile'.

**Source: European Academy of Dermatology and Venereology (EADV) Congress 2019**
Interface / URL: n/a
Database coverage dates: n/a
Search date: 06/08/20
Retrieved records: 1
Search strategy:

The list of EADV Congresses was identified at: https://www.eadv.org/congress/show-congress

A link was found for a 2019 Congress webpage (https://eadvmadrid2019.org/). No full abstracts could be found at this site. Abstracts could not be identified freely available online.

The conference organisers were contacted (congress@eadv.org) to enquire if abstracts were available to be freely viewed online anywhere or, if not, whether a PDF of the abstracts could be sent. EADV confirmed that abstracts submitted for previous EADV events are freely accessible only to EADV members.

Almirall were asked if they could provide access to the abstracts. Almirall investigated, and abstracts were sent.

Two routes of access were provided:

1. EADV Abstracts.exe application. This route only facilitated search by topic browsing, or by title, author or abstract id search – no abstract search was available. The topic browsing and title-only search options were not considered to be robust enough search approaches, so this application was not used.

2. A 'data' folder containing 2302 individual abstracts as PDF files

The following process was used to search across the PDF files contained in the 'data' folder:

1. The first PDF file in the 'data' folder was opened in Acrobat Reader
2. Shift+Ctrl+F was used to to open the Search panel
3. The option to search 'All PDF documents in' was selected.
4. The dropdown option was used to select 'Browse for Location'
5. Navigated to the 'data folder' containing the conference abstract PDFs and clicked OK
6. The following terms were entered into 'What word or phrase would you like to search for?' box: actinic solar senil keratinocytic sailor farmer
7. 'Show more options' was selected
8. From the dropdown options, 'Match Any of the words' was selected
9. Clicked on 'Search'.
10. 78 PDFs were found containing the terms
11. Each displayed PDF result was clicked on to open the PDF. The PDF was assessed for relevance by the Information Specialist. PDFs for RCTs or systematic reviews on treatment of AK with the interventions of interest were retrieved for further consideration.
12. Following assessment, 1 / 78 PDFs were retrieved for further consideration
13. 'New search' was selected
14. The following terms were entered into 'What word or phrase would you like to search for?' box: ak aks hak haks kin kins
15. 'Whole words only' was selected
16. Clicked on 'Search'
17. 14 PDFs were found containing the terms
18. Each displayed PDF result was clicked on to open the PDF. The PDF was assessed for relevance by the Information Specialist. PDFs for RCTs or systematic reviews on treatment of AK with the interventions of interest were retrieved for further consideration. Duplicates were not retrieved.
19. Following assessment, 0 / 14 PDFs were retrieved for further consideration

Search note on hyperkeratosis terms:

Search functionality was very limited and did not facilitate efficient search and assessment methods. In this context, and given that the population of interest was AK rather than non-specific hyperkeratosis, in relation to hyperkeratosis terms the search approach used was designed to only retrieve records containing hyperkeratosis terms (and variants including hyperkeratoma, hyperkeratotic, hyperkeratopathy) if the terms were found in the context of 'actinic', 'solar' or 'senile'.

## Supplementary Table S1: Databases and information sources searched for the review

| Resource | Interface / URL |
|---|---|
| MEDLINE(R) ALL | Ovid SP |
| PubMed | http://www.ncbi.nlm.nih.gov/pubmed |
| Embase | OvidSP |
| Cochrane Database of Systematic Reviews (CDSR) | Cochrane Library/Wiley |
| Cochrane Central Register of Controlled Trials (CENTRAL) | Cochrane Library/Wiley |
| Database of Abstracts of Reviews of Effects (DARE) | https://www.crd.york.ac.uk/CRDWeb/ |
| Health Technology Assessment Database (HTA Database) | https://www.crd.york.ac.uk/CRDWeb/ |
| Epistemonikos | https://www.epistemonikos.org/ |
| EU Clinical Trials Register | https://www.clinicaltrialsregister.eu/ |
| ClinicalTrials.gov | https://www.clinicaltrials.gov/ct |
| Drugs@FDA | https://www.accessdata.fda.gov/scripts/cder/daf/index.cfm |
| European Medicines Agency (EMA) medicines webpages | https://www.ema.europa.eu/en |
| National Institute for Health and Care Excellence (NICE) webpages | https://www.nice.org.uk/ |
| Canadian Agency for Drugs and Technologies in Health (CADTH) webpages | https://www.cadth.ca/ |
| Institute for Clinical and Economic Review (ICER) webpages | https://icer-review.org/ |

Recent research published as conference abstracts was identified by searching Embase (which indexes a significant number of conference publications). The following three conferences were identified as highly relevant:

- European Association of Dermato-Oncology (EADO) Congress
- American Academy of Dermatology (AAD) Annual Meeting
- European Academy of Dermatology and Venereology (EADV) Congress

We ascertained if records for these conferences (above) were included in Embase for the last three years (2017 to 2020). Records were not found in Embase for the sub-set of conferences listed below. For the conferences listed below, we undertook hand-searching of proceedings via conference webpages or journal supplements, where they were available online free of charge.

- EADV Congress: 2017, 2018, 2019
- EADO Congress: 2018, 2019

All were hand-searched, except EADO 2018. Abstracts were unobtainable for EADO 2018 – the abstracts could not be identified via an online search, and EADO confirmed that the abstracts were only available to conference attendees. AAD Annual Meeting 2020 was cancelled, and while EADO Congress 2020 and EADV Congress 2020 were held virtually in October 2020,

both were held after the review and NMA were completed, and for this reason are not included in this manuscript.

## Supplementary Table S2: Documents excluded from the review (145)

| Reference | Exclusion Reason |
|---|---|
| Actavis Inc. A Study Comparing Diclofenac Sodium Gel 3% to Solaraze® (Diclofenac Sodium) Gel 3% in the Treatment of Actinic Keratosis. Identifier: NCT01962987. In: ClinicalTrials.gov [internet]. Bethesda: US National Library of Medicine: 2013. Available from https://ClinicalTrials.gov/show/NCT01962987. | Completed trial (no results) |
| Actavis Inc. Bioequivalence of Generic Imiquimod Cream, 5% When Compared to Aldara™ (Imiquimod) Cream, 5% in the Treatment of Actinic Keratosis. Identifier: NCT00948428. In: ClinicalTrials.gov [internet]. Bethesda: US National Library of Medicine: 2008. Available from https://ClinicalTrials.gov/show/NCT00948428. | Completed trial (no results) |
| Actavis Mid-Atlantic LLC. A Bioequivalence Study With Clinical Endpoints Comparing Generic Imiquimod Cream, 3.75% and Zyclara™ (Imiquimod) Cream, 3.75% in Subjects With Actinic Keratoses. Identifier: NCT01502020. In: ClinicalTrials.gov [internet]. Bethesda: US National Library of Medicine: 2011. Available from https://ClinicalTrials.gov/show/NCT01502020. | Completed trial (no results) |
| Almirall SA. Study on the Efficacy of LAS41005 in the Treatment of Actinic Keratosis. Identifier: NCT00987246. In: ClinicalTrials.gov [internet]. Bethesda: US National Library of Medicine: 2008. Available from https://ClinicalTrials.gov/show/NCT00987246. | Completed trial (no results) |
| Apotex Inc. A Randomized, Double-Blind, Parallel-Group, Vehicle-Controlled Therapeutic Equivalence Study of Three Imiquimod Cream 5% Treatments for Patients With Actinic Keratosis. Identifier: NCT00859105. In: ClinicalTrials.gov [internet]. Bethesda: US National Library of Medicine: 2008. Available from https://ClinicalTrials.gov/show/NCT00859105. | Completed trial (no results) |
| Biofrontera Bioscience GmbH. A Clinical Trial of Topical Photodynamic Therapy With 5-aminolevulinic Acid for the Treatment of Actinic Keratosis. Identifier: NCT02799030. In: ClinicalTrials.gov [internet]. Bethesda: US National Library of Medicine: 2006. Available from https://ClinicalTrials.gov/show/NCT02799030. | Possible ongoing trial (no results) |
| Braathen L. Photodynamic therapy for oncologic indications: actinic keratosis (Abstract W20-3). 11th congress of the european academy of dermatology & venereology prague october 2nd-6th, 2002. 11th congress of the european academy of dermatology & venereology prague october 2nd-6th, 2002. 2002; 16: 57. Available from: https://www.cochranelibrary.com/central/doi/10.1002/central/CN-00478479/full | Abstract - cannot link to full text paper |
| Charité. A randomized study of topical 3% Diclofenac in a 2.5% Hyaluronate base (Solaraze® 3% Gel) versus topical 5% 5-Fluorouracil (Efudix® Cream) versus liquid nitrogen spray cryotherapy in immunosufficient patients with actinic keratoses – a single center study H2H Solaraze-5FU-Cryo. Identifier: EUCTR2006-000815-24. In: EU Clinical Trials Register [internet]. London: European Medicines Agency: 2006. Available from https://www.clinicaltrialsregister.eu/ctr-search/trial/2006-000815-24/DE/. | Completed trial (no results) |
| Dartmouth-Hitchcock Medical Center. Indoor Daylight Photo Dynamic Therapy for Actinic Keratosis. Identifier: NCT03805737. In: ClinicalTrials.gov [internet]. Bethesda: US National Library of Medicine: 2019. Available from https://ClinicalTrials.gov/show/NCT03805737. | Possible ongoing trial (no results) |
| Dermapharm AG. Double-blind, randomised clinical study comparing efficacy and safety of Imiquimod 5% Cream (Test) vs. Aldara® 5% Cream (Reference) vs. | Possible ongoing trial (no results) |

| Reference | Exclusion Reason |
|---|---|
| Vehicle in patients with actinic keratosis. Identifier: EUCTR2016-000712-15. In: EU Clinical Trials Register [internet]. London: European Medicines Agency: 2016. Available from https://www.clinicaltrialsregister.eu/ctr-search/trial/2016-000712-15/DE/. | |
| DUSA Pharmaceuticals I. Microneedle Lesion Preparation Prior to Aminolevulinic Acid Photodynamic Therapy (ALA-PDT) for AK on Face. Identifier: NCT02632110. In: ClinicalTrials.gov [internet]. Bethesda: US National Library of Medicine: 2016. Available from https://ClinicalTrials.gov/show/NCT02632110. | Ineligible study design |
| DUSA Pharmaceuticals I. Phase 3 Study of Levulan With New Blue Light for AK on the Face or Scalp. Identifier: NCT03024060. In: ClinicalTrials.gov [internet]. Bethesda: US National Library of Medicine: 2017. Available from https://ClinicalTrials.gov/show/NCT03024060. | Possible ongoing trial (no results) |
| Encube Ethicals Pvt. Ltd. Therapeutic Equivalence of Diclofenac Sodium Gel 3% vs Solaraze ® in the Treatment of Actinic Keratosis. Identifier: NCT03826550. In: ClinicalTrials.gov [internet]. Bethesda: US National Library of Medicine: 2018. Available from https://ClinicalTrials.gov/show/NCT03826550. | Possible ongoing trial (no results) |
| Foley K, Gupta A, Martin G, Tweed J, Villanueva E, Carviel J. Topical treatments and photodynamic therapy for actinic keratosis of the face and scalp. Cochrane Database Syst Rev. 2019; (10): Available from: http://dx.doi.org/10.1002/14651858.CD013452 | Ineligible publication type |
| Galderma R&D. A Study to Assess Recurrence of Actinic Keratosis in Participants Treated With Methyl Aminolevulinate Hydrochloride Cream or Vehicle Cream Who Achieved Complete Response to Treated Lesions in Earlier Study. Identifier: NCT04269395. In: ClinicalTrials.gov [internet]. Bethesda: US National Library of Medicine: 2020. Available from https://ClinicalTrials.gov/show/NCT04269395. | Possible ongoing trial (no results) |
| Galderma R&D. Multicenter Study to Assess the Efficacy and Safety of Methyl Aminolevulinate Hydrochloride (MAL) 16.8% Cream (CD06809-41) Versus Vehicle Cream for Actinic Keratosis of the Face. Identifier: NCT04085367. In: ClinicalTrials.gov [internet]. Bethesda: US National Library of Medicine: 2019. Available from https://ClinicalTrials.gov/show/NCT04085367. | Possible ongoing trial (no results) |
| Galderma. Metvix PDT Versus Vehicle PDT With Aktilite CL128 Lamp in Patients With Actinic Keratosis on the Face and Scalp. Identifier: NCT00304239. In: ClinicalTrials.gov [internet]. Bethesda: US National Library of Medicine: 2006. Available from https://ClinicalTrials.gov/show/NCT00304239. | Completed trial (no results) |
| Glynis Ablon1 AB, Suzanne Bruce3, Michael Bukhalo4, Seth Forman5, Abel Jarell6, Steven Kempers7,, Edward Lain8 RL, Jane Fang10, 1 The Ablon Skin Institute Research Center MB, United States, 2 Oregon Medical Research Center,, Portland US, 3 The Center For Skin Research, Suzanne Bruce And Associates, Pa, Houston, United States,, 4 Arlington Dermatology AH, United States, 5 Forward Clinical Trials, Tampa, United States, 6 Activmed, Practices & Research P, United States, 7 Associated Skin Care Specialists, Fridley, United States, 8 Austin, et al. Abstract N°: FC05.06. Title: KX2-391 ointment 1%, a novel Src phosphorylation and tubulin polymerisation inhibitor, for the treatment of actinic keratosis: Results from two pivotal Phase III studies. In: European Academy of Dermatology and Venereology (EADV) Congress 2019, 2019. | Abstract - cannot link to full text paper |
| Grönroos M. Daylight-PDT in the treatment of actinic keratosis: a pilot study comparing two different light sensitizers. Identifier: EUCTR2013-001389-40. In: EU Clinical Trials Register [internet]. London: European Medicines Agency: 2013. | Possible ongoing trial (no results) |

| Reference | Exclusion Reason |
|---|---|
| Available from https://www.clinicaltrialsregister.eu/ctr-search/trial/2013-001389-40/FI/. | |
| Grönroos M. Treating actinic keratoses with natural daylight PDT: comparing two light sensitizers (ALA vs. MAL). Identifier: EUCTR2013-002108-15. In: EU Clinical Trials Register [internet]. London: European Medicines Agency: 2013. Available from https://www.clinicaltrialsregister.eu/ctr-search/trial/2013-002108-15/FI/. | Possible ongoing trial (no results) |
| Infectopharm Arzneimittel GmbH. Study on the treatment of actinic keratosis with the medical device Solcera in comparison with the drug Solaraze and placebo. Identifier: EUCTR2019-003678-16. In: EU Clinical Trials Register [internet]. London: European Medicines Agency: 2019. Available from https://www.clinicaltrialsregister.eu/ctr-search/trial/2019-003678-16/DE/. | Possible ongoing trial (no results) |
| Intas Pharmaceuticals Ltd. A Randomized, Double-Blind, Placebo-controlled, Three-arm, Parallel Assignment, Multi-Centre, Therapeutic Equivalence Study of Two Fluorouracil 5% Topical Cream Formulations in Adult Patients with Multiple Actinic Keratoses Lesions. Identifier: EUCTr2018-003436-74. In: EU Clinical Trials Register [internet]. London: European Medicines Agency: 2018. Available from https://www.clinicaltrialsregister.eu/ctr-search/trial/2018-003436-74/ES/. | Possible ongoing trial (no results) |
| Janne Räsänen. Daylight photodynamic therapy for actinic keratoses: a multicentre study comparing two photosensitizers (BF-200 ALA versus MAL) Identifier: EUCTR2015-000265-32. In: EU Clinical Trials Register [internet]. London: European Medicines Agency: 2015. Available from https://www.clinicaltrialsregister.eu/ctr-search/trial/2015-000265-32/FI/. | Possible ongoing trial (no results) |
| LEO Pharma. Risk of Squamous Cell Carcinoma on Skin Areas Treated with Ingenol Mebutate Gel, 0.015% and Imiquimod Cream, 5%. Identifier: EUCTR2012-003112-31. In: EU Clinical Trials Register [internet]. London: European Medicines Agency: 2020. Available from https://www.clinicaltrialsregister.eu/ctr-search/trial/2012-003112-31/GB/. | Possible ongoing trial (no results) |
| LEO Pharma. Risk of Squamous Cell Carcinoma on Skin Areas Treated With Ingenol Mebutate Gel, 0.015% and Imiquimod Cream, 5%. Identifier: NCT01926496. In: ClinicalTrials.gov [internet]. Bethesda: US National Library of Medicine: 2013. Available from https://ClinicalTrials.gov/show/NCT01926496. | Possible ongoing trial (no results) |
| Maastricht Universitair Medisch Centrum. Topical Ingenol mebutate versus 5% 5-fluorouracil versus 5% Imiquimod versus photodynamic therapy in the treatment of actinic keratosis: a multi-center randomized efficacy and cost-effectiveness study. Identifier: EUCTR2014-003691-23. In: EU Clinical Trials Register [internet]. London: European Medicines Agency: 2014. Available from https://www.clinicaltrialsregister.eu/ctr-search/trial/2014-003691-23/NL/. | Possible ongoing trial (no results) |
| Medicis Global Service Corporation. Potential Effect of Topical Imiquimod on Atrial Ectopy in Patients With Actinic Keratosis. Identifier: NCT01413763. In: ClinicalTrials.gov [internet]. Bethesda: US National Library of Medicine: 2011. Available from https://ClinicalTrials.gov/show/NCT01413763. | Completed trial (no results) |
| Novartis Pharmaceuticals. Safety, tolerability, and efficacy study of LFX453 in actinic keratosis patients. Identifier: NCT02404389. In: ClinicalTrials.gov [internet]. Bethesda: US National Library of Medicine: 2015. Available from https://ClinicalTrials.gov/show/NCT02404389. | Insufficient information to include |
| Perrigo Company. To Compare Safety and Efficacy of Perrigo's Drug Compared to an FDA Approved Drug in the Treatment of Actinic Keratosis. Identifier: | Completed trial (no results) |

| Reference | Exclusion Reason |
|---|---|
| NCT02459795. In: ClinicalTrials.gov [internet]. Bethesda: US National Library of Medicine: 2015. Available from https://ClinicalTrials.gov/show/NCT02459795. | |
| Perrigo Company. To Compare Safety and Efficacy of Perrigo's Drug Product Compared to an FDA Approved Drug Product in the Treatment of Actinic Keratosis. Identifier: NCT02385318. In: ClinicalTrials.gov [internet]. Bethesda: US National Library of Medicine: 2015. Available from https://ClinicalTrials.gov/show/NCT02385318. | Possible ongoing trial (no results) |
| PhotoCure ASA. A multicenter, double blind, vehicle-controlled, randomized study of photodynamic therapy (PDT) with Metvix®160 mg/g cream and Aktilite CL128 LED light in patients with multiple actinic keratoses on the face and/or scalp. Identifier: EUCTR2005-005015-13. In: EU Clinical Trials Register [internet]. London: European Medicines Agency: 2005. Available from https://www.clinicaltrialsregister.eu/ctr-search/trial/2005-005015-13/DE/. | Completed trial (no results) |
| Photonamic GmbH & Co. KG. PD P 506 A-PDT Versus Placebo-PDT and Cryosurgery for the Treatment of AK. Identifier: NCT00308867. In: ClinicalTrials.gov [internet]. Bethesda: US National Library of Medicine: 2006. Available from https://ClinicalTrials.gov/show/NCT00308867. | Completed trial (no results) |
| Photonamic GmbH & Co. KG. Photodynamic Therapy With PD P 506 A Compared With Placebo-PDT for the Treatment of AK. Identifier: NCT00308854. In: ClinicalTrials.gov [internet]. Bethesda: US National Library of Medicine: 2006. Available from https://ClinicalTrials.gov/show/NCT00308854. | Completed trial (no results) |
| Photonamic GmbH & Co. KG. Photodynamic Therapy with PD P 506 A or its placebo compared with cryosurgery for the treatment of mild to moderate actinic keratosis. Identifier: EUCTR2005-003556-36. In: EU Clinical Trials Register [internet]. London: European Medicines Agency: 2005. Available from https://www.clinicaltrialsregister.eu/ctr-search/trial/2005-003556-36/DE/. | Completed trial (no results) |
| RWTH Aachen University Hospital. Prospektive randomisierte studie zur untersuchung der wirksamkeit der photodynamischen therapie bei aktinischen keratosen PDT in AK. Identifier: EUCTR2005-002983-27. In: EU Clinical Trials Register [internet]. London: European Medicines Agency: 2005. Available from https://www.clinicaltrialsregister.eu/ctr-search/trial/2005-002983-27/DE/. | Ineligible comparator |
| Skroza N, Bernardini N, Proietti I, Potenza C. Clinical utility of ingenol mebutate in the management of actinic keratosis: perspectives from clinical practice. Ther Clin Risk Manag. 2018;14:1879-85. | SR for reference checking |
| Sol-Gel Technologies Ltd. Bioequivalence Study to Compare Sol-Gel Technologies' 5-FU Cream With Efudex® (5-FU) Cream. Identifier: NCT03727074. In: ClinicalTrials.gov [internet]. Bethesda: US National Library of Medicine: 2018. Available from https://ClinicalTrials.gov/show/NCT03727074. | Possible ongoing trial (no results) |
| Szeimies R, Karrer S, Radakovic-Fijan S, Tanew A, Calzavara-Pinton P, Zane C, et al. Photodynamic therapy using topical methyl aminolaevulinate photodynamic therapy compared with cryotherapy for actinic keratosis: a European prospective, randomized study. Abstract P-32 The 85th BAD Annual Meeting 5-8th July 2005, Glasgow, UK. Br J Dermatol. 2005; 153(S1): 30. Available from: https://onlinelibrary.wiley.com/doi/epdf/10.1111/j.1365-2133.2005.06628.x | Ineligible patient population |
| Taro Pharmaceuticals USA. A Study Comparing Diclofenac Sodium Gel 3% to Solaraze® Gel 3% in the Treatment of Actinic Keratosis. Identifier: NCT01742663. In: ClinicalTrials.gov [internet]. Bethesda: US National Library of Medicine: 2012. Available from https://ClinicalTrials.gov/show/NCT01742663. | Completed trial (no results) |

| Reference | Exclusion Reason |
|---|---|
| Taro Pharmaceuticals USA. Clinical Study to Evaluate the Bioequivalence of Two Imiquimod Cream 3.75% Formulations in Patients With Actinic Keratosis. Identifier: NCT01788007. In: ClinicalTrials.gov [internet]. Bethesda: US National Library of Medicine: 2013. Available from https://ClinicalTrials.gov/show/NCT01788007. | Completed trial (no results) |
| Teva Pharmaceuticals USA. A Randomized, Double-Blind, Parallel-Group, Vehicle-Controlled, Multicenter Study Comparing Diclofenac Sodium Gel, 3% to Solaraze® Gel, 3% in the Treatment of Actinic Keratosis on the Face or Bald Scalp. Identifier: NCT02611804. In: ClinicalTrials.gov [internet]. Bethesda: US National Library of Medicine: 2015. Available from https://ClinicalTrials.gov/show/NCT02611804. | Possible ongoing trial (no results) |
| Teva Pharmaceuticals USA. Study Comparing Imiquimod Cream, 3.75% to Zyclara® (Imiquimod) Cream, 3.75% in the Treatment of Actinic Keratosis. Identifier: NCT01686152. In: ClinicalTrials.gov [internet]. Bethesda: US National Library of Medicine: 2012. Available from https://ClinicalTrials.gov/show/NCT01686152. | Completed trial (no results) |
| Torres A, Anders R. Double-blind, vehicle-controlled study to evaluate the clinical, histological and confocal microscopy effects during 4 weeks of treatment for actinic keratoses with imiquimod 5 % cream Abstract 263. The 65th Annual Meeting of the Society for Investigative Dermatology, April 28th -May 1st, 2004, Providence, Rhode Island,USA. J Invest Dermatol. 2004; 122(3): A44. Available from: https://www.jidonline.org/action/showPdf?pii=S0022-202X%2815%2930735-1 | Abstract - cannot link to full text paper |
| University Hospital Regensburg. Daylight-PDT With MAL for AK and Photodamaged Skin. Identifier: NCT02736760. In: ClinicalTrials.gov [internet]. Bethesda: US National Library of Medicine: 2016. Available from https://ClinicalTrials.gov/show/NCT02736760. | Possible ongoing trial (no results) |
| Yamauchi P, Lowe N, Lask G, Patnaik R, Moore D, Foley P. Methyl aminolevulinate and photodynamic therapy in the treatment of actinic keratosis (Abstract 804). The 63rd Annual Meeting of the Society for Investigative Dermatology, 15-18 May, Los Angeles, USA. J Invest Dermatol. 2002; 119(1): 341. Available from: https://www.cochranelibrary.com/central/doi/10.1002/central/CN-00487928/full | Ineligible outcomes |
| Zouboulis CC. Is cryosurgery less effective that conservative regimens in the treatment of actinic keratoses? Journal of the European Academy of Dermatology and Venereology : JEADV. 2016;30(10):e50-e53. | SR for reference checking |
| Akarsu S, Aktan S, Atahan A, Koc P, Ozkan S. Comparison of topical 3% diclofenac sodium gel and 5% imiquimod cream for the treatment of actinic keratoses. Clin Exp Dermatol. 2011;36(5):479-84. | Ineligible patient population |
| Alomar A, Stockfleth E, Dirschka T, Gupta G, Aractingi S, Dakovic R, et al. Efficacy and safety of imiquimod 3.75% from lmax in actinic keratosis according to fitzpatrick skin type. J Drugs Dermatol. 2016;15(3):285-9. | Ineligible subgroup analysis |
| Anderson SJ, Steinman HK, Mazzurco JD, Dixon AJ. Prolonged AEs following photodynamic therapy: regulatory implications. J Drugs Dermatol. 2014;13(1):62-66. | Ineligible patient population |
| Augustin M, Tu JH, Knudsen KM, Erntoft S, Larsson T, Hanke CW. Ingenol mebutate gel for actinic keratosis: the link between quality of life, treatment satisfaction, and clinical outcomes. J Am Acad Dermatol. 2015;72(5):816-21. | Ineligible outcomes |

| Reference | Exclusion Reason |
|---|---|
| Berman B, Goldenberg G, Hanke CW, Tyring S, Werschler WP, Knudsen KM, et al. Long-term efficacy and clearance of actinic keratosis using sequential treatment with ingenol mebutate 0 015% gel after cryosurgery. Br J Dermatol. 2014;171(S4):48. | Ineligible intervention |
| Berman B, Goldenberg G, Hanke CW, Tyring SK, Werschler WP, Knudsen KM, et al. Ingenol mebutate after cryosurgery is effective for actinic keratosis. J Dtsch Dermatol Ges. 2014;12(S3):11. | Ineligible intervention |
| Berman B, Marmur E, Melgaard A, Larsson T. Three-day topical treatment with ingenol mebutate gel, 0.015% for actinic keratoses on the face and scalp: analysis of data pooled from two trials. J Dtsch Dermatol Ges. 2013;11(S2):109. | Abstract - cannot link to full text paper |
| Berman B, Melgaard A, Marmur E, Larsson T. Three-day topical treatment with ingenol mebutate gel, 0.015% for actinic keratoses on the face and scalp: analysis of data pooled from two trials. J Am Acad Dermatol. 2012;66(4 S1):AB3. | Abstract - cannot link to full text paper |
| Berman B, Shabbir AQ, MacNeil T, Knudsen KM. Variables in cryosurgery technique associated with clearance of actinic keratosis. Dermatol Surg. 2017;43(3):424-30. | Ineligible intervention |
| Burkhart CN, Burkhart CG. Reassessment of topical diclofenac/hyaluronan gel for actinic keratoses. Int J Dermatol. 2002;41(6):371-2. | Ineligible publication type |
| Calzavara-Pinton P, Zane C, Pacou M, Szeimies RM. Bucher's indirect comparison of daylight photodynamic therapy with methyl aminolevulinate cream versus diclofenac plus hyaluronic acid gel for the treatment of multiple actinic keratosis. Eur J Dermatol. 2016;26(5):487-92. | SR for reference checking |
| Calzavara-Pinton P, Zane C, Pacou M, Szeimies RM. Daylight photodynamic therapy with methyl aminolevulinate cream versus diclofenac plus hyaluronic acid gel for the treatment of multiple actinic keratoses: an indirect comparison. Melanoma Res. 2016;26(S1):e80. | Abstract - cannot link to full text paper |
| Dermapharm AG. Clinical study to compare two creams with the active substance imiquimod and one cream without active substance for patients with actinic keratosis. Identifier: EUCTR2016-000712-15-DE. In: EU Clinical Trials Register [internet]. London: European Medicines Agency: 2016. Available from https://www.clinicaltrialsregister.eu/ctr-search/trial/2016-000712-15/DE. | Possible ongoing trial (no results) |
| Dessinioti C, Panagiotopoulos A, Verra P, Papagiannaki N, Stavropoulos P, Katsambas A, et al. A randomized, double-blind, vehicle-controlled study of diclofenac 3% in hyaluronic acid 2.5% gel for the treatment of actinic keratoses. Exp Dermatol. 2013;22(3):e12. | Ineligible patient population |
| Effron JS, Aliazzi H, Garcia-Zuazaga J. Current evidence and applications of photodynamic therapy in dermatology: part 1: cutaneous neoplasms. Journal of the Dermatology Nurses' Association. 2015;7(3):145-51. | Review, non-systematic |
| Epstein E. Evidence-based evaluation of photodynamic therapy of actinic keratoses. J Am Acad Dermatol. 2003;48(2):304; author reply 04-6. | Ineligible publication type |
| Fariba I, Ali A, Hossein SA, Atefeh S, Atarzadeh Behbahan SA. Efficacy of 3% diclofenac gel for the treatment of actinic keratoses: a randomized, double-blind, placebo controlled study. Indian J Dermatol Venereol Leprol. 2006;72(5):346-9. | Ineligible study design |
| Fitzmaurice S, Eisen DB. Daylight photodynamic therapy: what is known and what is yet to be determined. Dermatol Surg. 2016;42(3):286-95. | SR for reference checking |
| Fu C, Kuang BH, Qin L, Zeng XY, Wang BC. Efficacy and safety of photodynamic therapy with amino-5-laevulinate nanoemulsion versus methyl-5-aminolaevulinate for actinic keratosis: a meta-analysis. Photodiagnosis Photodyn Ther. 2019;27:408-14. | SR for reference checking |

| Reference | Exclusion Reason |
|---|---|
| Garbe C, Basset-Seguin N, Poulin Y, Larsson T, Knudsen KM, Lear J. Safety of follow-up field application of ingenol mebutate 0.015% gel for actinic keratoses on face and scalp. J Am Acad Dermatol. 2015;72(5 S1):AB191. | Ineligible study design |
| Garbe C, Basset-Seguin N, Poulin Y, Larsson T, Osterdal ML, Venkata R, et al. Efficacy and safety of follow-up field treatment of actinic keratosis with ingenol mebutate 0.015% gel: a randomized, controlled 12-month study. Br J Dermatol. 2016;174(3):505-13. | Ineligible study design |
| Garbe C, Larsson T, Venkata R, Knudsen KM, Lear J, Seguin NB, et al. Ingenol mebutate 0.015% gel follow-on use for multiple actinic keratoses on face and scalp: a randomized 12-month phase 3 study. J Clin Aesthet Dermatol. 2015;8(5 S1):S7-S8. | Ineligible study design |
| Gebauer K, Brown P, Varigos G. Topical diclofenac in hyaluronan gel for the treatment of solar keratoses. Australas J Dermatol. 2003;44(1):40-3. | Ineligible patient population |
| Gholam P, Bosselmann I, Enk A, Fink C. Impact of red versus blue light on tolerability and efficacy of PDT: a randomized controlled trial. J Dtsch Dermatol Ges. 2018;16(6):711-17. | Ineligible study design |
| Goldberg D, Ciocon D, Hussain M. Comparison of 5-ALA alone, PDT alone and a combination of sequential 5-ALA and PDT in the treatment of actinic keratoses and photorejuvenation. Lasers Surg Med. 2010;42(S22):92. | Ineligible outcomes |
| Goldenberg G, Smith CC, Martin G, Del Rosso J. Imiquimod 2.5% and 3.75% for the treatment of actinic keratosis: analysis by subgroup. J Am Acad Dermatol. 2014;70(5 S1):AB127. | Abstract - cannot link to full text paper |
| Gollnick H, Dirschka T, Ostendorf R, Kerl H, Kunstfeld R. Long-term clinical outcomes of imiquimod 5% cream vs. diclofenac 3% gel for actinic keratosis on the face or scalp: a pooled analysis of two randomized controlled trials. J Eur Acad Dermatol Venereol. 2020;34(1):82-89. | Duplicate |
| Gupta G, Stockfleth E, Peris K, Aractingi S, Alomar A, Dakovic R, et al. Long-term sustained lesion clearance from Lmax with imiquimod 3.75%, a new field-directed treatment for actinic keratosis. J Eur Acad Dermatol Venereol. 2015;29(9):1840-2. | Ineligible study design |
| Gutierrez Garcia-Rodrigo C, Pellegrini C, Piccioni A, Maini M, Fargnoli MC. Long-term efficacy data for daylight-PDT. G Ital Dermatol Venereol. 2018;153(6):800-05. | SR for reference checking |
| Haddad A, Santos ID, Gragnani A, Ferreira LM. The effect of increasing fluence on the treatment of actinic keratosis and photodamage by photodynamic therapy with 5-aminolevulinic acid and intense pulsed light. Photomed Laser Surg. 2011;29(6):427-32. | Ineligible study design |
| Haedersdal M, Erlendsson AM, Paasch U, Anderson RR. Translational medicine in the field of ablative fractional laser (AFXL)-assisted drug delivery: a critical review from basics to current clinical status. J Am Acad Dermatol. 2016;74(5):981-1004. | SR for reference checking |
| Hanke CW, Albrecht L, Skov T, Larsson T, Osterdal ML, Spelman L. Efficacy and safety of ingenol mebutate gel in field treatment of actinic keratosis on full face, balding scalp, or approximately 250 $cm^2$ on the chest: a phase 3 randomized controlled trial. J Am Acad Dermatol. 2020;82(3):642-50. | Ineligible intervention |
| Hanke CW, Swanson N, Bruce S, Berman B, Kulp J, Levy S. Complete clearance is sustained for at least 12 months after treatment of actinic keratoses of the face or balding scalp via daily dosing with imiquimod 3.75% or 2.5% cream. J Drugs Dermatol. 2011;10(2):165-70. | Ineligible subgroup analysis |

| Reference | Exclusion Reason |
|---|---|
| Ibbotson S, Stones R, Bowling J, Campbell S, Kownacki S, Sivaramakrishnan M, et al. A consensus on the use of daylight photodynamic therapy in the UK. J Dermatolog Treat. 2017;28(4):360-67. | Ineligible study design |
| Intas Pharmaceuticals Ltd. A study to evaluate if a new cream of Fluorouracil 5% for treatment of actinic keratoses lesions is equivalent to an existing one that also contains Fluorouracil 5%. In: EU Clinical Trials Register [internet]. London: European Medicines Agency: 2019. Available from https://www.clinicaltrialsregister.eu/ctr-search/trial/2018-003436-74/ES. | Possible ongoing trial (no results) |
| Janne Räsänen. Daylight photodynamic therapy for premalignant skin cancer lesions: a comparison of two light sensitizing creams. Identifier: EUCTR2015-000265-32-FI. In: EU Clinical Trials Register [internet]. London: European Medicines Agency: 2015. Available from https://www.clinicaltrialsregister.eu/ctr-search/trial/2015-000265-32/FI. | Possible ongoing trial (no results) |
| Jansen MHE, Kessels J, Merks I, Nelemans PJ, Kelleners-Smeets NWJ, Mosterd K, et al. A trial-based cost-effectiveness analysis of topical 5-fluorouracil vs. imiquimod vs. ingenol mebutate vs. methyl aminolaevulinate conventional photodynamic therapy for the treatment of actinic keratosis in the head and neck area performed in the Netherlands. Br J Dermatol. 2020;21:21. | Ineligible outcomes |
| Jim On S, Knudsen KM, Skov T, Lebwohl M. Regression analysis of local skin reactions to predict clearance of actinic keratosis on the face in patients treated with ingenol mebutate gel: experience from randomized controlled trials. J Drugs Dermatol. 2017;16(2):112-14. | Does not report comparative results |
| Jorizzo J, Stewart D, Bucko A, Davis SA, Espy P, Hino P, et al. Randomized trial evaluating a new 0.5% fluorouracil formulation demonstrates efficacy after 1-, 2-, or 4-week treatment in patients with actinic keratosis. Cutis. 2002;70(6):335-9. | Ineligible intervention |
| Jorizzo J, Weiss J, Furst K, VandePol C, Levy SF. Effect of a 1-week treatment with 0.5% topical fluorouracil on occurrence of actinic keratosis after cryosurgery: a randomized, vehicle-controlled clinical trial. Arch Dermatol. 2004;140(7):813-6. | Ineligible intervention |
| Kolde G. Follow-up analysis of the efficacy of photodynamic therapy in actinic keratosis. Br J Dermatol. 2013;169(5):1156. | Ineligible publication type |
| Krawtchenko N, Roewert-Huber J, Ulrich M, Mann I, Sterry W, Stockfleth E. A randomised study of topical 5% imiquimod vs. topical 5-fluorouracil vs. cryosurgery in immunocompetent patients with actinic keratoses: a comparison of clinical and histological outcomes including 1-year follow-up. Br J Dermatol. 2007;157 Suppl 2:34-40. | Ineligible patient population |
| Lebwohl M, Berman B, Rigel D, Kaur M. Imiquimod 2.5% and 3.75% for the treatment of actinic keratosis: assessment of safety in 479 randomized patients. J Am Acad Dermatol. 2014;70(5 S1):AB127. | Abstract - cannot link to full text paper |
| Lebwohl M, Melgaard A, Kobayashi K, Swanson N. Local skin responses associated with ingenol mebutate gel for the treatment of actinic keratosis: two analyses of pooled data. J Am Acad Dermatol. 2012;66(4 S1):AB153. | Abstract - cannot link to full text paper |
| Lebwohl M, Shumack S, Stein Gold L, Melgaard A, Larsson T, Tyring SK. Long-term follow-up study of ingenol mebutate gel for the treatment of actinic keratoses. JAMA Dermatol. 2013;149(6):666-70. | Ineligible study design |
| Lebwohl M. Actinic keratosis. Jama. 2016;315(13):1394-5. | Ineligible publication type |
| Lee PK, Harwell WB, Loven KH, Phillips TJ, Whiting DA, Andres KL, et al. Long-term clinical outcomes following treatment of actinic keratosis with imiquimod 5% cream. Dermatol Surg. 2005;31(6):659-64. | Ineligible study design |

| Reference | Exclusion Reason |
|---|---|
| Maastricht Universitair Medisch Centrum. Comparative study for the treatment of actinic keratosis: 5% fluorouracil versus Imiquimod versus photodynamic therapy versus ingenol mebutate. Identifier: EUCTR2014-003691-23. In: EU Clinical Trials Register [internet]. London: European Medicines Agency: 2014. Available from https://www.clinicaltrialsregister.eu/ctr-search/trial/2014-003691-23/NL. | Possible ongoing trial (no results) |
| Maastricht University Medical Center. Treatment of Actinic keratosis: topical ingenol mebutate versus 5%5-fluorouracil versus 5% imiquimod versus photodynamic therapy. Identifier: NTR4849. In: Nederlands Trial Register [internet]. Amsterdam: The Dutch Cochrane Centre: 2014. Available from https://www.trialregister.nl/trial/4595. | Ineligible patient population |
| Malvehy J, Puig S. Response to 'Phase IV head-to-head randomized controlled trial comparing ingenol mebutate 0.015% gel with diclofenac sodium 3% gel for the treatment of actinic keratosis on the face or scalp'. Br J Dermatol. 2018;178(3):812-13. | Ineligible publication type |
| McEwan LE, Smith JG. Topical diclofenac/hyaluronic acid gel in the treatment of solar keratoses. Australas J Dermatol. 1997;38(4):187-9. | Ineligible patient population |
| Mei X, Wang L, Zhang R, Zhong S. Daylight versus conventional photodynamic therapy for the treatment of actinic keratosis: a meta-analysis of randomized controlled trials. Photodiagnosis Photodyn Ther. 2019;25:23-28. | SR for reference checking |
| Neittaanmakiperttu N, Gronroos M, Karppinen TT, Snellman E, Rissanen P. Photodynamic therapy for actinic keratoses: a randomized prospective non-sponsored cost-effectiveness study of daylightmediated treatment compared with light-emitting diode treatment. Acta Derm Venereol. 2016;96(2):241-44. | Ineligible comparator |
| Neittaanmaki-Perttu N, Gronroos M, Tani T, Snellman E. Long-term outcome of daylight photodynamic therapy with amino-5-laevulinate nanoemulsion vs. methyl-5-aminolaevulinate for actinic keratoses. Acta Derm Venereol. 2016;96(5):712-3. | Ineligible study design |
| Neittaanmaki-Perttu N, Karppinen TT, Tani T, Snellman E, Gronroos M. Long-term outcome of low-concentration hexyl-5-aminolaevulinate daylight photodynamic therapy for treatment of actinic keratoses. Acta Derm Venereol. 2017;97(1):120-21. | Ineligible study design |
| Nistico S, Torchia V, Gliozzi M, Bottoni U, Del Duca E, Muscoli C. Pharmacoeconomy of drugs used in the treatment of actinic keratoses. Int. 2016;29(4):796-804. | Ineligible study design |
| Peris K, Stockfleth E, Gupta G, Aractingi S, Dakovic R, Dirschka T, et al. Efficacy of imiquimod 3.75% from Lmax according to the number of actinic keratosis lesions. J Eur Acad Dermatol Venereol. 2015;29(12):2470-3. | Ineligible subgroup analysis |
| Pomerantz H, Hogan D, Eilers D, Swetter S, Chen SC, Jacob S, et al. Long-term efficacy of topical 5-fluorouracil 5% cream in treating actinic keratosis. J Invest Dermatol. 2015;135(S1):S50. | Ineligible outcomes |
| Pomerantz H, Hogan D, Eilers D, Swetter SM, Chen SC, Jacob SE, et al. Long-term efficacy of topical fluorouracil cream, 5%, for treating actinic keratosis: a randomized clinical trial. JAMA Dermatol. 2015;151(9):952-60. | Ineligible patient population |
| Prince GT, Cameron MC, Fathi R, Alkousakis T. Topical 5-fluorouracil in dermatologic disease. Int J Dermatol. 2018;57(10):1259-64. | SR for reference checking |
| Rivers JK, Arlette J, Shear N, Guenther L, Carey W, Poulin Y. Topical treatment of actinic keratoses with 3.0% diclofenac in 2.5% hyaluronan gel. Br J Dermatol. 2002;146(1):94-100. | Ineligible patient population |

| Reference | Exclusion Reason |
|---|---|
| Rodriguez TM. Don't have to put on the red light: a comparison in outcomes of daylight PDL versus conventional PDL in the treatment of actinic keratosis. J Am Geriatr Soc. 2018;66(S2):S316-S17. | Ineligible study design |
| Sachar M, Siegel JA, Walker JL, Chen SC, Swetter SM, Dellavalle R, et al. 5-fluorouracil decreases rates of persistent actinic keratoses. J Invest Dermatol. 2016;136(5 S1):S45. | Ineligible patient population |
| Samorano LP, Torezan LA, Sanches JA. Evaluation of the tolerability and safety of 0.015% ingenol mebutate gel compared to 5% 5-fluorouracil cream for the treatment of facial actinic keratosis: a prospective randomized trial. J Am Acad Dermatol. 2015;72(5 S1):AB184. | Duplicate |
| Schoppmeyer M. Actinic keratosis: In a comparison of therapies, fluorouracil cream comes off best. Krankenhauspharmazie. 2019;40(8):408-09. | Unobtainable |
| Segatto MM, Dornelles SI, Silveira VB, Frantz Gde O. Comparative study of actinic keratosis treatment with 3% diclofenac sodium and 5% 5-fluorouracil. An Bras Dermatol. 2013;88(5):732-8. | Ineligible patient population |
| Serra-Guillen C, Nagore E, Hueso L, Llombart B, Requena C, Sanmartin O, et al. A randomized comparative study of tolerance and satisfaction in the treatment of actinic keratosis of the face and scalp between 5% imiquimod cream and photodynamic therapy with methyl aminolaevulinate. Br J Dermatol. 2011;164(2):429-33. | Ineligible outcomes |
| Shanghai Skin Disease Hospital. A Randomized single-blind clinical study: the efficacy and safety of Daylight PDT and Red light PDT in the treatment of actinic keratosis. Identifier: ChiCTR-INR-17013506 In: Chinese Clinical Trial Register [internet]. Chengdu: Chinese University of Hong Kong: 2017. Available from http://www.who.int/trialsearch/Trial2.aspx?TrialID=ChiCTR-INR-17013506. | Ineligible comparator |
| Siegel JA, Chren M, Weinstock MA. New keratinocyte carcinomas worsen skin-related quality of life. J Invest Dermatol. 2016;136(5 S1):S31. | Ineligible patient population |
| Siegel JA, Korgavkar K, Weinstock MA. Sunscreen use increases with free provision and regular reminders. J Invest Dermatol. 2016;136(8):B4. | Ineligible patient population |
| Smith S, Piacquadio D, Morhenn V, Atkin D, Fitzpatrick R. Short incubation PDT versus 5-FU in treating actinic keratoses. J Drugs Dermatol. 2003;2(6):629-35. | Ineligible intervention |
| Steeb T, Heppt MV, Becker L, Kohl C, French LE, Berking C. Long-term efficacy of interventions for actinic keratosis: protocol for a systematic review and network meta-analysis. Syst Rev. 2019;8(1):237. | Ineligible publication type |
| Steeb T, Schlager JG, Kohl C, Ruzicka T, Heppt MV, Berking C. Laser-assisted photodynamic therapy for actinic keratosis: a systematic review and meta-analysis. J Am Acad Dermatol. 2019;80(4):947-56. | Ineligible intervention |
| Steinmassl H. Advantage for 5-fluorouracil in actinic keratosis: In direct comparison, other therapies come off worse. Dtsch Apoth Ztg. 2019;159(13) | Ineligible publication type |
| Stockfleth E, Christophers E, Benninghoff B, Sterry W. Low incidence of new actinic keratoses after topical 5% imiquimod cream treatment: a long-term follow-up study. Arch Dermatol. 2004;140(12):1542. | Unobtainable |
| Stockfleth E, Gupta G, Peris K, Aractingi S, Dakovic R, Alomar A. Reduction in lesions from Lmax: a new concept for assessing efficacy of field-directed therapy for actinic keratosis. Results with imiquimod 3.75%. Eur J Dermatol. 2014;24(1):23-7. | Ineligible outcomes |
| Stockfleth E, Harwood CA, Serra-Guillen C, Larsson T, Osterdal ML, Skov T. Response to 'Phase IV head-to-head randomized controlled trial comparing ingenol mebutate 0.015% gel with diclofenac sodium 3% gel for the treatment of | Ineligible publication type |

| Reference | Exclusion Reason |
| --- | --- |
| actinic keratosis on the face or scalp': reply from the authors. Br J Dermatol. 2018;178(3):813-14. | |
| Stockfleth E, Meyer T, Benninghoff B, Salasche S, Papadopoulos L, Ulrich C, et al. A randomized, double-blind, vehicle-controlled study to assess 5% imiquimod cream for the treatment of multiple actinic keratoses. Arch Dermatol. 2002;138(11):1498-502. | Ineligible patient population |
| Stockfleth E, Sibbring GC, Alarcon I. New topical treatment options for actinic keratosis: a systematic review. Acta Derm Venereol. 2016;96(1):17-22. | SR for reference checking |
| Stockfleth E, Ulrich M, Kerl H, Willers C. Long-term sustained efficacy of low dose 5-fluorouracil combined with 10% salicylic acid as a lesion directed treatment for actinic keratoses. Melanoma Res. 2011;21(S1):e53. | Ineligible outcomes |
| Swanson N, Smith CC, Kaur M, Goldenberg G. Imiquimod 2.5% and 3.75% for the treatment of actinic keratoses: two phase 3 multicenter, randomized, double-blind, placebo-controlled studies. J Drugs Dermatol. 2013;12(11):1278-82. | Ineligible patient population |
| Szeimies RM, Karrer S, Radakovic-Fijan S, Tanew A, Calzavara-Pinton PG, Zane C, et al. Photodynamic therapy using topical methyl 5-aminolevulinate compared with cryotherapy for actinic keratosis: a prospective, randomized study. J Am Acad Dermatol. 2002;47(2):258-62. | Ineligible patient population |
| Szeimies RM, Matheson RT, Davis SA, Bhatia AC, Frambach Y, Klovekorn W, et al. Topical methyl aminolevulinate photodynamic therapy using red light-emitting diode light for multiple actinic keratoses: a randomized study. Dermatol Surg. 2009;35(4):586-92. | Ineligible patient population |
| Szeimies RM, Reinhold U, Dirschka T, Hauschild A, Novak B, Schmitz B, et al. Photodynamic therapy for actinic keratosis and basal cell carcinoma using BF-200 ALA: results of pivotal phase III trials and follow-up. J Eur Acad Dermatol Venereol. 2017;31(S3):89. | Ineligible patient population |
| Szeimies R-M, Reinhold U, Dirschka T, Hauschild A, Novak B, Schmitz B, et al. Photodynamic therapy for actinic keratosis and basal cell carcinoma using BF-200 ALA: results of pivotal phase III trials and follow-up. Journal of the european academy of dermatology and venereology : JEADV. 2017; 31: 89-. Available from: https://www.cochranelibrary.com/central/doi/10.1002/central/CN-01400060/full | Does not report comparative results |
| Szeimies RM, Stockfleth E, Popp G, Borrosch F, Bruning H, Dominicus R, et al. Long-term follow-up of photodynamic therapy with a self-adhesive 5-aminolaevulinic acid patch: 12 months data. Br J Dermatol. 2010;162(2):410-4. | Ineligible study design |
| Szeimies RM. A network meta-analysis of the relative efficacy of treatments for actinic keratosis of the face or scalp in Europe. J Eur Acad Dermatol Venereol. 2016;30(9):1619-20. | Ineligible study design |
| Tanghetti E, Tanghetti M. Pre-treatment with topical 5 fluorouracil (5-FU) enhances the efficacy of ALA_PDT for the treatment of actinic keratoses; results of a randomized, controlled clinical trial with post-treatment 5-FU challenge. Lasers Surg Med. 2014;46(S25):46. | Ineligible outcomes |
| Tanghetti EA, Hamann C, Tanghetti M. A controlled comparison study of topical fluourouracil 5% cream pre-treatment of aminolevulinic acid/photodynamic therapy for actinic keratosis. J Drugs Dermatol. 2015;14(11):1241-4. | Ineligible outcomes |
| Tanghetti EA. Comparison of 5-fluorouracil 5% and imiquimod 5% for actinic keratoses. Cosmetic Dermatology. 2004;17(11 S3):16-20. | Ineligible study design |
| Tomas-Velazquez A, Redondo P. Switching from conventional photodynamic therapy to daylight photodynamic therapy for actinic keratoses: systematic review and meta-analysis. Actas Dermosifiliogr. 2017;108(4):282-92. | SR for reference checking |

| **Reference** | **Exclusion Reason** |
|---|---|
| Universidad de Antioquia. Trial of Methyl Aminolevulinate Plus Aktilite in Facial Photodamage. Identifier: NCT00629317. In: ClinicalTrials.gov [internet]. Bethesda: US National Library of Medicine: 2008. Available from https://clinicaltrials.gov/ct2/show/NCT00629317. | Completed trial (no results) |
| von Felbert V, Hoffmann G, Hoff-Lesch S, Abuzahra F, Renn CN, Braathen LR, et al. Photodynamic therapy of multiple actinic keratoses: reduced pain through use of visible light plus water-filtered infrared a compared with light from light-emitting diodes. Br J Dermatol. 2010;163(3):607-15. | Ineligible comparator |
| Walker JL, Siegel JA, Sachar M, Pomerantz H, Chen SC, Swetter SM, et al. 5-fluorouracil for actinic keratosis treatment and chemoprevention: a randomized controlled trial. J Invest Dermatol. 2017;137(6):1367-70. | Ineligible patient population |
| Walker JL, Siegel JA, Sachar M, Qureshi AA, Chen SC, Swetter SM, et al. Single course of 5-fluorouracil treatment prevents new actinic keratoses for 6 to 12 months. J Invest Dermatol. 2016;136(5 S1):S45. | Ineligible patient population |
| Wolf JE, Jr., Taylor JR, Tschen E, Kang S. Topical 3.0% diclofenac in 2.5% hyaluronan gel in the treatment of actinic keratoses. Int J Dermatol. 2001;40(11):709-13. | Ineligible patient population |
| Wu Y, Tang N, Cai L, Li Q. Relative efficacy of 5-fluorouracil compared with other treatments among patients with actinic keratosis: a network meta-analysis. Dermatol Ther. 2019;32(3):e12822. | SR for reference checking |
| Zhao W, Guan M, Nong X, Li Q, Chen Z. The safety and efficacy of daylight photodynamic therapy in the treatment of actinic keratoses: a systematic review and meta-analysis. Int J Dermatol. 2019;58(2):159-66. | SR for reference checking |
| Zhu L, Wang P, Zhang G, Zhang L, Liu X, Hu C, et al. Conventional versus daylight photodynamic therapy for actinic keratosis: a randomized and prospective study in China. Photodiagnosis Photodyn Ther. 2018;24:366-71. | Ineligible comparator |

## Supplementary Table S3: List of the 46 trials included in the review (reported in 86 documents)

Note that blue indicates a paper appearing twice in the table as it reports separate results (i.e. not pooled) for two different studies. This table also includes abstracts that are related to one of the included studies but do not contribute any additional data. These abstracts were not eligible for data extraction.

| **Study Identifier** *(bold text indicates identifier used in this review)* | **Record** |
|---|---|
| **Alomar 2007** | Alomar A, Bichel J, McRae S. Vehicle-controlled, randomized, double-blind study to assess safety and efficacy of imiquimod 5% cream applied once daily 3 days per week in one or two courses of treatment of actinic keratoses on the head. Br J Dermatol. 2007;157(1):133-41. |
| **Athenex 2019a (NCT03285477; KX01-AK-003)** | Athenex. Clinical Study Report: [KX01-AK-003] A Phase 3, Double-Blind, Vehicle-Controlled, Randomized, Parallel Group, Multicenter, Efficacy and Safety Study of KX2-391 Ointment 1% in Adult Subjects with Actinic Keratosis on the Face or Scalp. New Jersey, USA: Athenex Inc.; 09 August 2019a. 1-619. |
| | Athenex I. A Multi-Center Study to Evaluate the Efficacy and Safety of KX2-391 Ointment 1% on AK on Face or Scalp. Identifier: NCT03285477. In: ClinicalTrials.gov [internet]. Bethesda: 2017. Available from https://ClinicalTrials.gov/show/NCT03285477. |
| **Athenex 2019b (NCT03285490; KX01-AK-004)** | Athenex. Clinical Study Report: [KX01-AK-004] A Phase 3, Double-Blind, Vehicle-Controlled, Randomized, Parallel Group, Multicenter, Efficacy and Safety Study of KX2-391 Ointment 1% in Adult Subjects with Actinic Keratosis on the Face or Scalp. New Jersey, USA: Athenex Inc.; 09 September 2019b. 1-620. |
| | Athenex I. A Multi-Center Study to Evaluate the Efficacy and Safety of KX2-391 Ointment 1% on AK on Face or Scalp (AK004). Identifier: NCT03285490. In: ClinicalTrials.gov [internet]. Bethesda: US National Library of Medicine: 2017. Available from https://ClinicalTrials.gov/show/NCT03285490. |
| **Arisi 2020** | Arisi M, Zane C, Polonioli M, Tomasi C, Moggio E, Cozzi C, et al. Effects of MAL-PDT, ingenol mebutate and diclofenac plus hyaluronate gel monitored by high-frequency ultrasound and digital dermoscopy in actinic keratosis- a randomized trial. J Eur Acad Dermatol Venereol. 2020;34(6):1225-32. |
| **Berman 2014** | Berman B, Nestor MS, Newburger J, Park H, Swenson N. Treatment of facial actinic keratoses with aminolevulinic acid photodynamic therapy (ALA-PDT) or ingenol mebutate 0.015% gel with and without prior treatment with ALA-PDT. J Drugs Dermatol. 2014;13(11):1353-6. |
| **Chen 2003** | Chen K, Yap LM, Marks R, Shumack S. Short-course therapy with imiquimod 5% cream for solar keratoses: a randomized controlled trial. Australas J Dermatol. 2003;44(4):250-5 |

| Study Identifier<br>*(bold text indicates identifier used in this review)* | Record |
|---|---|
| **Dirschka 2012**<br><br>NCT02799069<br>EUCTR- 2007-006854-24<br>ALA-AK-CT002 | Biofrontera Bioscience GmbH.  Evaluation of safety and efficacy of BF-200 ALA for the treatment of actinic keratosis with photodynamic therapy.  In: ClinicalTrials.gov [internet].  Bethesda.  US National Library of Medicine.  2008.  Available from https://ClinicalTrials.gov/show/NCT02799069.  Identifier:  NCT02799069 |
| | Biofrontera Bioscience GmbH.  A randomized, observer blind, multinational phase III study to evaluate the safety and efficacy of a nanoemulsion gel formulation BF-200 ALA, in comparison with Metvix® and placebo, for the treatment of actinic keratosis with photodynamic therapy.  In: EU Clinical Trials Register [internet].  London.  European Medicines Agency.  2009.  Available from https://www.clinicaltrialsregister.eu/ctr-search/trial/2017-000486-72/results.  Identifier:  EUCTR2007-006854-24-DE |
| | Dirschka T, Radny P, Dominicus R, Mensing H, Bruning H, Jenne L, et al. Long-term (6 and 12 months) follow-up of two prospective, randomized, controlled phase III trials of photodynamic therapy with BF-200 ALA and methyl aminolaevulinate for the treatment of actinic keratosis.  Br J Dermatol.  2013;168(4):825-36. |
| | Dirschka T, Radny P, Dominicus R, Mensing H, Bruning H, Jenne L, et al. Photodynamic therapy with BF-200 ALA for the treatment of actinic keratosis: results of a multicentre, randomized, observer-blind phase III study in comparison with a registered methyl-5-aminolaevulinate cream and placebo.  Br J Dermatol.  2012;166(1):137-46. |
| | Dirschka T, Radny P, Dominicus R, Mensing H, Bruning H, Jenne L, et al. Efficacy and safety comparison of photodynamic therapy with BF-200 ALA versus Metvix for the treatment of actinic keratosis: a prospective, randomized, placebo-controlled multinational, phase III study.  J Dtsch Dermatol Ges.  2011;9(S1):196-97.  (Abstract; adds no additional information to study) |
| **Dohil 2016: Study 2** | Dohil MA.  Efficacy, safety, and tolerability of 4% 5-fluorouracil cream in a novel patented aqueous cream containing peanut oil once daily compared with 5% 5-fluorouracil cream twice daily: meeting the challenge in the treatment of actinic keratosis.  J Drugs Dermatol.  2016;15(10):1218-24. |
| | Swedish Medical Products Agency. Tolak 40 mg/g kräm: Produktresumé. Uppsala: Swedish Medical Products Agency; 2020. [cited 4 April 2021]. Available from: https://docetp.mpa.se/LMF/Tolak%20cream%20ENG%20SmPC_09001bee809dbaa1.pdf. |
| **Dermapharm AG, 2011: EUCTR- 2011-003317-41**<br><br>11-02 / Diclo-G | Dermapharm AG.  Double-blind, randomized, clinical trial to compare the efficacy and safety of diclofenc 3% gel vs.  solaraze 3% gel vs.  vehicle for the treatment of patients with actinic keratosis.  In: EU Clinical Trials Register [internet].  London.  European Medicines Agency.  2011.  Available from https://www.clinicaltrialsregister.eu/ctr-search/trial/2011-003317-41/DE/.  Identifier:  EUCTR2011-003317-41-DE/ |
| **Dermapharm AG, 2014: EUCTR- 2014-001621-33**<br><br>14-01 / AK-Diclo | Dermapharm AG.  Multicenter, randomised, double-blind clinical trial on the efficacy and safety of medicinal products containing diclofenac in patients with actinic keratosis.  In: EU Clinical Trials Register [internet].  London.  European Medicines Agency.  2014.  Available from https://www.clinicaltrialsregister.eu/ctr-search/trial/2014-001621-33/DE/.  Identifier:  EUCTR2014-001621-33-DE |
| **Biofrontera Bioscience GmbH, 2006: EUCTR-2006-000314-20**<br><br>ALA-AK-CT001 | Biofrontera Bioscience GmbH.  A randomized placebo-controlled clinical trial of topical photodynamic therapy with a nanoemulsion formulation of 5-aminolevulinic acid for the treatment of actinic keratosis.  In: EU Clinical Trials Register [internet].  London.  European Medicines Agency.  2006.  Available from https://www.clinicaltrialsregister.eu/ctr-search/trial/2006-000314-20/DE/.  Identifier:  EUCTR2006-000314-20-DE |

| **Study Identifier** *(bold text indicates identifier used in this review)* | **Record** |
|---|---|
| **Almirall Hermal GmbH, 2007: EUCTR-2007-003889-18**<br><br>H 1005 6002-0702 | Almirall Hermal GmbH. Study on the efficacy of Verrumal(R) compared to placebo and Solaraze(R) in the treatment of actinic keratosis grade I to II. In: EU Clinical Trials Register [internet]. London. European Medicines Agency. 2007. Available from https://www.clinicaltrialsregister.eu/ctr-search/trial/2007-003889-18/DE/. Identifier: EUCTR2007-003889-18-DE |
| **NCT01453179; MEDA Pharma GmbH & Co. KG, 2010: EUCTR-2010-022054-16**<br><br>LEIDA 2 (X-03016-3284) | MEDA Pharma GmbH & Co. KG. Long-term Effects of Imiquimod and Diclofenac in Actinic Keratoses (LEIDA 2). In: ClinicalTrials.gov [internet]. Bethesda. US National Library of Medicine. 2011. Available from https://ClinicalTrials.gov/show/NCT01453179. Identifier: NCT01453179 |
| | MEDA Pharma GmbH & Co. KG. Long-term effects of Aldara® 5% cream and Solaraze® 3% gel in the treatment of actinic keratoses on the face or scalp with respect to the risk of progression to in-situ and invasive squamous cell carcinoma (LEIDA 2). In: EU Clinical Trials Register [internet]. London. European Medicines Agency. 2010. Available from https://www.clinicaltrialsregister.eu/ctr-search/trial/2010-022054-16/DE/. Identifier: EUCTR2010-022054-16-DE |
| **Almirall S.A., 2010: EUCTR-2010-022244-20**<br><br>H 569 000 – 1004 | Almirall S.A. Double-blind, randomized, vehicle- and comparator-controlled, multicenter trial to evaluate the efficacy and safety of LAS41007 in the treatment of actinic keratosis. In: EU Clinical Trials Register [internet]. London. European Medicines Agency. 2010. Available from https://www.clinicaltrialsregister.eu/ctr-search/trial/2010-022244-20/DE/. Identifier: EUCTR2010-022244-20-DE |
| **Foley 2011** | Foley P, Merlin K, Cumming S, Campbell J, Crouch R, Harrison S, et al. A comparison of cryotherapy and imiquimod for treatment of actinic keratoses: lesion clearance, safety, and skin quality outcomes. J Drugs Dermatol. 2011;10(12):1432-8. |
| **Freeman 2003** | Freeman M, Vinciullo C, Francis D, Spelman L, Nguyen R, Fergin P, et al. A comparison of photodynamic therapy using topical methyl aminolevulinate (Metvix) with single cycle cryotherapy in patients with actinic keratosis: a prospective, randomized study. J Dermatolog Treat. 2003;14(2):99-106. |
| | Foley P. A comparison of photodynamic therapy using methyl aminolaevulinate with cryotherapy in actinic keratosis. British Association of Dermatologists 83rd Annual Meeting. Abstract P-67. Br J Dermatol. 2003; 149(S64): P67. Available from: https://onlinelibrary.wiley.com/doi/epdf/10.1046/j.1365-2133.149.s64.8. (Abstract; adds no additional information to study) |
| | Foley P, Freeman M, Vinciullo C, Spelman L, Murrell D, Weightman W, et al. Photodynamic therapy using methyl aminolaevulinate with cryotherapy in actinic keratosis: an Australian study. abstract P-31 The 85th BAD Annual Meeting 5-8th July 2005, Glasgow, UK. Br J Dermatol. 2005; 153(S1): 29. Available from: https://www.cochranelibrary.com/central/doi/10.1002/central/CN-00527201/full. (Abstract; adds no additional information to study) |
| **Hanke 2010**<br><br>NCT00603798 | Graceway Pharmaceuticals. Safety and effectiveness study of imiquimod creams for the treatment of actinic keratoses (AKs). In: ClinicalTrials.gov [internet]. Bethesda. US National Library of Medicine. 2008. Available from https://ClinicalTrials.gov/show/NCT00603798. Identifier: NCT00603798 |

| Study Identifier<br>*(bold text indicates identifier used in this review)* | Record |
|---|---|
| GW01-0703 / 0705 | Hanke CW, Beer KR, Stockfleth E, Wu J, Rosen T, Levy S. Imiquimod 2.5% and 3.75% for the treatment of actinic keratoses: results of two placebo-controlled studies of daily application to the face and balding scalp for two 3-week cycles. J Am Acad Dermatol. 2010;62(4):573-81. |
| **Hauschild 2009 AK03** and **Hauschild 2009 AK04** | Hauschild A, Stockfleth E, Popp G, Borrosch F, Bruning H, Dominicus R, et al. Optimization of photodynamic therapy with a novel self-adhesive 5-aminolaevulinic acid patch: results of two randomized controlled phase III studies. Br J Dermatol. 2009;160(5):1066-74. |
| | Szeimies RM, Stockfleth E, Moor ACE, Stocker M, Ortland C, Hauschild A. A new 5-ALA-patch for the photodynamic therapy of actinic keratoses. Photodiagn Photodyn Ther. 2011;8(2):200. (Abstract; adds no additional information to study) |
| **Jansen 2019**<br><br>NCT02281682<br>EUCTR- 2014-003691-23<br>NL50621.068.14 | Maastricht University Medical Center. IM Versus 5-FU Versus IMI Versus MAL-PDT in Treatment of Actinic Keratosis. In: ClinicalTrials.gov [internet]. Bethesda. US National Library of Medicine. 2014. Available from https://ClinicalTrials.gov/show/NCT02281682. Identifier: NCT02281682 |
| | Jansen MHE, Kessels J, Nelemans PJ, Kouloubis N, Arits A, van Pelt HPA, et al. Randomized trial of four treatment approaches for actinic keratosis. N Engl J Med. 2019;380(10):935-46 |
| | Jansen MHE. Topical treatment of actinic keratosis. Nederlands Tijdschrift voor Dermatologie en Venereologie. 2019;29(3):68. (Abstract; adds no additional information to study) |
| | Jansen MHE, Kessels JPHM, Nelemans PJ, Essers BA, Kelleners-Smeets NWJ, Mosterd K. Ingenolmebutaat bij actinische keratosen. Nederlands Tijdschrift voor Dermatologie en Venereologie. 2018;28(3):24. (Abstract; adds no additional information to study) |
| **Jorizzo 2007** | Jorizzo J, Dinehart S, Matheson R, Moore JK, Ling M, Fox TL, et al. Vehicle-controlled, double-blind, randomized study of imiquimod 5% cream applied 3 days per week in one or two courses of treatment for actinic keratoses on the head. J Am Acad Dermatol. 2007;57(2):265-8. |
| **Korman 2005** | Korman N, Moy R, Ling M, Matheson R, Smith S, McKane S, et al. Dosing with 5% imiquimod cream 3 times per week for the treatment of actinic keratosis: results of two phase 3, randomized, double-blind, parallel-group, vehicle-controlled trials. Arch Dermatol. 2005;141(4):467-73. |
| **Kose 2008** | Kose O, Koc E, Erbil AH, Caliskan E, Kurumlu Z. Comparison of the efficacy and tolerability of 3% diclofenac sodium gel and 5% imiquimod cream in the treatment of actinic keratosis. J Dermatolog Treat. 2008;19(3):159-63. |
| **Lebwohl 2004** | Lebwohl M, Dinehart S, Whiting D, Lee PK, Tawfik N, Jorizzo J, et al. Imiquimod 5% cream for the treatment of actinic keratosis: results from two phase III, randomized, double-blind, parallel group, vehicle-controlled trials. J Am Acad Dermatol. 2004;50(5):714-21. |
| **Peplin, 2008: NCT00700063**<br><br>PEP005-015 | Peplin. A multicenter study to evaluate the safety and efficacy of PEP005 topical gel when used to treat actinic keratoses on the head (face or scalp). In: ClinicalTrials.gov [internet]. Bethesda. US National Library of Medicine. 2008. Available from https://ClinicalTrials.gov/show/NCT00700063 |

| **Study Identifier**<br>*(bold text indicates identifier used in this review)* | **Record** |
|---|---|
| **MEDA Pharma GmbH & Co. KG, 2015: NCT00777127**<br><br>EUCTR-2007-004884-24<br>LEIDA (X-03016-3271) | MEDA Pharma GmbH & Co. KG. Long-term Effects of Imiquimod and Diclofenac in Actinic Keratoses. In: ClinicalTrials.gov [internet]. Bethesda. US National Library of Medicine. 2008. Available from https://ClinicalTrials.gov/show/NCT00777127. Identifier: NCT00777127 |
| | MEDA Pharma GmbH & Co. KG. Long-term effects of Aldara® 5% cream and Solaraze® 3% gel in the treatment of actinic keratoses on the face or scalp (LEIDA). In: EU Clinical Trials Register [internet]. London. European Medicines Agency. 2015. Available from https://www.clinicaltrialsregister.eu/ctr-search/trial/2007-004884-24/DE. Identifier: EUCTR2007-004884-24-DE |
| **Taro Pharmaceuticals USA, 2008: NCT00828568**<br><br>MIQ-0403 | Taro Pharmaceuticals USA. Bioequivalence study of two imiquimod cream 5%. In: ClinicalTrials.gov [internet]. Bethesda. US National Library of Medicine. 2008. Available from https://ClinicalTrials.gov/show/NCT00828568. Identifier: NCT00828568 |
| **Peplin, 2009a: NCT00915551**<br><br>PEP005-025 | Peplin. A multi-center study to evaluate the efficacy and safety of pep005 (ingenol mebutate) gel, when used to treat actinic keratoses on the head (face or scalp). In: ClinicalTrials.gov [internet]. Bethesda. US National Library of Medicine. 2009a. Available from https://ClinicalTrials.gov/show/NCT00915551. Identifier: NCT00915551 |
| **Peplin, 2009b: NCT00916006**<br><br>PEP005-016 | Peplin. A multi-center study to evaluate the efficacy and safety of pep005 (ingenol mebutate) gel, when used to treat actinic keratoses on the head (face or scalp). In: ClinicalTrials.gov [internet]. Bethesda. US National Library of Medicine. 2009b. Available from https://ClinicalTrials.gov/show/NCT00916006. Identifier: NCT00916006 |
| **Gage Development Company, 2016: NCT02952898**<br><br>GDC-695-001 | Gage Development Company. Study comparing GDC 695 and diclofenac sodium gel, 3% in subjects with actinic keratoses. In: ClinicalTrials.gov [internet]. Bethesda. US National Library of Medicine. 2016. Available from https://ClinicalTrials.gov/show/NCT02952898. Identifier: NCT02952898 |
| **Actavis Inc, 2016: NCT03200912**<br><br>094-8152-301 | Actavis Inc. An equivalence study of generic ingenol mebutate gel 0.015% and picato gel 0.015% in subjects with actinic keratosis. In: ClinicalTrials.gov [internet]. Bethesda. US National Library of Medicine. 2016. Available from https://ClinicalTrials.gov/show/NCT03200912. Identifier: NCT03200912 |
| **Pariser 2003**<br><br>NCT00306800<br>PC T404 / 05 | Galderma. Metvix PDT Versus Vehicle PDT With Aktilite CL128 Lamp in Patients With Actinic Keratosis on the Face and Scalp. In: ClinicalTrials.gov [internet]. Bethesda. US National Library of Medicine. 2007. Available from https://ClinicalTrials.gov/show/NCT00306800. Identifier: NCT00306800 |
| | Pariser DM, Lowe NJ, Stewart DM, Jarratt MT, Lucky AW, Pariser RJ, et al. Photodynamic therapy with topical methyl aminolevulinate for actinic keratosis: results of a prospective randomized multicenter trial. J Am Acad Dermatol. 2003;48(2):227-32. |
| **Pariser 2008** | Pariser D, Loss R, Jarratt M, Abramovits W, Spencer J, Geronemus R, et al. Topical methyl-aminolevulinate photodynamic therapy using red light-emitting diode light for treatment of multiple actinic keratoses: a randomized, double-blind, placebo-controlled study. J Am Acad Dermatol. 2008;59(4):569-76. |

| Study Identifier *(bold text indicates identifier used in this review)* | Record |
|---|---|
| **Pariser 2016**<br><br>NCT01475955<br>CP0105 | DUSA Pharmaceuticals. Short-incubation levulan photodynamic therapy versus vehicle for face/scalp actinic keratosis (AK). In: ClinicalTrials.gov [internet]. Bethesda. US National Library of Medicine. 2011. Available from https://ClinicalTrials.gov/show/NCT01475955. Identifier: NCT01475955 |
| | Pariser DM, Houlihan A, Ferdon MB, Berg JE, Group P-AI. Randomized vehicle-controlled study of short drug incubation aminolevulinic acid photodynamic therapy for actinic keratoses of the face or scalp. Dermatol Surg. 2016;42(3):296-304. |
| | Pariser D, McConnehey D, Bukhalo M, Matheson R, Guenthner S, Kempers S. A phase II study of photodynamic therapy (PDT) with aminolevulinic acid HCl (ALA) 20% topical solution + blue light vs ALA topical solution vehicle + blue light using spot and broad area application and incubation times of 1, 2 and 3 hours for the treatment. J Am Acad Dermatol. 2013;68(4 S1):AB156 (Abstract; adds no additional information to study) |
| **Piacquadio 2004** | Fowler Jr JF, Zax RH. Aminolevulinic acid hydrochloride with photodynamic therapy: efficacy outcomes and recurrence 4 years after treatment. Cutis. 2002;69(S6):2-7. |
| | Piacquadio DJ, Chen DM, Farber HF, Fowler JF, Jr., Glazer SD, Goodman JJ, et al. Photodynamic therapy with aminolevulinic acid topical solution and visible blue light in the treatment of multiple actinic keratoses of the face and scalp: investigator-blinded, phase 3, multicenter trials. Arch Dermatol. 2004;140(1):41-6. |
| **Reinhold 2016**<br><br>NCT01966120<br>EUCTR- 2013-002510-12<br>ALA-AK-CT007 | Biofrontera Bioscience GmbH. Safety and efficacy study for the field-directed treatment of actinic keratosis (AK) with photodynamic therapy (PDT). In: ClinicalTrials.gov [internet]. Bethesda. US National Library of Medicine. 2013. Available from https://ClinicalTrials.gov/show/NCT01966120. Identifier: NCT01966120 |
| | Biofrontera Bioscience GmbH. A randomized, double-blind, phase III multi-center study to evaluate the safety and efficacy of BF-200 ALA (Ameluz®) versus placebo in the field-directed treatment of mild to moderate actinic keratosis with photodynamic therapy (PDT) when using BF-RhodoLED®. In: EU Clinical Trials Register [internet]. London. European Medicines Agency. 2013. Available from https://www.clinicaltrialsregister.eu/ctr-search/trial/2013-002510-12/DE/. Identifier: EUCTR2013-002510-12-DE |
| | Reinhold U, Dirschka T, Ostendorf R, Aschoff R, Berking C, Philipp-Dormston WG, et al. A randomized, double-blind, phase III, multicentre study to evaluate the safety and efficacy of BF-200 ALA (Ameluz(R) ) vs. placebo in the field-directed treatment of mild-to-moderate actinic keratosis with photodynamic therapy (PDT) when using the BF-RhodoLED(R) lamp. Br J Dermatol. 2016;175(4):696-705. |
| **Samorano 2015**<br><br>NCT02242747<br>11334 | University of Sao Paulo. Safety and Tolerability Study of Ingenol Mebutate Compared to 5-FU to Treat Facial Actinic Keratosis. In: ClinicalTrials.gov [internet]. Bethesda. US National Library of Medicine. 2014. Available from https://ClinicalTrials.gov/show/NCT02242747. Identifier: NCT02242747 |
| | Samorano LP, Torezan LA, Sanches JA. Evaluation of the tolerability and safety of a 0.015% ingenol mebutate gel compared to 5% 5-fluorouracil cream for the treatment of facial actinic keratosis: a prospective randomized trial. J Eur Acad Dermatol Venereol. 2015;29(9):1822-7. |

| **Study Identifier**<br>*(bold text indicates identifier used in this review)* | **Record** |
|---|---|
| **Serra-Guillen 2012** | Serra-Guillen C, Nagore E, Hueso L, Traves V, Messeguer F, Sanmartin O, et al. A randomized pilot comparative study of topical methyl aminolevulinate photodynamic therapy versus imiquimod 5% versus sequential application of both therapies in immunocompetent patients with actinic keratosis: clinical and histologic outcomes. J Am Acad Dermatol. 2012;66(4):e131-7. |
| **Simon 2015**<br><br>NCT01358851<br>EUCTR-2010-022980-37<br>H 1005 6002 1007 | Almirall SA. LAS41005 in Hyperkeratotic Actinic Keratosis. In: ClinicalTrials.gov [internet]. Bethesda. US National Library of Medicine. 2011. Available from https://ClinicalTrials.gov/show/NCT01358851. Identifier: NCT01358851 |
| | Simon JC, Dominicus R, Karl L, Rodriguez R, Willers C, Dirschka T. A prospective randomized exploratory study comparing the efficacy of once-daily topical 0.5% 5-fluorouracil in combination with 10.0% salicylic acid (5-FU/SA) vs. cryosurgery for the treatment of hyperkeratotic actinic keratosis. J Eur Acad Dermatol Venereol. 2015;29(5):881-9. |
| | Almirall Hermal GmbH. A prospective comparator controlled randomized exploratory study on the efficacy of LAS 41005 compared to cryotherapy in subjects with hyperkeratotic actinic keratosis. In: EU Clinical Trials Register [internet]. London. European Medicines Agency. 2010. Available from https://www.clinicaltrialsregister.eu/ctr-search/trial/2010-022980-37/DE/. Identifier: EUCTR2010-022980-37-DE |
| **Stockfleth 2017**<br><br>NCT02289768<br>EUCTR- 2014-001171-31<br>98605101-1401 | Almirall SA. Study To Evaluate The Efficacy And Safety Of Actikerall® Solution In Patients With Grade I-II Actinic Keratoses. In: ClinicalTrials.gov [internet]. Bethesda. US National Library of Medicine. 2014. Available from https://ClinicalTrials.gov/show/NCT02289768. Identifier: NCT02289768 |
| | Ulrich M, Reinhold U, Falques M, Rodriguez Azeredo R, Stockfleth E. Use of reflectance confocal microscopy to evaluate 5-fluorouracil 0.5%/salicylic acid 10% in the field-directed treatment of subclinical lesions of actinic keratosis: subanalysis of a phase III, randomized, double-blind, vehicle-controlled trial. J Eur Acad Dermatol Venereol. 2018;32(3):390-6. |
| | Stockfleth E, von Kiedrowski R, Dominicus R, Ryan J, Ellery A, Falques M, et al. Efficacy and safety of 5-fluorouracil 0.5%/salicylic acid 10% in the field-directed treatment of actinic keratosis: a phase III, randomized, double-blind, vehicle-controlled trial. Dermatol Ther. 2017;7(1):81-96. |
| | Stockfleth E, von Kiedrowski R, Dominicus R, Ryan J, Ellery A, Falques M, et al. Erratum to: efficacy and safety of 5-fluorouracil 0.5%/salicylic acid 10% in the field-directed treatment of actinic keratosis: a phase III, randomized, double-blind, vehicle-controlled trial. Dermatol Ther. 2017;7(2):263. |
| | Stockfleth E, Von Kiedrowski R, Dominicus R, Ryan J, Ellery A, Falques M, et al. Evaluation of 5-fluorouracil 0.5%/salicylic acid 10% in the field-directed treatment of actinic keratosis: a phase III, randomised, vehicle-controlled trial. Melanoma Res. 2016;26(S1):e10. |
| | Almirall Hermal GmbH. Multicentre, randomized, parallel, double-blind, vehicle controlled study to evaluate the efficacy and safety of Actikerall® solution in the field-directed treatment of actinic keratoses grade I to II (field cancerization). In: EU Clinical Trials Register [internet]. London. European Medicines Agency. 2014. Available from https://www.clinicaltrialsregister.eu/ctr-search/trial/2014-001171-31/DE/. Identifier: EUCTR2014-001171-31-DE |

| Study Identifier<br>*(bold text indicates identifier used in this review)* | Record |
|---|---|
| | Stockfleth E, Ryan J, Falques M, Ivanoff N, Azeredo RR, Vilardell D. Predictors of response to 5-fluorouracil 0.5%/salicylic acid 10% in the field-directed treatment of actinic keratosis: a post hoc analysis of a phase III, randomized, vehicle-controlled trial. J Am Acad Dermatol. 2017;76(6 S1):AB202. (Abstract; adds no additional information to study) |
| **Stockfleth 2018**<br><br>NCT02406014<br>EUCTR- 2014-003218-98<br>LP0041-1120 | LEO Pharma. Efficacy and Safety of Ingenol Mebutate Gel 0.015% Compared to Diclofenac Sodium Gel 3% in Subjects With Actinic Keratoses on the Face or Scalp. In: ClinicalTrials.gov [internet]. Bethesda. US National Library of Medicine. 2015. Available from https://ClinicalTrials.gov/show/NCT02406014. Identifier: NCT02406014 |
| | Stockfleth E, Harwood CA, Serra-Guillen C, Larsson T, Osterdal ML, Skov T. Phase IV head-to-head randomized controlled trial comparing ingenol mebutate 0.015% gel with diclofenac sodium 3% gel for the treatment of actinic keratosis on the face or scalp. Br J Dermatol. 2018;178(2):433-42. |
| | Stockfleth E, Harwood C, Serra-Guillen C, Larsson T, Osterdal ML, Skov T. Safety of ingenol mebutate compared to diclofenac for patients with actinic keratosis on the face or scalp. J Eur Acad Dermatol Venereol. 2017;31(S3):44. |
| | Stockfleth E, Harwood C, Serra-Guillen C, Larsson T, Osterdal ML, Skov T. Efficacy of ingenol mebutate compared to diclofenac for patients with actinic keratosis on the face or scalp. J Eur Acad Dermatol Venereol. 2017;31(S3):43-44. |
| | LEO Pharma. Efficacy and safety of ingenol mebutate gel 0.015% compared to diclofenac sodium gel 3% in subjects with actinic keratoses on the face or scalp In: EU Clinical Trials Register [internet]. London. European Medicines Agency. 2016. Available from https://www.clinicaltrialsregister.eu/ctr-search/trial/2014-003218-98/results. Identifier: EUCTR2014-003218-98-DE |
| **Swanson 2010**<br><br>NCT00605176<br>GW01-0702 / 0704 | Graceway Pharmaceuticals. Safety and effectiveness study of imiquimod creams for treatment of actinic keratoses (AKs). In: ClinicalTrials.gov [internet]. Bethesda. US National Library of Medicine. 2008. Available from https://ClinicalTrials.gov/show/NCT00605176. Identifier: NCT00605176 |
| | Swanson N, Abramovits W, Berman B, Kulp J, Rigel DS, Levy S. Imiquimod 2.5% and 3.75% for the treatment of actinic keratoses: results of two placebo-controlled studies of daily application to the face and balding scalp for two 2-week cycles. J Am Acad Dermatol. 2010;62(4):582-90. |
| | Swanson N, Smith CC, Kaur M, Goldenberg G. Imiquimod 2.5% and 3.75% for the treatment of actinic keratoses: two phase 3, multicenter, randomized, double-blind, placebo-controlled studies. J Drugs Dermatol. 2014;13(2):166-9. |
| | Swanson N, Jorizzo J, Kaur M. Imiquimod 2.5% and 3.75% for the treatment of actinic keratosis: assessment of efficacy in 479 randomized patients. J Am Acad Dermatol. 2014;70(5 S1):AB127. |
| **Szeimies 2004** | Szeimies RM, Gerritsen MJ, Gupta G, Ortonne JP, Serresi S, Bichel J, et al. Imiquimod 5% cream for the treatment of actinic keratosis: results from a phase III, randomized, double-blind, vehicle-controlled, clinical trial with histology. J Am Acad Dermatol. 2004;51(4):547-55. |
| **Szeimies 2010**<br><br>NCT02799082 | Biofrontera Bioscience GmbH. Evaluation of efficacy and safety of BF-200 ala used with photodynamic therapy in patients with actinic keratosis. In: ClinicalTrials.gov [internet]. Bethesda. US National Library of Medicine. 2007. Available from https://ClinicalTrials.gov/show/NCT02799082. Identifier: NCT02799082 |

| **Study Identifier** *(bold text indicates identifier used in this review)* | **Record** |
|---|---|
| EUCTR- 2007-003371-39<br>ALA-AK-CT003 | Dirschka T, Radny P, Dominicus R, Mensing H, Bruning H, Jenne L, et al. Long-term (6 and 12 months) follow-up of two prospective, randomized, controlled phase III trials of photodynamic therapy with BF-200 ALA and methyl aminolaevulinate for the treatment of actinic keratosis. Br J Dermatol. 2013;168(4):825-36. |
| | Szeimies RM, Radny P, Sebastian M, Borrosch F, Dirschka T, Krahn-Senftleben G, et al. Photodynamic therapy with BF-200 ALA for the treatment of actinic keratosis: results of a prospective, randomized, double-blind, placebo-controlled phase III study. Br J Dermatol. 2010;163(2):386-94. |
| | Biofrontera Bioscience GmbH. A randomized, double-blind, phase III multicenter study evaluating the safety and efficacy of BF-200 ALA versus placebo in the treatment of actinic keratosis when using PDT. In: EU Clinical Trials Register [internet]. London. European Medicines Agency. 2007. Available from https://www.clinicaltrialsregister.eu/ctr-search/trial/2007-003371-39/DE/. Identifier: EUCTR2007-003371-39-DE |
| | Szeimies RM, Borrosch F, Dirschka T, KrahnSenftleben G, Radny P, Reich K, et al. Two prospective, randomized, placebo-controlled, double-blinded clinical studies with BF-200 ALA for photodynamic therapy of actinic keratosis. J Dtsch Dermatol Ges. 2011;9(S1):197. (Abstract; adds no additional information to study) |
| **Tanghetti 2007** | Tanghetti E, Werschler P. Comparison of 5% 5-fluorouracil cream and 5% imiquimod cream in the management of actinic keratoses on the face and scalp. J Drugs Dermatol. 2007;6(2):144-7. |
| **Zane 2014** | Zane C, Facchinetti E, Rossi MT, Specchia C, Calzavara-Pinton PG. A randomized clinical trial of photodynamic therapy with methyl aminolaevulinate vs. diclofenac 3% plus hyaluronic acid gel for the treatment of multiple actinic keratoses of the face and scalp. Br J Dermatol. 2014;170(5):1143-50. |

## Supplementary Table S4: 6 papers listed for information (not data extracted)

| Record | Study identifiers | Notes |
|---|---|---|
| Almirall. Post-hoc statistical analyses of studies KX01-AK-003 and KX01-AK-004 to support the dossier of Tirbanibulin (KX2-391) ointment 1% in actinic keratosis on face or scalp. Barcelona: Almirall SA; 26 November 2019. 1-84. | NCT03285477 and NCT03285490 | Document reports pooled data from the two Athenex trials |
| Del Rosso J, Swanson N, Berman B, Martin GM, Lin T, Rosen T. Imiquimod 2.5% and 3.75% cream for the treatment of photodamage: a meta-analysis of efficacy and tolerability in 969 randomized patients. J Clin Aesthet Dermatol. 2018;11(9):28-31. | NCT00605176 / GW01-0702 / 0704 (two trials reported together) *and* NCT00603798 / GW01-0703 / 0705 (two trials reported together) | Paper reports pooled outcomes for four trials. We already have full papers reporting these trials in pooled pairs, rather than as a group of four. We will use the data reported for pairs of trials rather than the group of four. |
| Del Rosso J, Swanson N, Berman B, Rosen T. Imiquimod 2.5% and 3.75% for the treatment of photodamage: meta-analysis of efficacy and tolerability in 969 randomized patients. J Clin Aesthet Dermatol. 2017;10(5 S1):S12. *(Abstract; adds no additional information to study)* | | |
| Gollnick H, Dirschka T, Ostendorf R, Kerl H, Kunstfeld R. Long-term clinical outcomes of imiquimod 5% cream vs. diclofenac 3% gel for actinic keratosis on the face or scalp: a pooled analysis of two randomized controlled trials. JEADV. 2019: Available from: [https://www.cochranelibrary.com/central/doi/10.1002/central/CN-02053677/full](https://www.cochranelibrary.com/central/doi/10.1002/central/CN-02053677/full) | MEDA Pharma GmbH & Co. KG, 2015: NCT00777127 / 2007-004884-24 / LEIDA (X-03016-3271) *and* NCT01453179 / 2010-022054-16 / LEIDA 2 (X-03016-3284) | Paper reports pooled outcomes for two trials, both of which we have full papers and NCT records for. We will use the disaggregated data from the above sources rather than extracting the pooled data. |
| Lebwohl M, Swanson N, Anderson LL, Melgaard A, Xu Z, Berman B. Ingenol mebutate gel for actinic keratosis. N Engl J Med. 2012;366(11):1010-9. | NCT00915551 / PEP005-025 *and* NCT00916006 / PEP005-016 | Paper reports pooled outcomes for two trials, both of which we have full papers and NCT records for. We will use the disaggregated data from the above sources rather than extracting the pooled data. |
| Morales Toquero A, Ocampo Candiani J, Gomez Flores M, Gonzalez Gonzalez SE, Eguia Rodriguez R, Mendez Olvera NP, et al. Clinical and histological evaluation of 5% imiquimod cream vs 5% 5-fluorouracil ointment in patients with actinic keratosis on the face. Dermatol Rev Mex. 2010;54(6):326-31.. | Paper not in English. Listed (as per protocol) but not eligible for data extraction. | |

## Supplementary Table S5: Details of the 46 trials included in the review

| **Study identifier, duration of trial**, and location** | **Participants randomised** | **Trial design (as described by authors)** | **Primary and secondary outcomes and outcome measures** | **Duration of treatment** | **Patient characteristics**<br><br>**Age** ***(mean (SD) in years)***<br><br>**Gender** ***(N (%) male)***<br><br>**Lesion count per patient at baseline** ***(mean (SD))*** |
|---|---|---|---|---|---|
| Alomar 2007 [61]<br><br>20 weeks<br><br>20 study centres (four in France, six in Germany, three in Italy, two in the Netherlands, three in Spain, and two in the UK) | 259 | Vehicle-controlled, randomised study<br><br>Double-blind | **Primary outcome(s)**<br>Complete clearance<br><br>**Secondary outcome(s)**<br>Partial clearance (75% reduction) and clearance of individual AK lesions (reduction in all AK lesions from all patients totalled together). Safety evaluations included physical examinations, clinical laboratory tests (haematology, blood chemistry and urinalysis), and urine pregnancy tests for women of childbearing potential. AEs and the use of concomitant medications were monitored at each study visit beginning with treatment initiation, as were LSRs | **IMQ5%:** One 4-week course, with the potential for a second course 4 weeks later | Age<br>70.3 (8.45); Range: 44 to 90<br><br>Gender (male)<br>115 (89.1)<br><br>Lesion count NR |
| | | | | **PLAC_TOP:** One 4-week course, with the potential for a second course 4 weeks later | Age<br>71.85 (7.25); Range: 53 to 94<br><br>Gender (male)<br>113 (86.9)<br><br>Lesion count NR |
| Arisi 2020 [84]<br><br>13 weeks<br><br>Dermatology Department of the University of Brescia, Italy | 90 | Randomised comparative noninferiority study<br><br>Single-blind | **Primary outcome(s)**<br>AKs cumulative area<br><br>**Secondary outcome(s)**<br>Number of patients attaining the clearance of all lesions (complete responders) in the 25 $cm^2$ treated area | **MAL_PDT:** One session, with potential for a second session 12 weeks later | Age<br>Median: 80; Range: 71 to 89<br><br>Gender (male)<br>26 (100)<br><br>Lesion count |

| Study identifier, duration of trial**, and location | Participants randomised | Trial design (as described by authors) | Primary and secondary outcomes and outcome measures | Duration of treatment | **Patient characteristics**<br><br>**Age** *(mean (SD) in years)*<br><br>**Gender** *(N (%) male)*<br><br>**Lesion count per patient at baseline** *(mean (SD))* |
|---|---|---|---|---|---|
| | | | | | Median: 9; Range: 5 to 12 |
| | | | | **IM0.015%:** 3 days | Age<br>Median: 76; Range: 49 to 89<br><br>Gender (male)<br>23 (76.7)<br><br>Lesion count<br>Median: 7; Range: 5 to 13 |
| | | | | **DICLO3%:** 90 days | Age<br>Median: 77; Range: 54 to 91<br><br>Gender (male)<br>27 (96.4)<br><br>Lesion count<br>Median: 7; Range: 5 to 12 |
| Athenex, Inc 2019a<br>KX01-AK-003;<br>NCT03285477 [53]<br><br>15 months | 351 | Multicenter, randomised, double-blind, vehicle-controlled, | **Primary outcome(s)**<br>Complete and partial clearance<br><br>**Secondary outcome(s)**<br>• Recurrence | **TIRBA1%:** Once-daily self-administration for 5 consecutive days | Age<br>69.5 (8.5)<br><br>Gender (male)<br>147 (84) |

| Study identifier, duration of trial**, and location | Participants randomised | Trial design (as described by authors) | Primary and secondary outcomes and outcome measures | Duration of treatment | Patient characteristics<br><br>**Age** *(mean (SD) in years)*<br><br>**Gender** *(N (%) male)*<br><br>**Lesion count per patient at baseline** *(mean (SD))* |
|---|---|---|---|---|---|
| 31 study sites in the USA | | parallel-group study | • AEs<br>• Serious AEs (SAEs)<br>• LSRs (erythema, flaking / scaling, crusting, swelling, vesiculation / pustulation, and erosion / ulceration)<br>• Events of special interest (skin cancers, ocular exposure, overdose, and pregnancy)<br>• Pigmentation and scarring<br>• Vital signs, electrocardiograms (ECGs), physical examinations (PEs) including weight, and laboratory evaluation of haematology, biochemistry, and urinalyses at prespecified timepoints. | | Lesion count<br>Median 6; Range 4 to 8 |
| | | | | **PLAC_TOP:** Once-daily self-administration for 5 consecutive days | Age<br>70.2 (9.4)<br><br>Gender (male)<br>154 (88)<br><br>Lesion count<br>Median 6; Range 4 to 8 |
| Athenex, Inc 2019b KX01-AK-004; NCT03285490 [54]<br><br>15 months<br><br>31 study sites in the USA | 351 | Multicenter, randomised, double-blind, vehicle-controlled, parallel-group study | **Primary outcome(s)**<br>Complete and partial clearance<br><br>**Secondary outcome(s)**<br>• Recurrence<br>• AEs<br>• Serious AEs (SAEs)<br>• LSRs (erythema, flaking / scaling, crusting, swelling, vesiculation / pustulation, and erosion / ulceration)<br>• Events of special interest (skin cancers, ocular exposure, overdose, and pregnancy)<br>• Pigmentation and scarring<br>• Vital signs, electrocardiograms (ECGs), physical examinations (PEs) including weight, and laboratory evaluation of haematology, biochemistry, and urinalyses at prespecified timepoints. | **TIRBA1%:** Once-daily self-administration for 5 consecutive days | Age<br>69.1 (8.7)<br><br>Gender (male)<br>158 (89)<br><br>Lesion count<br>Median 6; Range 4 to 8 |
| | | | | **PLAC_TOP:** Once-daily self-administration for 5 consecutive days | Age<br>70.2 (8.9)<br><br>Gender (male)<br>150 (87)<br><br>Lesion count<br>Median 6; Range 4 to 8 |

| **Study identifier, duration of trial**, and location** | **Participants randomised** | **Trial design (as described by authors)** | **Primary and secondary outcomes and outcome measures** | **Duration of treatment** | **Patient characteristics**<br><br>**Age** ***(mean (SD) in years)***<br><br>**Gender** ***(N (%) male)***<br><br>**Lesion count per patient at baseline** ***(mean (SD))*** |
|---|---|---|---|---|---|
| Berman 2014 [93]<br><br>10 weeks<br><br>One centre in the USA | 16 | Randomised, three-group study<br><br>Open-label | **Primary outcome(s)**<br>Lesion clearance; LSRs; AEs<br><br>**Secondary outcome(s)**<br>NR | **ALA_PDT:** Two sessions two weeks apart | Age, gender and lesion count NR |
| | | | | **IM0.015%:** 3 days | Age, gender and lesion count NR |
| Chen 2003 [51]<br><br>14 weeks<br><br>Total number of sites unclear. Skin and Cancer Foundation Victoria, St Vincent's Hospital Melbourne, and private dermatology practices in Victoria and New South Wales, Australia | 44 | A randomised, vehicle-controlled study<br><br>Double-blind | **Primary outcome(s)**<br>The number of subjects achieving a clearance of 75% of baseline AK<br><br>**Secondary outcome(s)**<br>LSRs and AEs | **IMQ5%:** One 3-week course (i.e. 3 weeks' treatment, 4 weeks off) with the potential for a second course at the end of the first | Age<br>64.9 (10.2)<br><br>Gender (male)<br>19 (65.5*)<br><br>Lesion count<br>10.5 (2.8) |
| | | | | **PLAC_TOP:** One 3-week course (i.e. 3 weeks' treatment, 4 weeks off) with the potential for a second course at the end of the first | Age<br>63.0 (12.1)<br><br>Gender (male)<br>4* (40*)<br><br>Lesion count<br>10.8 (3.7) |
| Dirschka 2012 [73]<br><br>12 to 24 weeks | 571 | Multicentre, placebo-controlled, interindividual RCT | **Primary outcome(s)**<br>Complete clearance rate<br><br>**Secondary outcome(s)**<br>Rate of completely cleared lesions | **ALA_PDT:** 1 session, with potential for retreatment at 12 weeks | Age<br>70.2 (7.18)<br><br>Gender (male)<br>214 (86.3) |

<table>
<tr><th>Study identifier, duration of trial**, and location</th><th>Participants randomised</th><th>Trial design (as described by authors)</th><th>Primary and secondary outcomes and outcome measures</th><th>Duration of treatment</th><th>Patient characteristics<br><br>Age (mean (SD) in years)<br><br>Gender (N (%) male)<br><br>Lesion count per patient at baseline (mean (SD))</th></tr>
<tr><td rowspan="3">26 study centres in Germany, Austria and Switzerland</td><td rowspan="3"></td><td rowspan="3">Single-blind</td><td rowspan="3"></td><td></td><td>Lesion count<br>6.1 (1.6)</td></tr>
<tr><td>MAL_PDT: 1 session, with potential for retreatment at 12 weeks</td><td>Age<br>71.0 (6.93)<br><br>Gender (male)<br>205 (83.3)<br><br>Lesion count<br>6.3 (1.5)</td></tr>
<tr><td>PLAC_PDT: 1 session, with potential for retreatment at 12 weeks</td><td>Age<br>71.5 (6.68)<br><br>Gender (male)<br>60 (78.9)<br><br>Lesion count<br>6.4 (1.4)</td></tr>
<tr><td rowspan="4">Dohil 2016: Study 2 [63]<br><br>8 weeks<br><br>27 centres in the USA</td><td rowspan="4">841</td><td rowspan="4">Randomised, parallel-group study<br><br>Double-blind</td><td rowspan="4">Primary outcome(s)<br>100% clearing of AK lesions<br><br>Secondary outcome(s)<br>• The proportion of subjects who had 75% clearing of AK lesions compared with baseline, in the ITT population.</td><td>5FU4%: 4 weeks (once daily)</td><td rowspan="4">Age<br>Overall: Around 68 years<br><br>Gender (male)<br>Overall: Ratio of male to female was about 3:1</td></tr>
<tr><td>5FU5%: 4 weeks (twice daily)</td></tr>
<tr><td>PLAC_TOP: 4 weeks (once daily)</td></tr>
<tr><td>PLAC_TOP: 4 weeks (twice daily)</td></tr>
</table>

| Study identifier, duration of trial**, and location | Participants randomised | Trial design (as described by authors) | Primary and secondary outcomes and outcome measures | Duration of treatment | Patient characteristics<br><br>**Age** *(mean (SD) in years)*<br><br>**Gender** *(N (%) male)*<br><br>**Lesion count per patient at baseline** *(mean (SD))* |
|---|---|---|---|---|---|
| | | | • The percentage change from baseline in number of AK lesions, in the ITT population.<br>• 100% clearing of AK lesions, in the PP population.<br>• 75% clearing of AK lesions compared with baseline, in the PP population.<br>• The percentage change from baseline in number of AK lesions, in the PP population. | | Lesion count<br>Overall: "Average" 15; Range: 5 to 90 |
| Dermapharm AG, 2011: EUCTR- 2011-003317-41 [94]<br><br>17 weeks<br><br>12 centres in Germany | 339 | Randomised trial<br><br>Double-blind | **Primary outcome(s)**<br>Complete clearance<br><br>**Secondary outcome(s)**<br>• Course of the cumulative lesion number score (CLNS) between beginning of treatment (day 0) and final examination or early termination.<br>• Evaluation of the global improvement by the investigator (IGII) and by the patient (PGII) during the whole study course.<br>• Proportion of patients with the IGII rating "cured" at final examination.<br>• Evaluation of the tolerability of the treatment by the investigator and the patient.<br>• Safety (laboratory assessment)<br>• AEs | **DICLO3%:** 90 days (13 weeks)<br><br>**PLAC_TOP:** 90 days (13 weeks) | Age, gender and lesion count NR<br><br>Age, gender and lesion count NR |
| Dermapharm AG, 2014: EUCTR- 2014-001621-33 [70] | 293 | Multicenter, randomised, clinical trial | **Primary outcome(s)**<br>Proportion of patients in the PP population with reduction of the Target Lesion Number Score (TLNS) of ≥75% at the final examination | **DICLO3%:** NR, but may be 13 weeks as previous trial (Dermapharm AG, 2011: EUCTR- 2011-003317-41) | Age<br>Overall: Adults (18-64 years): 66 |

| Study identifier, duration of trial**, and location | Participants randomised | Trial design (as described by authors) | Primary and secondary outcomes and outcome measures | Duration of treatment | Patient characteristics<br><br>Age *(mean (SD) in years)*<br><br>Gender *(N (%) male)*<br><br>Lesion count per patient at baseline *(mean (SD))* |
|---|---|---|---|---|---|
| 17 weeks<br><br>Multiple sites in Germany (number not stated) | | Double-blind | **Secondary outcome(s)**<br>• Proportion of patients with a TLNS reduction of ≥75% at the end of treatment<br>• Changes of the severity of the target lesions<br>• Course of the cumulative lesion number score (CLNS) between beginning of treatment (day 0) and final examination or early termination<br>• Evaluation of the global improvement by the investigator (IGII) and by the patient (PGII) during the whole study course<br>• Proportion of patients with the IGII rating "cured" at final examination<br>• 100% clearance of all AK lesions at the final examination (CLNS =0) and proportion of patients with 100% reduction of the TLNS | with same methods and investigators<br><br>**PLAC_TOP:** NR, but may be 13 weeks as previous trial (Dermapharm AG, 2011: EUCTR- 2011-003317-41) with same methods and investigators | 65 to 84 years: 355<br>85 years and over: 18<br><br>Gender (male)<br>Overall: 371 (84.5*)<br><br>Lesion count NR |
| Biofrontera Bioscience GmbH, 2006: EUCTR-2006-000314-20 [81]<br><br>12 months<br><br>11 study centres in Germany | 55 | Randomised placebo-controlled clinical trial<br><br>Double-blind | **Primary outcome(s)**<br>Complete clearance<br><br>**Secondary outcome(s)**<br>Safety and tolerability, cosmetic outcome, recurrence rates of AK lesions within the treated area | **ALA_PDT:** One session<br><br>**PLAC_PDT:** One session | Age<br>Overall mean age: 71.1<br>Range between the different treatment groups: 69.9 to 72.6<br><br>Gender NR<br><br>Lesion count |

| Study identifier, duration of trial**, and location | Participants randomised | Trial design (as described by authors) | Primary and secondary outcomes and outcome measures | Duration of treatment | **Patient characteristics**<br><br>**Age** ***(mean (SD) in years)***<br><br>**Gender** ***(N (%) male)***<br><br>**Lesion count per patient at baseline** ***(mean (SD))*** |
|---|---|---|---|---|---|
| | | | | | ALA_PDT: Face / forehead: 4.4; Bald scalp: 5.3<br>PLAC_PDT: NR |
| Almirall Hermal GmbH, 2007: EUCTR-2007-003889-18 [79]<br><br>20 weeks<br><br>38 sites in Germany | 470 | Randomised, active- and placebo-controlled trial<br><br>Double-blind | **Primary outcome(s)**<br>Histological status of (one) target lesion (cleared, not cleared)<br><br>**Secondary outcome(s)**<br>Lesion response, tolerability and safety | **5FU0.5%+SA:** 12 weeks<br>**DICLO3%:** 12 weeks<br>**PLAC_TOP:** 12 weeks | Age<br>Overall: 71.8 (between 71.6 and 72.3 between treatment groups) across the FAS<br><br>Gender (male)<br>Overall: 398* (84.7) (between 84.4% and 85.9% between the treatment groups) across the safety set<br><br>Lesion count<br>5FU0.5%+SA: 5.8<br>DICLO3%:5.9<br>PLAC_TOP: 5.6 |
| MEDA Pharma GmbH & Co. KG, 2010: EUCTR-2010-022054-16<br><br>LEIDA 2 | 221 | RCT<br><br>Open-label | **Primary outcome(s)**<br>To determine long-term risk of progression to squamous cell cancer (SCC). Histological finding of an in situ SCC or an invasive SCC after start of treatment will be considered as "histological progression", which is the primary endpoint. | **IMQ5%:** Each cycle could be either: 4 weeks' treatment, 4 weeks off, 4 weeks' treatment, OR 4 weeks' treatment, 8 weeks | Age<br>71.36 (8.1)<br><br>Gender (male)<br>92 (83.6*) |

| Study identifier, duration of trial**, and location | Participants randomised | Trial design (as described by authors) | Primary and secondary outcomes and outcome measures | Duration of treatment | **Patient characteristics**<br><br>**Age** ***(mean (SD) in years)***<br><br>**Gender** ***(N (%) male)***<br><br>**Lesion count per patient at baseline** ***(mean (SD))*** |
|---|---|---|---|---|---|
| [90]<br><br>36 months<br><br>14 sites in Germany and 3 in Austria | | | **Secondary outcome(s)**<br>• Recurrence rates<br>• Time to recurrence<br>• Need of rescue treatment<br>• Cosmetic outcome | off. Patients received between 1 and 6 cycles. | Lesion count NR |
| | | | | **DICLO3%:** 12 weeks. Patients received between 1 and 6 cycles. | Age<br>70.75 (8.4)<br><br>Gender (male)<br>99 (89.2*)<br><br>Lesion count NR |
| Almirall S.A., 2010: EUCTR-2010-022244-20 [71]<br><br>21 weeks (12 months post-treatment follow up was planned but study was discontinued before this)<br><br>40 sites in Germany, 5 in the UK, and 9 in Poland | 508 | Randomised, vehicle- and comparator-controlled, multicenter trial<br><br>Double-blind | **Primary outcome(s)**<br>Histological clearance of one pre-selected AK lesion as a marker of complete clearance<br><br>**Secondary outcome(s)**<br>Rate of clinical clearance (target lesions, or target + non-target lesions + new lesions) and rate of responders, reduction of total AK target lesion area per patient, improvement of target lesions. Safety determined by incidence of AEs, tolerability (overall and local), laboratory variables, vital signs, and the patient's compliance. | **DICLO3%:** 90 days (13 weeks) | Age<br>Overall: 72.7; Range: 39 to 94<br><br>Gender (male)<br>Overall: 791 (89)<br><br>Lesion count<br>DICLO3%: Median: 9<br>PLAC_TOP: Median: 9 |
| | | | | **PLAC_TOP:** 90 days (13 weeks) | |

| Study identifier, duration of trial**, and location | Participants randomised | Trial design (as described by authors) | Primary and secondary outcomes and outcome measures | Duration of treatment | Patient characteristics<br><br>Age *(mean (SD) in years)*<br><br>Gender *(N (%) male)*<br><br>Lesion count per patient at baseline *(mean (SD))* |
|---|---|---|---|---|---|
| Foley 2011 [86]<br><br>12 months<br><br>Single site in Australia (Melbourne) | 71 | Single centre prospective RCT<br><br>Open-label | **Primary outcome(s)**<br>Lesion response rates<br><br>**Secondary outcome(s)**<br>Patient response rates, AEs | **IMQ5%:** One 3 to 4 week course with potential for a second course 4 weeks later | Age<br>71.3 (1.88)<br><br>Gender (male)<br>28 (80)<br><br>Lesion count<br>Total across arm: 340 (35 patients) |
| | | | | **CRYO:** 1 session, with potential for retreatment at 3, 6 and 9 months | Age<br>71.7 (1.63)<br><br>Gender (male)<br>28 (77.8)<br><br>Lesion count<br>Total across arm: 360 (36 patients) |
| Freeman 2003 [77]<br><br>13 weeks<br><br>9 study centres in Australia | 200 | Randomised, reference- and placebo-controlled, parallel group multicentre study | **Primary outcome(s)**<br>Lesion response rate, overall cosmetic outcome, cosmetic outcome in individual lesions, patient satisfaction, AEs<br><br>**Secondary outcome(s)**<br>NR | **MAL_PDT:** Two sessions of PDT one week apart | Age<br>64; Range: 33 to 86<br><br>Gender (male)<br>49 (56)<br><br>Lesion count overall<br>1–2: 48 (55)<br>3–7: 27 (31) |

| Study identifier, duration of trial**, and location | Participants randomised | Trial design (as described by authors) | Primary and secondary outcomes and outcome measures | Duration of treatment | Patient characteristics<br><br>Age *(mean (SD) in years)*<br><br>Gender *(N (%) male)*<br><br>Lesion count per patient at baseline *(mean (SD))* |
|---|---|---|---|---|---|
| | | The study was open with regard to PDT versus cryosurgery and double-blind with regard to methyl aminolevulinate versus placebo PDT | | | 8–28: 13 (15) |
| | | | | **PLAC_PDT:** Two sessions of PDT one week apart | Age<br>66; Range: 49 to 89<br><br>Gender (male)<br>16 (70)<br><br>Lesion count overall<br>1–2: 13 (57)<br>3–7: 8 (35)<br>8–28: 2 (9) |
| | | | | **CRYO:** One session | Age<br>65; Range: 38 to 86<br><br>Gender (male)<br>54 (61)<br><br>Lesion count overall<br>1–2: 39 (44)<br>3–7: 31 (35)<br>8–28: 19 (21) |
| Hanke 2010 [58]<br><br>17 weeks<br><br>26 USA study centres | 326 | Two randomised, multicenter, placebo-controlled studies | **Primary outcome(s)**<br>Complete clearance<br><br>**Secondary outcome(s)**<br>• 75% reduction in the number of AK lesions in the treatment area compared with baseline | **IMQ3.75%:** Two 3-week treatment cycles, i.e. 3 weeks' treatment, 3 weeks off, 3 weeks' treatment | Age<br>64.3 (10.2)<br><br>Gender (male)<br>123 (75.9) |

| Study identifier, duration of trial**, and location | Participants randomised | Trial design (as described by authors) | Primary and secondary outcomes and outcome measures | Duration of treatment | **Patient characteristics**<br><br>**Age** ***(mean (SD) in years)***<br><br>**Gender** ***(N (%) male)***<br><br>**Lesion count per patient at baseline** ***(mean (SD))*** |
|---|---|---|---|---|---|
| | | Double-blind | • Percent change from baseline in AK lesion count<br>• Local skin reactions | | Lesion count<br>11.1 (4.9) |
| | | | | **PLAC_TOP:** Two 3-week treatment cycles, i.e. 3 weeks' treatment, 3 weeks off, 3 weeks' treatment | Age<br>63.7 (10.9)<br><br>Gender (male)<br>135 (82.3)<br><br>Lesion count<br>10.3 (4.3) |
| Hauschild 2009 AK 03 [78]<br><br>12 weeks<br><br>29 centres in Germany | 103 | Two separate confirmatory randomised parallel-group studies<br><br>Single-blind | **Primary outcome(s)**<br>Lesion complete clearance, LSRs, AEs<br><br>**Secondary outcome(s)**<br>NR | **ALA_PDT Patch:** One session | Age<br>70.4 (8.32)<br><br>Gender (male)<br>58 (84)<br><br>Lesion count<br>5.8 (1.91) |
| | | | | **PLAC_PDT Patch:** One session | Age<br>71.4 (6.77)<br><br>Gender (male)<br>26 (76)<br><br>Lesion count<br>5.5 (1.91) |

| Study identifier, duration of trial**, and location | Participants randomised | Trial design (as described by authors) | Primary and secondary outcomes and outcome measures | Duration of treatment | **Patient characteristics**<br><br>**Age** *(mean (SD) in years)*<br><br>**Gender** *(N (%) male)*<br><br>**Lesion count per patient at baseline** *(mean (SD))* |
|---|---|---|---|---|---|
| Hauschild 2009 AK 04 [78]<br><br>12 weeks<br><br>29 centres in Germany | 346 | Two separate confirmatory randomised parallel-group studies<br><br>Open-label | **Primary outcome(s)**<br>Lesion complete clearance, LSRs, AEs<br><br>**Secondary outcome(s)**<br>NR | **ALA_PDT Patch**: One session | Age<br>70.0 (8.32)<br><br>Gender (male)<br>104 (70)<br><br>Lesion count<br>5.8 (1.64) |
| | | | | **PLAC_PDT patch**: One session | Age<br>71.6 (7.54)<br><br>Gender (male)<br>40 (82)<br><br>Lesion count<br>5.9 (1.76) |
| | | | | **CRYO:** One session | Age<br>70.6 (8.73)<br><br>Gender (male)<br>104 (70)<br><br>Lesion count<br>5.4 (1.57) |
| Jansen 2019 [28]<br><br>Up to 72 weeks | 624 | Multicenter, randomised trial | **Primary outcome(s)** | **5FU5%:** One 4-week treatment course, with | Age<br>Median: 74; Range: 48 to 90 |

| Study identifier, duration of trial**, and location | Participants randomised | Trial design (as described by authors) | Primary and secondary outcomes and outcome measures | Duration of treatment | Patient characteristics<br><br>**Age** *(mean (SD) in years)*<br><br>**Gender** *(N (%) male)*<br><br>**Lesion count per patient at baseline** *(mean (SD))* |
|---|---|---|---|---|---|
| Four hospitals (Maastricht University Medical Centre, Catharina Hospital, VieCuri Medical Centre and Zuyderland Medical Centre) in the Netherlands | | Single-blind | Proportion of patients who remained free from treatment failure during 12 months of follow-up after the last treatment[1].<br><br>**Secondary outcome(s)**<br>Initial treatment success[2] at 3 months after the last treatment, AEs, adherence, patient satisfaction with treatment, health-related quality of life, and cosmetic results. | potential for retreatment 12 weeks later | Gender (male)<br>136 (87.7)<br><br>Lesion count<br>Median: 16; Range: 5 to 48 |
| | | | | **IMQ5%:** One 4-week treatment course, with potential for retreatment 4 weeks later | Age<br>Median: 73; Range: 59 to 89<br><br>Gender (male)<br>143 (91.7)<br><br>Lesion count<br>Median: 16.5; Range: 5 to 37 |
| | | | | **MAL_PDT:** 1 session, with potential for retreatment at 12 weeks | Age<br>Median: 73; Range: 55 to 90 |

[1] Treatment failure was defined as a reduction of less than 75% in the number of actinic keratosis lesions counted at baseline and could occur at 3 months after the last treatment (initial failure) or at 12 months after initial successful treatment.
[2] Treatment success was defined as ≥75% reduction from baseline in the number of actinic keratosis lesions

| Study identifier, duration of trial**, and location | Participants randomised | Trial design (as described by authors) | Primary and secondary outcomes and outcome measures | Duration of treatment | Patient characteristics<br><br>Age *(mean (SD) in years)*<br><br>Gender *(N (%) male)*<br><br>Lesion count per patient at baseline *(mean (SD))* |
|---|---|---|---|---|---|
| | | | | | Gender (male)<br>140 (89.7)<br><br>Lesion count<br>Median: 16; Range: 5 to 38 |
| | | | | **IM0.015%:** One 3-day course, with potential for retreatment 12 weeks later | Age<br>Median: 72; Range: 51 to 94<br><br>Gender (male)<br>139 (88.5)<br><br>Lesion count<br>Median: 15; Range: 5 to 40 |
| Jorizzo 2007 [60]<br><br>12 months (only 8 weeks outcomes eligible)<br><br>13 USA study centres | 246 | A randomised, vehicle-controlled study<br><br>Double-blind | **Primary outcome(s)**<br>Complete clearance<br><br>**Secondary outcome(s)**<br>• Partial clearance (75% reduction)<br>• Individual AK lesion clearance rate<br>• AEs and local skin reactions (LSRs)<br>• Clinical laboratory tests (haematology and chemistry blood tests, and urinalysis), and the culture of suggested skin infections. | **IMQ5%:** One 4-week course, with the potential for a second course 4 weeks later<br><br>**PLAC_TOP:** One 4-week course, with the potential for a second course 4 weeks later | Age and gender NR; lesion count overall median across arms: 6 |
| Korman 2005 [57] | 492 | | **Primary outcome(s)** | **IMQ5%:** 16 weeks | Age |

| Study identifier, duration of trial**, and location | Participants randomised | Trial design (as described by authors) | Primary and secondary outcomes and outcome measures | Duration of treatment | **Patient characteristics**<br><br>**Age** *(mean (SD) in years)*<br><br>**Gender** *(N (%) male)*<br><br>**Lesion count per patient at baseline** *(mean (SD))* |
|---|---|---|---|---|---|
| 24 weeks<br><br>26 USA study centres | | Two independent, randomised, parallel-group, vehicle-controlled studies<br><br>Double-blind | Complete clearance.<br><br>**Secondary outcome(s)**<br>• Partial clearance rate, (75% reduction)<br>• AEs<br>• Local skin reactions<br>• Clinical laboratory tests<br>• Skin quality assessment | | 66.7 (10.6); Range: 41 to 87<br><br>Gender (male)<br>210 (86.6)<br><br>Lesion count NR |
| | | | | **PLAC_TOP:** 16 weeks | Age<br>65.9 (9.9); Range: 41 to 93<br><br>Gender (male)<br>221 (88.4)<br><br>Lesion count NR |
| Kose 2008 [88]<br><br>12 months<br><br>Department of Dermatology, Gulhane School of Medicine, Ankara 06018, Turkey | 49 | Single center, randomised controlled study<br><br>Open-label | **Primary outcome(s)**<br>≥75% AK clearance<br><br>**Secondary outcome(s)**<br>• Investigator and Patient Global Improvement Indices (IGII and PGII)<br>• Baseline Severity Index<br>• Tolerability<br>• Safety<br>• Routine laboratory tests (complete blood cell counts, urine analysis, and fasting chemistry) | **DICLO3%:** 12 weeks | Age<br>57.6 (10.6)<br><br>Gender (male)<br>12 (50.0*)<br><br>Lesion count NR |
| | | | | **IMQ5%:** 12 weeks | Age<br>56.5 (9.8)<br><br>Gender (male)<br>12 (48.0*) |

| Study identifier, duration of trial**, and location | Participants randomised | Trial design (as described by authors) | Primary and secondary outcomes and outcome measures | Duration of treatment | **Patient characteristics**<br><br>**Age** ***(mean (SD) in years)***<br><br>**Gender** ***(N (%) male)***<br><br>**Lesion count per patient at baseline** ***(mean (SD))*** |
|---|---|---|---|---|---|
| | | | | | Lesion count NR |
| Lebwohl 2004 [56]<br><br>24 weeks<br><br>24 centres in the USA and Canada | 436 | Two large, randomised, parallel group, vehicle-controlled trials<br><br>Double-blind | **Primary outcome(s)**<br>Complete clearance<br><br>**Secondary outcome(s)**<br>Partial clearance rate (75% reduction). Other outcomes were AEs; local skin reactions; clinical laboratory tests; skin quality assessment | **IMQ5%:** 16 weeks | Age<br>66.6 (10.6); Range: 39 to 88<br><br>Gender (male)<br>187 (87)<br><br>Lesion count<br>Median: 6; Range: 4 to 10 |
| | | | | **PLAC_TOP:** 16 weeks | Age<br>65.5 (9.8); Range: 37 to 88<br><br>Gender (male)<br>193 (87.3)<br><br>Lesion count<br>Median: 6; Range: 3 to 9 |
| Peplin, 2008: NCT00700063 [65]<br><br>8 weeks | 131 | Multicenter, randomised, vehicle-controlled, dose-ranging study | **Primary outcome(s)**<br>AEs, complete clearance<br><br>**Secondary outcome(s)**<br>Partial clearance | **IM0.015%:** Two days' treatment | Age<br>67.8 (7.0)<br><br>Gender (male)<br>32 (97) |

| Study identifier, duration of trial**, and location | Participants randomised | Trial design (as described by authors) | Primary and secondary outcomes and outcome measures | Duration of treatment | Patient characteristics<br><br>Age *(mean (SD) in years)*<br><br>Gender *(N (%) male)*<br><br>Lesion count per patient at baseline *(mean (SD))* |
|---|---|---|---|---|---|
| 25 centres in the USA and 3 centres in Australia | | Double-blind | | | Lesion count NR |
| | | | | **IM0.015%:** Three days' treatment | Age<br>65.2 (9.7)<br><br>Gender (male)<br>29 (87.9)<br><br>Lesion count NR |
| | | | | **PLAC_TOP:** Two days' treatment | Age<br>67.2 (10.1)<br><br>Gender (male)<br>28 (87.5)<br><br>Lesion count NR |
| | | | | **PLAC_TOP:** Three days' treatment | Age<br>69.3 (10.7)<br><br>Gender (male)<br>28 (84.8)<br><br>Lesion count NR |
| MEDA Pharma GmbH & Co. KG, 2015: NCT00777127 | 261 | Randomised, active controlled, multicentre, multinational study | **Primary outcome(s)**<br>Recurrence of AK<br><br>**Secondary outcome(s)** | **IMQ5%:** Each course could be either: 4 weeks' treatment, 4 weeks off, 4 weeks' treatment, OR 4 weeks' treatment, 8 weeks | Age<br>Overall: 70.88 (7.6)<br><br>Gender (male)<br>Overall: 227 (87.3) |

| Study identifier, duration of trial**, and location | Participants randomised | Trial design (as described by authors) | Primary and secondary outcomes and outcome measures | Duration of treatment | Patient characteristics<br><br>**Age** *(mean (SD) in years)*<br><br>**Gender** *(N (%) male)*<br><br>**Lesion count per patient at baseline** *(mean (SD))* |
|---|---|---|---|---|---|
| EUCTR-2007-004884-24<br><br>LEIDA (X-03016-3271)<br><br>[76]<br><br>36 months<br><br>26 centres in Germany, Austria, and France | | Open-label | Incidence of squamous cell carcinoma. Cosmetic outcome determined by patient and investigator. Complete and partial clinical clearance; AEs and LSRs | off. Patients received between 1 and 6 courses.<br><br>**DICLO3%:** Each course contained 12 weeks' treatment. Patients received between 1 and 6 courses. | Lesion count<br>Overall: Median 7.0 |
| Taro Pharmaceuticals USA, 2008: NCT00828568 [80]<br><br>24 weeks<br><br>20 centres in the USA | 246 | Randomised, parallel-group, vehicle-controlled therapeutic equivalence study<br><br>Double-blind | **Primary outcome(s)**<br>Complete clearance<br><br>**Secondary outcome(s)**<br>AEs | **IMQ5%:** 16 weeks | Age<br>68.0 (9.5)<br><br>Gender (male)<br>149 (81.4)<br><br>Lesion count NR |
| | | | | **PLAC_TOP:** 16 weeks | Age<br>64.7 (9.7)<br><br>Gender (male)<br>52 (86.7)<br><br>Lesion count NR |

| **Study identifier, duration of trial**, and location** | **Participants randomised** | **Trial design (as described by authors)** | **Primary and secondary outcomes and outcome measures** | **Duration of treatment** | **Patient characteristics**<br><br>**Age** *(mean (SD) in years)*<br><br>**Gender** *(N (%) male)*<br><br>**Lesion count per patient at baseline** *(mean (SD))* |
|---|---|---|---|---|---|
| Peplin, 2009a: NCT00915551 [66]<br><br>8 weeks<br><br>19 centres in the USA and 2 in Australia | 278 | Multicenter, randomised, parallel group, vehicle-controlled study<br><br>Double-blind | **Primary outcome(s)**<br>Complete clearance<br><br>**Secondary outcome(s)**<br>Partial clearance defined as ≥75% reduction in the number of AK lesions identified at baseline in the treatment area | **IM0.015%:** 3 days | Age<br>Between 18 and 65 years: 73 (51.4)<br>≥65 years: 69 (48.6)<br><br>Gender (male)<br>117 (82.4)<br><br>Lesion count NR |
| | | | | **PLAC_TOP:** 3 days | Age<br>Between 18 and 65 years: 63 (46.3)<br>≥65 years: 73 (53.7)<br><br>Gender (male)<br>112 (82.4)<br><br>Lesion count NR |
| Peplin, 2009b: NCT00916006 [67]<br><br>8 weeks<br><br>19 centres in the USA and 2 in Australia | 269 | Multicenter, randomised, parallel group, vehicle-controlled study<br><br>Double-blind | **Primary outcome(s)**<br>Complete clearance<br><br>**Secondary outcome(s)**<br>Partial clearance defined as ≥75% reduction in the number of AK lesions identified at baseline in the treatment area | **IM0.015%:** 3 days | Age<br>Between 18 and 65 years: 71 (52.6)<br>≥65 years: 64 (47.4)<br><br>Gender (male)<br>116 (85.9)<br><br>Lesion count NR |

| **Study identifier, duration of trial**, and location** | **Participants randomised** | **Trial design (as described by authors)** | **Primary and secondary outcomes and outcome measures** | **Duration of treatment** | **Patient characteristics**<br><br>**Age** *(mean (SD) in years)*<br><br>**Gender** *(N (%) male)*<br><br>**Lesion count per patient at baseline** *(mean (SD))* |
|---|---|---|---|---|---|
| | | | | **PLAC_TOP:** 3 days | Age<br>Between 18 and 65 years: 77 (57.5)<br>≥65 years: 57 (42.5)<br><br>Gender (male)<br>120 (89.6)<br><br>Lesion count NR |
| Gage Development Company, 2016: NCT02952898 [68]<br><br>13 weeks<br><br>16 centres in the USA | 445 | Multicenter, randomised, placebo-controlled, parallel group comparison study<br><br>Double-blind | **Primary outcome(s)**<br>Complete clearance<br><br>**Secondary outcome(s)**<br>AEs | **DICLO3%:** 60 days | Age<br>69.6 (9.8)<br><br>Gender (male)<br>175 (79.5)<br><br>Lesion count NR |
| | | | | **PLAC_TOP:** 60 days | Age<br>69.1 (9.3)<br><br>Gender (male)<br>187 (83.9)<br><br>Lesion count NR |
| Actavis Inc, 2016: NCT03200912 [69]<br><br>8 weeks | 337 | Multicenter, randomised, vehicle-controlled | **Primary outcome(s)**<br>Complete clearance<br><br>**Secondary outcome(s)** | **IM0.015%:** 3 days | Age<br>68.9 (8.6)<br><br>Gender (male) |

| Study identifier, duration of trial**, and location | Participants randomised | Trial design (as described by authors) | Primary and secondary outcomes and outcome measures | Duration of treatment | **Patient characteristics**<br><br>**Age** ***(mean (SD) in years)***<br><br>**Gender** ***(N (%) male)***<br><br>**Lesion count per patient at baseline** ***(mean (SD))*** |
|---|---|---|---|---|---|
| 23 centres in the USA | | parallel comparison study<br><br>Double-blind | NR | | 137 (81.1)<br><br>Lesion count NR |
| | | | | **PLAC_TOP:** 3 days | Age<br>70.2 (9.4)<br><br>Gender (male)<br>133 (79.2)<br><br>Lesion count NR |
| Pariser 2003: NCT00306800 [64]<br><br>13 weeks<br><br>5 centres in the USA | 80 | Multicenter, randomised<br><br>Double-blind | **Primary outcome(s)**<br>Patient complete response rate<br><br>**Secondary outcome(s)**<br>Cosmetic outcome, patient satisfaction, safety | **MAL_PDT:** Two treatments, one week apart | Age<br>64; Range: 31 to 84<br><br>Gender (male)<br>36 (85.7*)<br><br>Lesion count<br>Total: 260<br>Mean: 6.2 per patient |
| | | | | **PLAC_PDT:** Two treatments, one week apart | Age<br>67; Range: 39 to 84<br><br>Gender (male)<br>34 (89.5*)<br><br>Lesion count<br>Total: 242 |

| **Study identifier, duration of trial**, and location** | **Participants randomised** | **Trial design (as described by authors)** | **Primary and secondary outcomes and outcome measures** | **Duration of treatment** | **Patient characteristics**<br><br>**Age** ***(mean (SD) in years)***<br><br>**Gender** ***(N (%) male)***<br><br>**Lesion count per patient at baseline** ***(mean (SD))*** |
|---|---|---|---|---|---|
| | | | | | Mean: 6.4 per patient |
| Pariser 2008 [72]<br><br>13 weeks<br><br>8 centres in the USA | 96 | Randomised, vehicle-controlled,<br><br>Double-blind | **Primary outcome(s)**<br>Lesion complete response (complete disappearance of the lesion); patient complete response; new lesions<br><br>**Secondary outcome(s)**<br>NR | **MAL_PDT:** Two sessions of PDT one week apart | Age<br>66.1; Range: 43 to 86<br><br>Gender (male)<br>42 (85.7*)<br><br>Lesion count<br>Median: 8; Range: 4 to 10 |
| | | | | **PLAC_PDT:** Two sessions of PDT one week apart | Age<br>66.7; Range: 48 to 89<br><br>Gender (male)<br>37 (78.7*)<br><br>Lesion count<br>Median: 8; Range: 4 to 10 |
| Pariser 2016: NCT01475955 [74]<br><br>24 weeks<br><br>13 sites in the USA | 93 | Randomised vehicle-controlled study<br><br>Single-blind | **Primary outcome(s)**<br>Lesion clearance; patient complete (100%) clearance rate, partial (≥75%) clearance rate, and subject satisfaction and acceptability of treatment. Tolerability assessments<br><br>**Secondary outcome(s)** | **ALA_PDT:** One session with potential for a second session 8 weeks later | Age<br>66.4 (10.6)<br><br>Gender (male)<br>40 (85.1)<br><br>Lesion count |

| Study identifier, duration of trial**, and location | Participants randomised | Trial design (as described by authors) | Primary and secondary outcomes and outcome measures | Duration of treatment | Patient characteristics<br><br>Age *(mean (SD) in years)*<br><br>Gender *(N (%) male)*<br><br>Lesion count per patient at baseline *(mean (SD))* |
|---|---|---|---|---|---|
| | | | NR | | Median: 13.0; Range: 6 to 20 |
| | | | | **PLAC_PDT:** One session with potential for a second session 8 weeks later | Age<br>68.4 (9.4)<br><br>Gender (male)<br>40 (87.0)<br><br>Lesion count<br>Median: 14.0; Range: 7 to 20 |
| Piacquadio 2004 [85]<br><br>12 weeks<br><br>16 sites in the USA | 243 | This was a multicentre, investigator-blinded, randomised, vehicle-controlled, uneven parallel-group study. The results from 2 independent, identical, phase 3 clinical trials are presented as a single research effort | **Primary outcome(s)**<br>Safety was assessed by analyses of AEs, PDT response, pigmentary changes, and laboratory results. Efficacy was assessed using (1) the complete response rate or clearing of individual AK lesions, (2) the percentage of patients who experienced 75% or greater clearance of all target AKs treated (75% response rate), and (3) the percentage of patients who experienced 100% clearance of all target AKs treated (100% response rate).<br><br>**Secondary outcome(s)**<br>NR | **ALA_PDT:** One session with potential for a second session at 8 weeks | Age<br>67.1; Range: 34 to 89<br><br>Gender (male)<br>147 (81)<br><br>Lesion count NR |
| | | | | **PLAC_PDT:** One session with potential for a second session at 8 weeks | Age<br>64.5; Range: 35 to 85<br><br>Gender (male)<br>56 (90)<br><br>Lesion count NR |

| **Study identifier, duration of trial**, and location** | **Participants randomised** | **Trial design (as described by authors)** | **Primary and secondary outcomes and outcome measures** | **Duration of treatment** | **Patient characteristics**<br><br>**Age** ***(mean (SD) in years)***<br><br>**Gender** ***(N (%) male)***<br><br>**Lesion count per patient at baseline** ***(mean (SD))*** |
|---|---|---|---|---|---|
| | | Single-blind | | | |
| Reinhold 2016 [52]<br><br>24 weeks<br><br>7 centres in Germany | 87 | Randomised, multicentre, placebo-controlled, parallel-group trial<br><br>Double-blind | **Primary outcome(s)**<br>Complete clearance<br><br>**Secondary outcome(s)**<br>Cosmetic outcome, safety and tolerability | **ALA_PDT:** One session with the potential for a second session 12 weeks later | Age<br>71.9 (6.4)<br><br>Gender (male)<br>50 (91)<br><br>Lesion count<br>5.4 (1.0) |
| | | | | **PLAC_PDT:** One session with the potential for a second session 12 weeks later | Age<br>71.0 (6.4)<br><br>Gender (male)<br>29 (91)<br><br>Lesion count<br>5.4 (1.2) |
| Samorano 2015: NCT02242747 [87]<br><br>7 weeks<br><br>Hospital das Clínicas of the University of São | 100 | Prospective single-site RCT<br><br>Open-label | **Primary outcome(s)**<br>Total local skin reaction (LSR) grading scale values; adverse effects related to drug treatment<br><br>**Secondary outcome(s)**<br>To evaluate and compare the area under the curve for each component of the total local skin reaction score using generalised linear models | **IM0.015%:** 3 days<br><br>**5FU5%:** 4 weeks | Age<br>Overall mean: 74;<br>Range: 38 to 95<br><br>Gender (male)<br>Overall: 35 (35)<br><br>Lesion count NR |

| Study identifier, duration of trial**, and location | Participants randomised | Trial design (as described by authors) | Primary and secondary outcomes and outcome measures | Duration of treatment | Patient characteristics<br><br>Age *(mean (SD) in years)*<br><br>Gender *(N (%) male)*<br><br>Lesion count per patient at baseline *(mean (SD))* |
|---|---|---|---|---|---|
| Paulo Medical School, Brazil | | | | | |
| Serra-Guillen 2012 [92]<br><br>8 weeks<br><br>Dermatology Service of the Instituto Valenciano de Oncología, Spain | NR | Randomised pilot study<br><br>Open-label | **Primary outcome(s)**<br>Complete clinical response<br><br>**Secondary outcome(s)**<br>Partial response (75% or more reduction in lesions); complete clinicopathologic response = lack of AK in the biopsy specimen | **MAL_PDT:** One session | Age<br>72.7<br><br>Gender (male)<br>37 (9.25*)<br><br>Lesion count<br>9.0 (2.7) |
| | | | | **IMQ5%:** 4 weeks | Age<br>74.3<br><br>Gender (male)<br>28 (84.8*)<br><br>Lesion count<br>9.4 (3.1) |
| Simon 2015: NCT01358851 [89]<br><br>32 weeks<br><br>4 centres in Germany | 67 | An exploratory, open, randomised, prospective, two-armed study<br><br>Open-label | **Primary outcome(s)**<br>Percentage of patients with histological clearance of a predefined target lesion at 8 weeks<br><br>**Secondary outcome(s)**<br>Lesion counts, lesion response at each study visit, AK clearance rate (complete and partial (>75%)), determined by clinical evaluation, the physicians' | **5FU0.5%+SA:** 6 weeks | Age<br>70.6 (8.3); Range: 51 to 83<br><br>Gender (male)<br>29 (87.9)<br><br>Lesion count<br>8.1 (1.2) |

| Study identifier, duration of trial**, and location | Participants randomised | Trial design (as described by authors) | Primary and secondary outcomes and outcome measures | Duration of treatment | Patient characteristics<br><br>**Age** *(mean (SD) in years)*<br><br>**Gender** *(N (%) male)*<br><br>**Lesion count per patient at baseline** *(mean (SD))* |
|---|---|---|---|---|---|
| | | | global assessment, cosmetic outcome and patients' assessment of clinical improvement. | **CRYO:** 1 session, with potential for a second session within 3 weeks (88% of patients had a second session) | Age<br>71.3 (7.6); Range: 53 to 85<br><br>Gender (male)<br>29 (87.9)<br><br>Lesion count<br>8.0 (1.1) |
| Stockfleth 2017: NCT02289768 [62]<br><br>20 weeks<br><br>14 sites in Germany and the UK | 166 | Multicentre, vehicle-controlled study<br><br>Double-blind | **Primary outcome(s)**<br>Complete clinical clearance<br><br>**Secondary outcome(s)**<br>• Partial clearance (≥75% reduction in clinically visible AK lesions)<br>• Change from baseline in the total number of AK lesions<br>• Number of lesions by AK grade severity<br>• Proportional change from baseline in the total number of lesions<br>• Global assessment of efficacy: Physician Global Assessment (PGA)<br>• Patient-reported outcomes included patient satisfaction with treatment by recording individual domain scores of the Treatment Satisfaction Questionnaire for Medication (TSQM, version 1.4) | **5FU0.5%+SA:** 12 weeks | Age<br>71.8 (7.3)<br><br>Gender (male)<br>92 (85.2)<br><br>Lesion count<br>5.6 (1.4) |
| | | | | **PLAC_TOP:** 12 weeks | Age<br>72.8 (6.9)<br><br>Gender (male)<br>51 (92.7)<br><br>Lesion count<br>5.6 (1.5) |

| **Study identifier, duration of trial**, and location** | **Participants randomised** | **Trial design (as described by authors)** | **Primary and secondary outcomes and outcome measures** | **Duration of treatment** | **Patient characteristics**<br><br>**Age** ***(mean (SD) in years)***<br><br>**Gender** ***(N (%) male)***<br><br>**Lesion count per patient at baseline** ***(mean (SD))*** |
|---|---|---|---|---|---|
| | | | • Quality-of-life assessment by recording the change from baseline in total and individual domain scores of the Dermatology Life Quality Index (DLQI)<br>• AEs | | |
| Stockfleth 2018 [75]<br><br>17 weeks<br><br>33 sites in Germany, Spain and the UK | 502 | Multicentre<br><br>Open-label | **Primary outcome(s)**<br>Complete clearance<br><br>**Secondary outcome(s)**<br>75% clearance; AEs | **IM0.015%:** One 3-day course, with potential for retreatment at 8 weeks | Age<br>Median: 75; Range: 49 to 95<br><br>Gender (male)<br>216 (84.7)<br><br>Lesion count<br>Median: 6; Range 4 to 8 |
| | | | | **DICLO3%:** 90 days | Age<br>Median: 75; Range: 34 to 96<br><br>Gender (male)<br>212 (85.8)<br><br>Lesion count<br>Median: 6; Range: 4 to 9 |
| Swanson 2010: NCT00605176 [59]<br><br>14 weeks | 319 | Two randomised, placebo-controlled studies | **Primary outcome(s)**<br>Complete clearance<br><br>**Secondary outcome(s)** | **IMQ3.75%**: Two 2-week treatment cycles; i.e. 2 weeks' treatment, 2 weeks off, 2 weeks' treatment | Age<br>64.5 (10.6)<br><br>Gender (male)<br>132 (82.5) |

| Study identifier, duration of trial**, and location | Participants randomised | Trial design (as described by authors) | Primary and secondary outcomes and outcome measures | Duration of treatment | Patient characteristics<br><br>Age *(mean (SD) in years)*<br><br>Gender *(N (%) male)*<br><br>Lesion count per patient at baseline *(mean (SD))* |
|---|---|---|---|---|---|
| 25 study centres in the USA | | Double-blind | • 75% reduction in the number of AK lesions in the treatment area compared with baseline<br>• Percent change from baseline in AK lesion count<br>• Local skin reactions<br>• Investigator Global Integrated Photodamage (IGIP) score | | Lesion count<br>11.0 (4.8) |
| | | | | **PLAC_TOP:** Two 2-week treatment cycles; i.e. 2 weeks' treatment, 2 weeks off, 2 weeks' treatment | Age<br>64.3 (8.9)<br><br>Gender (male)<br>130 (81.8)<br><br>Lesion count<br>11.3 (4.7) |
| Szeimies 2004 [55]<br><br>24 weeks<br><br>18 European centres (3 in France, 5 in Germany, 3 in Italy, 1 in the Netherlands, 3 in Spain, and 3 in the UK) | 286 | Multicentre, parallel group, vehicle-controlled RCT<br><br>Double-blind | **Primary outcome(s)**<br>Complete clearance rate<br><br>**Secondary outcome(s)**<br>Partial clearance rate | **IMQ5%:** 16 weeks | Age<br>71.1 (7.9)<br><br>Gender (male)<br>131 (89.1)<br><br>Lesion count NR |
| | | | | **PLAC_TOP:** 16 weeks | Age<br>70.9 (8.4)<br><br>Gender (male)<br>117 (84.2)<br><br>Lesion count NR |
| Szeimies 2010 [82] | 122 | Randomised, multicentre, | **Primary outcome(s)**<br>Complete clearance | **ALA_PDT:** One session with the potential for a | Age<br>70.4 (5.1) |

| Study identifier, duration of trial**, and location | Participants randomised | Trial design (as described by authors) | Primary and secondary outcomes and outcome measures | Duration of treatment | **Patient characteristics**<br><br>**Age** *(mean (SD) in years)*<br><br>**Gender** *(N (%) male)*<br><br>**Lesion count per patient at baseline** *(mean (SD))* |
|---|---|---|---|---|---|
| 24 weeks<br><br>8 study centres in Germany | | placebo-controlled, inter-individual, two-armed trial<br><br>Double-blind | **Secondary outcome(s)**<br>Safety and tolerability | second session 12 weeks later | Gender (male)<br>73 (90.1)<br><br>Lesion count<br>5.7 |
| | | | | **PLAC_PDT:** One session with the potential for a second session 12 weeks later | Age<br>70.6 (6.6)<br><br>Gender (male)<br>32 (78.0)<br><br>Lesion count<br>5.5 |
| Tanghetti 2007 [83]<br><br>24 weeks<br><br>2 centres; Centre for Dermatology and Laser Surgery, Sacramento, CA and Dermatology, University of Washington, Seattle, WA, USA | 39 | 2-centre, physician-blinded randomised study<br><br>Single-blind | **Primary outcome(s)**<br>AK count, % change in AK count, complete clearance, physician's global assessment, patient perception of efficacy, physician's assessment of erythema, patient perception of discomfort<br><br>**Secondary outcome(s)**<br>NR | **5FU5%:** 2 to 4 weeks | Age, gender and lesion count NR |
| | | | | **IMQ5%:** 16 weeks | Age, gender and lesion count NR |
| Zane 2014 [91] | 200 | | **Primary outcome(s)** | **DICLO3%:** 90 days | Age |

| Study identifier, duration of trial**, and location | Participants randomised | Trial design (as described by authors) | Primary and secondary outcomes and outcome measures | Duration of treatment | Patient characteristics<br><br>**Age** *(mean (SD) in years)*<br><br>**Gender** *(N (%) male)*<br><br>**Lesion count per patient at baseline** *(mean (SD))* |
|---|---|---|---|---|---|
| 12 months<br><br>Dermatology Department of the University of Brescia, Italy | | Single-centre, prospective, non sponsored, randomised controlled clinical trial<br><br>Open-label | Complete clearance, partial clearance, AEs, cosmetic outcome, cost-effectiveness<br><br>**Secondary outcome(s)**<br>NR | | 72 (8)<br><br>Gender (male)<br>76 (76)<br><br>Lesion count<br>8.05 |
| | | | | **MAL_PDT:** One session, with potential for a second session 12 weeks later | Age<br>74 (9)<br><br>Gender (male)<br>66 (66)<br><br>Lesion count<br>8.69* |

* Indicates value calculated by reviewers

** "Duration of trial" defined as treatment plus follow up, figures are standardised into weeks / months and are taken from the beginning of treatment

AE - Adverse event; AG – Aktiengesellschaft (joint-stock company); AK - Actinic keratosis; ALA - Aminolevulinic acid; CA - California; CLNS - Cumulative lesion number score; CRYO – cryosurgery; DHA - Diclofenac plus hyaluronate gel; DICLO - Diclofenac; DLQI - Dermatology Life Quality Index; EUCTR - EU Clinical Trials Register; FAS – full analysis set; 5FU - 5-Fluorouracil; HFUS - High-frequency ultrasound; IGII - Investigator Global Improvement Indices; IGIP - Investigator Global Integrated Photodamage; IM - Ingenol mebutate; IMQ - Imiquimod; ITT - Intent to Treat; KG – Kommanditgesellschaft (limited commercial partnership); LED – light-emiting diode; LEIDA - Long-term Effects of Imiquimod and Diclofenac in Actinic Keratoses; LLC – limited liability company; LSR - Local skin reaction; MAL - Methyl aminolevulinate; NR - Not reported; PDT - Photodynamic therapy; PGA - Physician Global Assessment; PGII - Patient Global Improvement Indices; PGT - Physician Global Assessment; PP – per protocol; RCT - Randomised controlled trial; SA - Salicylic acid; SCC - Squamous cell carcinoma; TIRBA1% – Tirbanibulin; TLNS - Target lesion number score; TOP – Topical; TSQM - Treatment Satisfaction Questionnaire for Medication; UK - United Kingdom; USA - United States of America; VEH - Vehicle; WA – Washington.

## 1.2 Data extraction elements

For each study, information was collected on:

- Trial details (bibliographic details)
- Trial characteristics:
  - Trial design
  - Trial objective
  - Number of participating centres
  - Country
  - Eligibility criteria
  - Number of patients randomised/analysed
  - Treatment duration
  - Follow up duration
  - Data collection time points
- Patient baseline characteristics:
  - Age
  - Gender
  - Time since diagnosis
  - Location and area of AK
  - Severity of AK
  - Lesion count at baseline
  - Number and details of prior treatments for AK
  - Fitzpatrick skin type
  - History of skin cancer
  - Details of any current immunosuppression
- Details of intervention and placebo
  - Treatment
  - Dose
  - Regimen
  - Duration of treatment
  - Concomitant medications, including sunscreen use
- Details of statistical analyses
- For each of the outcomes specified we will extract the following:
  - Outcome definition
  - The unit of measurement
  - The number of patients included in the analysis
  - The size of the effect:
    - For dichotomous outcomes; absolute and relative risks (or ORs) and risk (or rate) differences
    - For continuous outcomes; the mean change and measure of variance from baseline (or at both baseline and final visit), or mean difference between treatments
    - For time-to-event analysis; the number of events in each arm, median time to event and a hazard ratio and p-value
    - Where possible, absolute and relative data were extracted

- A measure of precision for each estimate of effect (95% confidence intervals, standard error or standard deviation)

## 1.3 Details of the PRISMA flow diagram

The following provides additional information on the reasons for exclusion of documents from the systematic review.

Abstract - cannot link to full text paper: Document is an abstract that appeared to be reporting an eligible study, but the study could not be linked (using details of patient N, interventions, location, and any reported clinical trial numbers or study names) to any study reported in an included full text paper. Studies only reported in abstract form did not offer sufficient information to include them in the review.

Completed trial (no results): Document is a clinical trial record reporting the methods of an apparently eligible clinical trial. Based on the dates supplied, the trial appears to be completed. However, no results were reported in the trial record and the trial could not be linked (using details of patient N, interventions, location, and any reported clinical trial numbers or study names) to any study reported in an included full text paper.

Does not report comparative results: Document is a paper reporting the conduct of eligible studies, but results are not presented for both arms of the study.

Duplicate record: Document is an exact duplicate of an already-included document, e.g. this may be the same record downloaded twice from different databases.

Ineligible publication type: Document does not report a clinical study, e.g. a letter to editor or report of a non-systematic review.

Ineligible subgroup analysis: Document reports an eligible study but the only results reported are for a subgroup analysis of a patient subgroup not of interest to this systematic review.

Insufficient information to include: Document does not report sufficient information to judge whether the study reported is eligible or not.

Possible ongoing trial (no results): Document is a clinical trial record reporting the methods of an apparently eligible clinical trial. Based on the dates supplied, the trial appears to be ongoing: no results are reported.

Review, non-systematic: Narrative (non-systematic) reviews of studies were not eligible for inclusion or reference checking.

SR for reference checking: Systematic reviews of treatments for actinic keratosis were excluded but the lists of included studies were checked to ensure all relevant studies had been identified.

Document unobtainable: Document was unavailable for purchase and subsequent screening.

Ineligible comparator: Document reports a study assessing an eligible intervention (i.e. one listed in the PICO for this systematic review) against a non-eligible comparator.

Ineligible intervention: Document reports a study assessing a non-eligible intervention (i.e. one other than those listed in the PICO for this systematic review).

Ineligible outcomes: Document reports a study that did not assess / report any outcomes of interest listed in the PICO for this systematic review.

Ineligible patient population: Document reports a study assessing treatments for AK in a population other than that detailed in the PICO for this systematic review, e.g. children or organ transplant patients.

Ineligible study design: Document reports a clinical study that is not an interindividual RCT, e.g. a retrospective study or an intraindividual RCT.

# 2 Selection of studies for NMA

## 2.1 Results of the feasibility assessment

Following a comparison of the populations, interventions, comparators and designs of the 46 trials included in the review, six studies were deemed to be unsuitable for inclusion in an NMA.

Dermapharm AG, 2011: EUCTR- 2011-003317-41 [94] was removed from the feasibility assessment as the lack of reporting of the number of patients per arm meant the study could not be used in a NMA.

Both MEDA Pharma studies [76, 90] were removed from consideration for the NMA as the number of courses of treatment assessed by both arms (DICLO3% and IMQ5%_EU) of these studies was not sufficiently similar to the rest of the studies included in the review to allow for pooling.

Berman 2014 [93] treated all patients with two sessions of MAL_PDT, four weeks apart, while the US label for Levulan Kerastick states that retreatment should be after eight weeks, and should only be for those patients for whom it is necessary. The study was therefore excluded from consideration for the NMA as the scheduling was not as per label.

The DICLO3% arm of Kose 2008 [88] was removed from consideration for the analyses, as it assessed one-daily treatment with DICLO3%. This is both off-label, and insufficiently comparable with the rest of the included studies of diclofenac, all of which assessed application

twice daily. The IMQ5%_USA arm of Kose 2008 was also removed from consideration for the analyses, as it assessed treatment for 12 weeks, rather than the 16 weeks both as suggested by the US label and as assessed by the other studies of the US scheduling of IMQ5%. As Kose 2008 is a two-armed trial it was therefore removed at this stage.

The IMQ3.75% arm of Hanke 2010 [58] was excluded from consideration for the analyses as it assesses a length of treatment other than that suggested by the relevant labelling for this intervention. As Hanke is a two-armed trial the whole trial was therefore removed from consideration for the NMA.

Placebo / vehicle arms were grouped into topical placebo (PLAC_TOP) or placebo PDT (PLAC_PDT). While this is an assumption around the equivalence of the included placebo / vehicle arms (for example, it does not take into consideration the fact that the hyaluronic acid vehicle for DICLO3% may have some efficacy of its own), this was necessary to form connected networks. The assumption of placebo equivalence is also consistent with the practices of previous NMA authors [21, 22, 47, 48], and by separating PLAC_TOP and PLAC_PDT into separate nodes, this NMA retains a greater degree of differentiation than prior NMAs which grouped PLAC_TOP and PLAC_PDT into one node [21, 22, 48].

## 2.2 Selection of studies for the European analyses

Of the 40 studies remaining for use in the analyses after the feasibility assessment [28, 51-57, 59-75, 77-87, 89, 91, 92], five studies were not relevant to a European perspective [55-57, 80, 83] as they assessed the US scheduling of imiquimod 5% against another intervention or placebo. Following the removal of these further five studies, 35 studies remained eligible for use in the European analyses [28, 51-54, 59-75, 77-79, 81, 82, 84-87, 89, 91, 92].

Supplementary Table S6 provides a summary of the studies informing the qualitative analyses and the NMA base case and sensitivity analyses, assessed for the European perspective.

# Supplementary Table S6: Summary of studies contributing to the European perspective analyses

| Study identifier | NMA of complete clearance | | | | | Qualitative analyses | | |
|---|---|---|---|---|---|---|---|---|
| | Base case European analysis | Sensitivity analyses | | | | Lesion count reduction | Discontinuations due to TRAEs / TEAEs / local AEs / LSRs | Number of severe LSRs |
| | Any number of courses | One treatment cycle | Treatment area limited to 25 cm$^2$ | Single placebo node | Response assessment ≥8wks after treatment | | | |
| Actavis Inc, 2016: NCT03200912 | x | x | x | x | x | | | |
| Almirall Hermal GmbH, 2007: EUCTR-2007-003889 | x | x | x | x | x | x | x | |
| Almirall S.A., 2010: EUCTR-2010-022244-20 | x | x | | x | x | | | |
| Alomar 2007 | x | | x | x | | | x | x |
| Arisi 2020 | x | x | x | x | x | x | | |
| Athenex, Inc 2019a KX01-AK-003: NCT03285477 | x | x | x | x | x | x | x | x |
| Athenex, Inc 2019b KX01-AK-004: NCT03285490 | x | x | x | x | x | x | x | x |
| Biofrontera Bioscience GmbH, 2006: EUCTR-2006-000314-20 | x | x | | x | | | x | |
| Dirschka 2012 | x | | | x | x | | x | |
| Dohil 2016 | x | x | | x | | x | | |
| Foley 2011 | x | | | x | x | | | |
| Gage Development Company, 2016: NCT02952898 | x | x | | x | | | | |
| Hauschild 2009 AK 03 | x | X (but does not link to main network) | | x | x | | x | |
| Hauschild 2009 AK 04 | x | X (but does not link to main network) | | x | x | | x | |
| Jorizzo 2007 | x | | x | x | | | | |
| Pariser 2003 | x | | | x | x | x | | |
| Pariser 2008 | x | | | x | x | | | |
| Pariser 2016 | x | | | x | x | | | x |

| Study identifier | NMA of complete clearance | | | | | Qualitative analyses | | |
|---|---|---|---|---|---|---|---|---|
| | **Base case European analysis** | **Sensitivity analyses** | | | | **Lesion count reduction** | **Discontinuations due to TRAEs / TEAEs / local AEs / LSRs** | **Number of severe LSRs** |
| | Any number of courses | One treatment cycle | Treatment area limited to 25 cm² | Single placebo node | Response assessment ≥8wks after treatment | | | |
| Peplin, 2008: NCT00700063 | x | x | | x | x | | | |
| Peplin, 2009a: NCT00915551 | x | x | x | x | x | | | |
| Peplin, 2009b: NCT00916006 | x | x | x | x | x | | | |
| Piacquadio 2004 | x | | | x | | | x | |
| Reinhold 2016 | x | | x (but does not link to main network) | x | x | x | x | |
| Serra-Guillen 2012 | x | x | x | x | | | | |
| Simon 2015 | x | | x | x | x | | x | |
| Stockfleth 2017 | x | x | x | x | x | x | x | |
| Stockfleth 2018 | x | | x | x | | x | x | |
| Swanson 2010 | x | | | x | x | x | x | x |
| Szeimies 2010 | x | | | x | x | | | |
| Zane 2014 | x | | | x | x | x | | |
| Chen 2003 | | | | | | | x | |
| Freeman 2003 | | | | | | | x | |
| Jansen 2019 | | | | | | | x | |
| **Studies eligible for European perspective analyses but reporting safety outcomes other than those described in this report** | | | | | | | | |
| Dermapharm AG, 2014: EUCTR- 2014-001621-33 | Reports overall serious adverse events, deaths, and LSRs (overall, not severe, so does not contribute to writeup) | | | | | | | |
| Samorano 2015 | Reports serious adverse events, and LSRs (overall, not severe, so does not contribute to writeup) | | | | | | | |
| **Studies assessing IMQ5%_USA excluded from the European perspective analyses** | | | | | | | | |
| Korman 2005 | | | | | | | | |
| Lebwohl 2004 | | | | | | | | |
| Szeimies 2004 | | | | | | | | |
| Tanghetti 2007 | | | | | | | | |
| Taro Pharmaceuticals USA, 2008: NCT00828568 | | | | | | | | |
| **Studies excluded from all analyses following feasibility assessment** | | | | | | | | |

| Study identifier | NMA of complete clearance | | | | | Qualitative analyses | | |
|---|---|---|---|---|---|---|---|---|
| | **Base case European analysis** | **Sensitivity analyses** | | | | **Lesion count reduction** | **Discontinuations due to TRAEs / TEAEs / local AEs / LSRs** | **Number of severe LSRs** |
| | Any number of courses | One treatment cycle | Treatment area limited to 25 $cm^2$ | Single placebo node | Response assessment ≥8wks after treatment | | | |
| Berman 2014 | Scheduling off label | | | | | | | |
| Dermapharm AG, 2011: EUCTR- 2011-003317-41 | Not enough data reported to use either arm; no N reported | | | | | | | |
| Hanke 2010 | Scheduling off label | | | | | | | |
| Kose 2008 | Both arms are off label, and one arm assesses IMQ5%_USA | | | | | | | |
| MEDA Pharma GmbH & Co. KG, 2015: NCT00777127, EUCTR-2007-004884-24, LEIDA (X-03016-3271) | Both arms offer more treatment than other studies of these interventions, so are not comparable | | | | | | | |
| MEDA Pharma GmbH & Co. KG, 2010: EUCTR-2010-022054-16 (LEIDA 2) | Both arms offer more treatment than other studies of these interventions, so are not comparable | | | | | | | |

AE – adverse event; LSR – local skin reaction; TEAE – treatment emergent adverse event; TRAE – treatment related adverse event

# 3 Detailed results of the qualitative analyses: Europe

The following analyses are from the European perspective only.

### 3.1.1 Lesion count reduction

Eleven studies eligible for inclusion in this analysis [52-54, 59, 62-64, 75, 79, 84, 91] reported data on lesion count reduction. Nine of the studies [52, 59, 62-64, 75, 79, 91] reported change as a percentage and did not include any variance data. The remaining three studies [53, 54, 84] reported a mean or median change rather than a percentage change.

Lesion count reductions of between 51.8% (DICLO3% [91]) and 94.3% (ALA_PDT [52] were reported in the active arms of the six studies. Five of the eleven studies reported a percentage reduction in the placebo / vehicle arms, ranging from 25% [59] to 46.9% [62]. A further two studies [53, 54] reported the mean and SD change from baseline in the placebo / vehicle arms.

Summary of interventions:

- TIRBA1%: Both studies [53, 54] reported a mean and standard deviation (SD) at baseline and endpoint. In these studies, there was a significant reduction from around six lesions to one or two lesions in the intervention arm ($p<0.001$) compared with a reduction from around six lesions to around four lesions in the placebo / vehicle arm.
- ALA_PDT: One study [52] reported a reduction from baseline of 94.3% compared with reduction of 32.8% in placebo / vehicle arm.
- DICLO3%: One study [79] showed a 54.6% reduction from baseline in the intervention arm compared with a 36.5% reduction in the placebo arm. Two additional studies [75, 91] reported a reduction from baseline of 51.8% and 57.7%, respectively, but with no placebo / vehicle data for comparison. A final study [84] showed a reduction from a baseline median of seven (range: 5 to 12) to three (range 0 to 8) at endpoint. This last study did not contain a placebo / vehicle arm.
- IM0.015%: One study [84] reported a baseline median of seven (range: 5 to 13) which reduced to two (range: 0 to 6). The small overlap in the ranges indicates a substantial difference between baseline and endpoint. No placebo / vehicle for comparison. A second study [75] reported a reduction from baseline of 80.2%. No placebo / vehicle for comparison.
- IMQ3.75%: One study [59] reported a reduction from baseline of 81.8% compared with a reduction of 25% in the placebo / vehicle arm.
- MAL_PDT: Two studies [91] and [64] reported a reduction from baseline of 85.9% and 89%, respectively. One of these [64] included a placebo / vehicle arm which had reduction of 38%. A third study [84] reported a baseline median of nine (range: 5 to 12) which reduced to

two (range: 0 to 4) in the active arm. The lack of overlap in the ranges indicates a substantial difference between the baseline and endpoint data. No placebo / vehicle for comparison.

- 5FU0.5%+SA: Two studies [62] and [79] reported a reduction from baseline of 74.5% and 78% in the intervention arm and 36.5% and 46.9% in the placebo / vehicle arm, respectively.
- 5FU4%: One study [63] reported an 80.1% reduction from baseline in the intervention arm. The reduction in the placebo / vehicle arm was not reported.
- 5FU5%: One study [63] reported a reduction from baseline of 79%. The reduction in the placebo / vehicle arm was not reported.

In conclusion all interventions, including TIRBA1%, showed substantial reductions in lesion counts, including when compared to placebo / vehicle. The comparisons with placebo / vehicle were sparse.

### 3.1.2 Discontinuation due to Adverse Events / Local Skin Reactions

As described in the main manuscript, rather than looking at discontinuation due to any adverse events (AEs), we only assessed discontinuations due to treatment-emergent AEs (TEAEs), treatment-related AEs (TRAEs), local AEs or local skin reactions (LSRs). These were deemed to most likely to provide data on discontinuations related to the study treatment, rather than reflecting the study methods, baseline characteristics of the study population, or events that were independent from the intervention.

A total of 17 studies reported discontinuations due to TEAEs, TRAEs, serious TRAEs, local AEs or LSRs. Thirteen of the 17 studies reported discontinuation rates for a placebo / vehicle arm. A summary of the discontinuation rates in the interventions of interest is presented below:

In summary:

- TIRBA1%: Two studies [53, 54] reported discontinuation rates of 0% due to TRAEs in the intervention and placebo / vehicle arms.
- ALA_PDT: AK03 and AK04 [78] reported discontinuations of 1.4% due to TEAEs. No discontinuation data were reported for the placebo / vehicle arms of either AK03 or AK04. Two further studies [52, 85] reported discontinuations of 0% due to TRAEs in the intervention arm, and a fifth study [73] reported discontinuations of 0.8% due to serious TRAEs. These final three studies all reported discontinuations of 0% in the placebo / vehicle arms.
- CRYO: One study [89] reported discontinuations of 0% due to LSRs. No placebo / vehicle for comparison.
- DICLO3%: One study [79] reported discontinuations of 4.9% due to local TEAEs compared with 1% (as calculated by reviewers) in the placebo / vehicle arm. The second study [71] reported rates of 2.1% and 12.3% in the intervention arm compared with 3.9% and 2.4% in the placebo / vehicle arm due to local TEAEs and "cutaneous side effects", respectively. Data from this study was taken from an EU Clinical Trials Registry (EUCTR) clinical study

report synopsis, and the relationship between local TEAEs and cutaneous side effects is unclear (i.e. whether one is a subset of the other). A third [75] reported discontinuations of 6% due to TRAEs in the DICLO3% arm. No placebo / vehicle for comparison in this final study.

- IM0.015%: Two studies [28, 75] reported discontinuation rates of 0% and 2% due to serious TRAEs and any TRAEs, respectively. No placebo / vehicle for comparison.
- IMQ3.75%: One study [59] reported discontinuations of 0.6% in the active arm and 0.6% in placebo / vehicle arm due to TRAEs.
- IMQ5%_EU: Two studies [28, 51] reported discontinuation rates in the intervention arm of 0% due to serious TRAEs and LSRs. A third study [61] reported a discontinuation rate of 1.6% due to LSRs. Two studies [51, 61] reported placebo / vehicle arm discontinuation rates of 0%.
- MAL_PDT: Three studies reported discontinuations of 0.8% due to local AEs [73] 1.1% due to LSRs [77] and 0 discontinuations for reasons related to treatment [28]. Of the three trials, two had placebo / vehicle arms [73, 77], for which only one reported data [73], with zero discontinuations due to local AEs.
- 5FU0.5%+SA: Two studies [62, 79] reported discontinuations of 1.9% and 3.7% due to TEAEs. One study [62] reported zero discontinuations in placebo / vehicle arm, and the other study [79] reported one discontinuation (1%, as calculated by reviewers). A third study [89] reported discontinuations due to LSRs of 9.1% in the intervention arm compared with 0% in placebo / vehicle arm.
- 5FU5%: One study [28] reported discontinuations of 0% in the active arm due to serious TRAEs. No placebo / vehicle for comparison.

Rates for the active arms were generally low, ranging from 0% (TIRBA1%; two studies [53, 54]; CRYO, one study [89]; IM0.015%, one study [28]; IMQ5%_EU, two studies [28, 51] and MAL_PDT, one study [28]) to 12.3% (DICLO3% [71] with placebo / vehicle arms ranging between 0% and 3.9% [71]. Exact reasons for discontinuation varied across studies.

The two studies assessing TIRBA1% reported 0% discontinuations due to TEAEs, TRAEs, local AEs or LSRs (these studies reported rates of 0% for both the intervention and the placebo / vehicle arms).

In conclusion, while intervention arms generally reported low rates of 2% or below (with even lower rates presented for placebo / vehicle, between 0% and 0.6%), two of the interventions, 5FU0.5%+ SA and DICLO3% [75, 89], reported rates of 9% and up to 12.3% respectively, compared with 0% discontinuation rates for placebo / vehicle (placebo / vehicle rates available for 5FU0.5%+ SA only).

### 3.1.3 Incidence of severe LSRs

Analyses were planned to investigate the relative incidence of severe forms of LSRs. However, on closer investigation of the available data, only a maximum of five studies contributed to the analysis for any of these outcomes.

- Severe redness / erythema: Reported by five studies [53, 54, 59, 61, 74]
- Severe flaking / scaling / dryness: Reported by five studies [53, 54, 59, 61, 74]
- Severe scabbing / crusting: Reported by four studies [53, 54, 59, 61]
- Severe erosion / ulceration: Reported by four studies [53, 54, 59, 61]
- Severe vesicles: Reported by three studies [53, 54, 61].
- Severe swelling / oedema: Reported by five studies [53, 54, 59, 61, 74]
- Severe itching / pruritis: Reported by three studies [53, 54, 79]
- Severe weeping / exudate: Reported by two studies [59, 61]

The following provides a summary of severe LSRs by intervention, as reported by studies assessing one course or session of treatment only. All LSRs referred to in the following paragraphs are severe.

- TIRBA1%: Across both studies [53, 54] 3% and 10% of patients experienced a severe form of redness / erythema, 6% and 11% experienced flaking / scaling / dryness, no patients experienced erosion, 1% and 3% experienced scabbing / crusting, 0.6% experienced vesicles, 0.6% experienced swelling / oedema, 0% and 0.6% experienced pruritis, and no data were reported for weeping / exudation. In the placebo / vehicle arm, none of these LSRs were reported [53, 54].
- ALA_PDT: One study [74] reported that no patients in the intervention or placebo / vehicle arms experienced any LSR.
- DICLO3%: One study [79] reported that 7% of patients in the intervention arm experienced a severe form of itching / pruritis compared with 0% in the placebo / vehicle arm. No data on redness / erythema, flaking / scaling / dryness, erosion / ulceration, scabbing / crusting, vesicles, swelling / oedema, or weeping / exudation were presented in this study.
- IMQ5%_EU: One study [61] reported that 31% of patients in the intervention arm experienced redness / erythema (placebo / vehicle arm: 0%), 11.6% experienced flaking / scaling / dryness (placebo / vehicle arm: 0.8%), 10.9% experienced erosion / ulceration (placebo / vehicle arm: 0.8%), 24% experienced scabbing / crusting (placebo / vehicle arm: 1.5%), 1.6% experienced vesicles (placebo / vehicle arm: 0%), 7% experienced swelling / oedema (placebo / vehicle arm: 0%), and 4.7% experienced weeping / exudation (placebo / vehicle arm: 0.8%). No data were presented on itching / pruritis.
- IMQ3.75%: One study [59] reported that 25.2% of patients in the intervention arm experienced redness / erythema (placebo / vehicle arm: 0%), 8.2% experienced flaking / scaling / dryness (placebo / vehicle arm: 1.3%), 10.7% experienced erosion / ulceration (placebo / vehicle arm: 0%), 13.8% experienced scabbing / crusting (placebo / vehicle arm:

0%), 5.7% experienced swelling / oedema (placebo / vehicle arm: 0%), and 5.7% experienced weeping / exudation (placebo / vehicle arm: 0%). This study did not report data on vesicles or itching / pruritis.

- 5FU0.5%+SA: One study [79] reported that 4.8% of patients in the intervention arm experienced a severe form of itching / pruritis compared with 0% in the placebo / vehicle arm. This study did not report data on redness / erythema, flaking / scaling / dryness, erosion / ulceration, scabbing / crusting, vesicles, swelling / oedema, or weeping / exudation.

With incidence of severe LSRs only being reported for six of the interventions of interest, the lack of reporting makes a comparison between drugs difficult. Overall, IMQ5%_EU and IMQ3.75% showed relatively high rates of severe LSRs, whereas no severe LSRs were reported for ALA_PDT.

# 4 Supplementary results of the European NMA

## 4.1 Fixed or random effects models

NMA can be based on either fixed or random effects models. With a fixed effects model, we assume that there is a true treatment effect that is the same across studies, and the differences between studies are due to chance alone. With random effects, we assume that the effects being estimated are not the same, but that they are related and follow a shared distribution. The mean of this distribution is the estimate of the treatment effect. Random effects models can be considered as both more plausible and more conservative, and for this reason this manuscript presents the results of the random rather than fixed effects models.

## 4.2 Sensitivity analyses: plots and networks

The following section presents networks and results plots for the four sensitivity analyses detailed in Section 2.5 of the main manuscript.

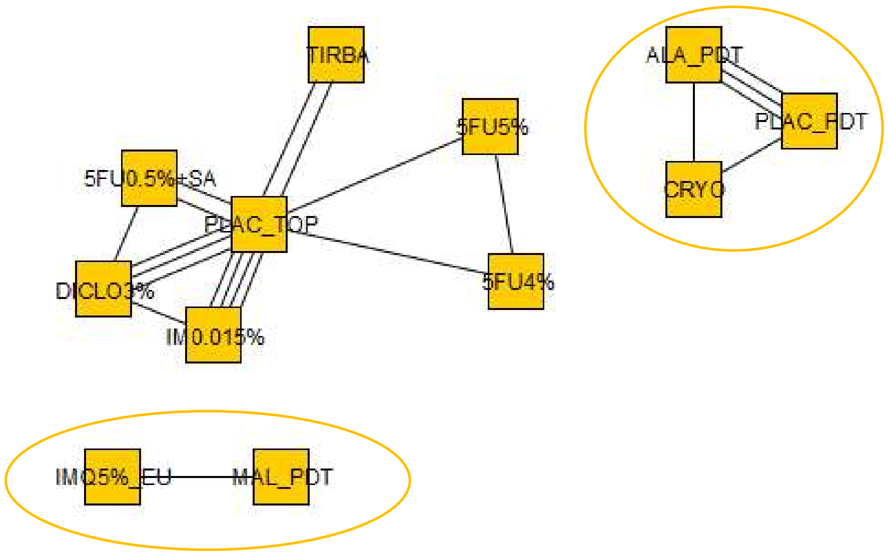


**(a)** Network diagram (circled nodes indicate non-linked subnetworks disconnected from the main network)

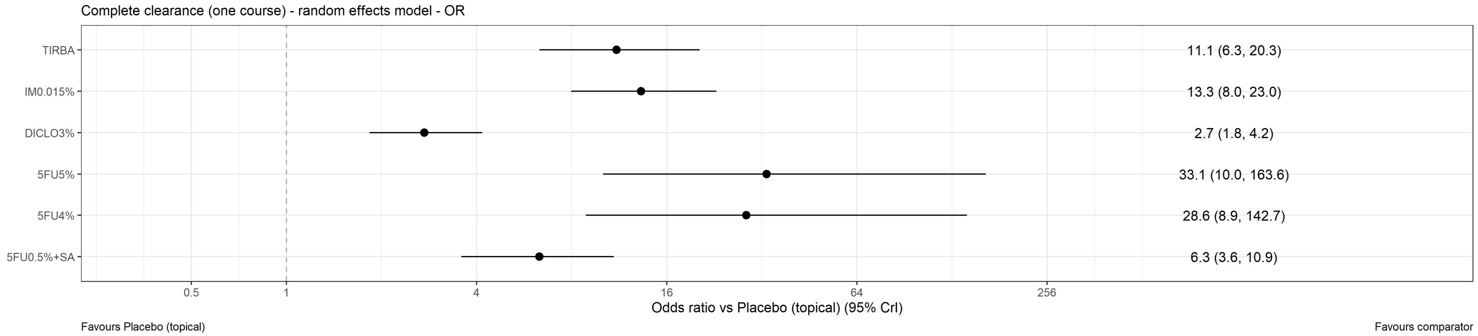


**(b)** Forest plot of the interventions from the main network showing odds ratios against placebo with 95% credible intervals

**Supplementary Figure S2:** Complete clearance, subgroup analysis, only studies assessing a single course or session of treatment – network diagram and forest plot

5FU0.5%+SA - 5 fluorouracil 0.5% + salicylic acid 10%; 5FU4% - 5 fluorouracil 4%; 5FU5% - 5 fluorouracil 5%; ALA_PDT - Photodynamic therapy with 5-Aminolevulinic acid sensitiser; CRYO - Cryotherapy; DICLO3% - Diclofenac 3%; IM0.015% - Ingenol mebutate 0.015%; IMQ5%_EU - Imiquimod 5% EU posology; MAL_PDT - Photodynamic therapy with methyl aminolevulinate sensitiser; PLAC_PDT - Photodynamic therapy with placebo sensitiser; PLAC_TOP - Topical placebo; TIRBA1% - Tirbanibulin 1%

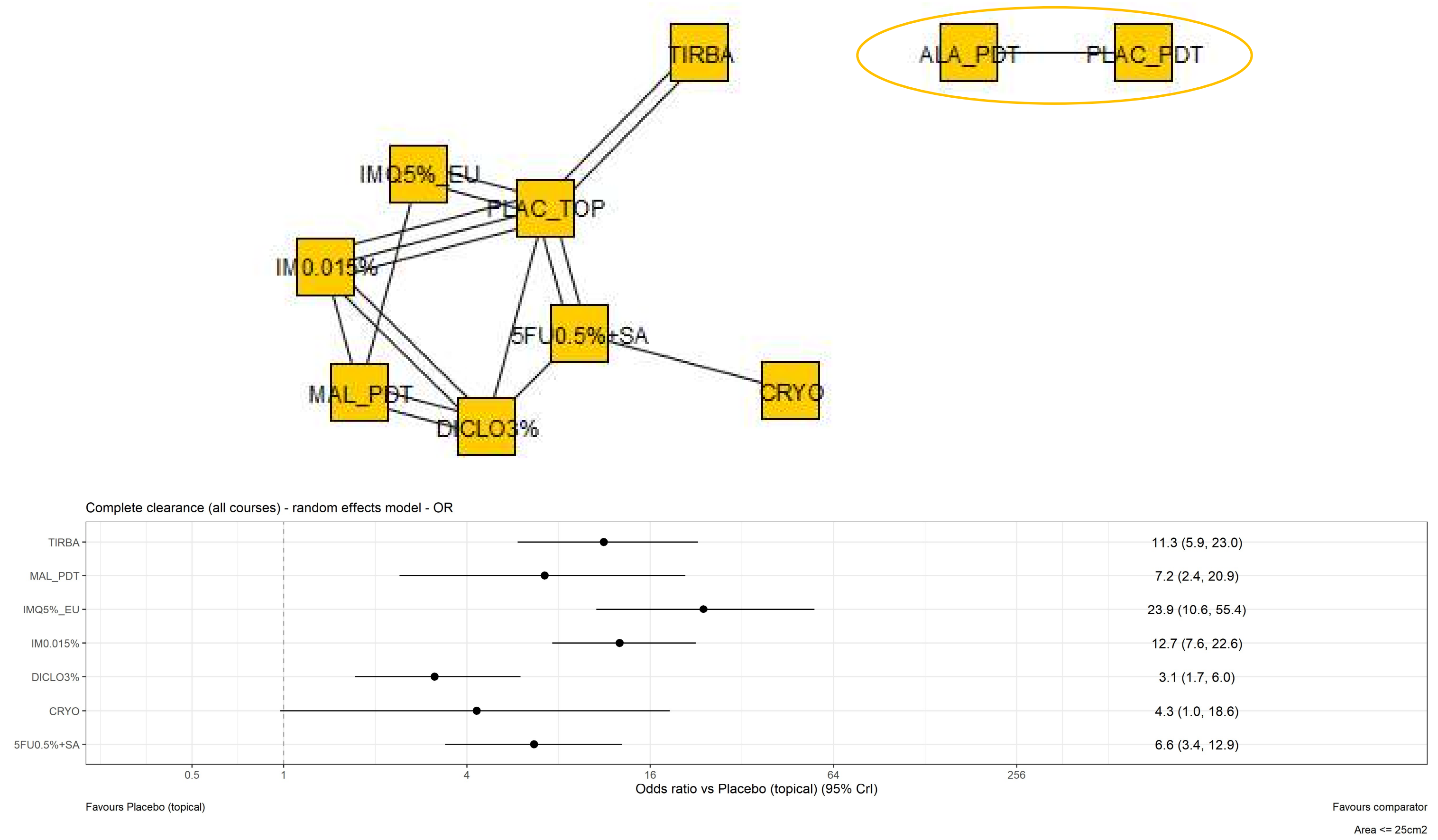


**(a)** Network diagram (circled nodes indicate non-linked subnetworks disconnected from the main network)

**(b)** Forest plot of the interventions from the main network showing odds ratios against placebo with 95% credible intervals

**Supplementary Figure S3:** Complete clearance, subgroup analysis, only studies assessing a treatment area of ≤25 $cm^2$ - network diagram and forest plot

5FU0.5%+SA - 5 fluorouracil 0.5% + salicylic acid 10%; ALA_PDT - Photodynamic therapy with 5-Aminolevulinic acid sensitiser; CRYO - Cryotherapy; DICLO3% - Diclofenac 3%; IM0.015% - Ingenol mebutate 0.015%; IMQ5%_EU - Imiquimod 5% EU posology; MAL_PDT - Photodynamic therapy with methyl aminolevulinate sensitiser; PLAC_PDT - Photodynamic therapy with placebo sensitiser; PLAC_TOP - Topical placebo; TIRBA1% - Tirbanibulin 1%

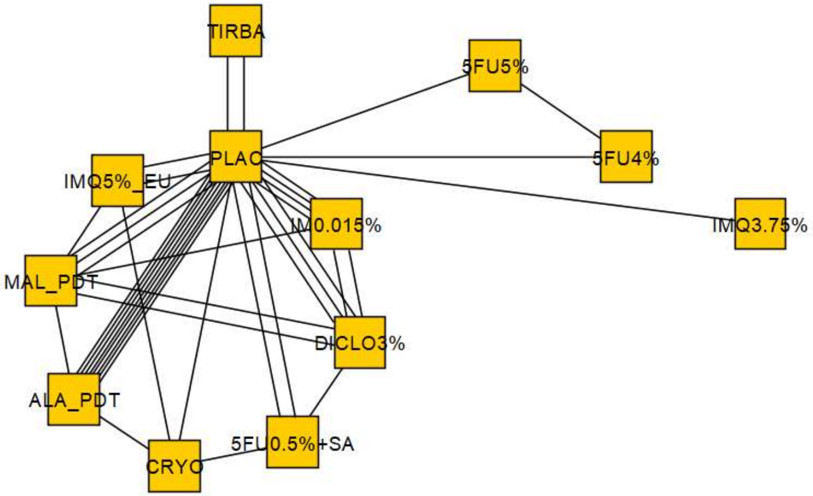


**(a)** Network diagram

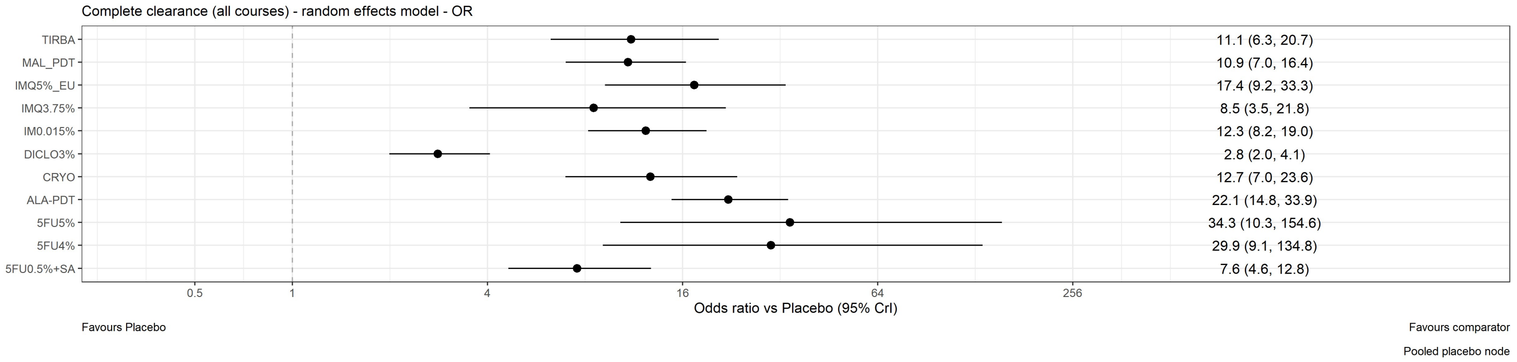


**(b)** Forest plot of the interventions from the main network showing odds ratios against placebo with 95% credible intervals

**Supplementary Figure S4:** Complete clearance, sensitivity analysis, single placebo node

5FU0.5%+SA - 5 fluorouracil 0.5% + salicylic acid 10%; 5FU4% - 5 fluorouracil 4%; 5FU5% - 5 fluorouracil 5%; ALA_PDT - Photodynamic therapy with 5-Aminolevulinic acid sensitiser; CRYO - Cryotherapy; DICLO3% - Diclofenac 3%; IM0.015% - Ingenol mebutate 0.015%; IMQ3.75% - Imiquimod 3.75%; IMQ5%_EU - Imiquimod 5% EU posology; MAL_PDT - Photodynamic therapy with methyl aminolevulinate sensitiser; PLAC - Placebo, topical or placebo PDT; TIRBA1% - Tirbanibulin 1%

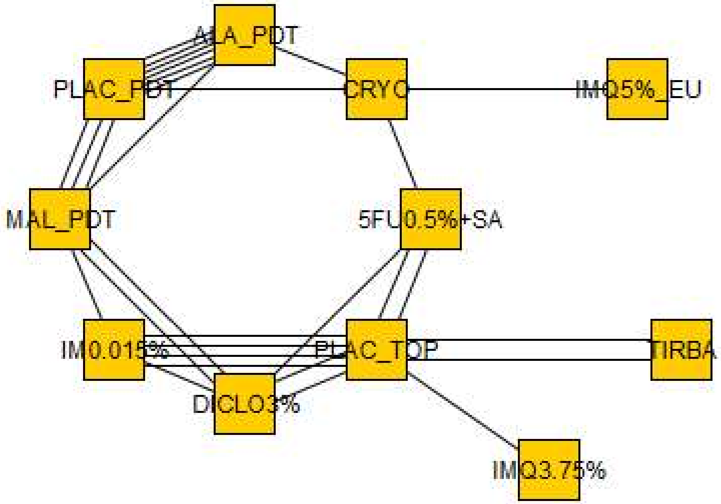


**(a)** Network diagram

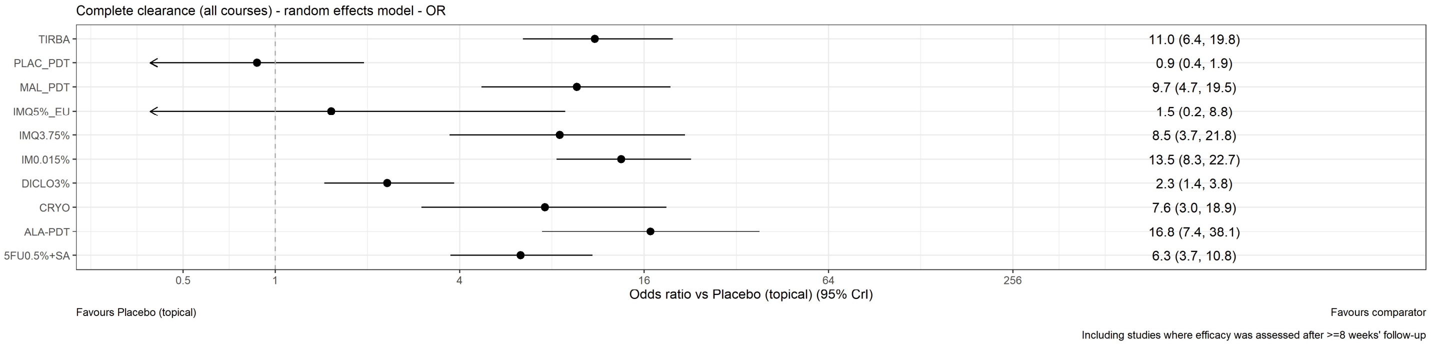


**(b)** Forest plot of the interventions from the main network showing odds ratios against placebo with 95% credible intervals

**Supplementary Figure S5.** Complete clearance, sensitivity analysis, only studies assessing efficacy ≥8 weeks after treatment

5FU0.5%+SA - 5 fluorouracil 0.5% + salicylic acid 10%; ALA_PDT - Photodynamic therapy with 5-Aminolevulinic acid sensitiser; CRYO - Cryotherapy; DICLO3% - Diclofenac 3%; IM0.015% - Ingenol mebutate 0.015%; IMQ3.75% - Imiquimod 3.75%; IMQ5%_EU - Imiquimod 5% EU posology; MAL_PDT - Photodynamic therapy with methyl aminolevulinate sensitiser; PLAC_PDT - Photodynamic therapy with placebo sensitiser; PLAC_TOP - Topical placebo; TIRBA1% - Tirbanibulin 1%

## 4.3 Assessment of Inconsistency

Inconsistency refers to unexplained heterogeneity (i.e. differing treatment effect estimates) between direct comparisons and indirect comparisons made possible by loops in the networks.

Here we estimate the posterior ORs between pairs of treatments for three scenarios: one where we only consider evidence when those treatments are head-to-head; the second where we only consider indirect evidence via shared comparators; and the third where we combine both forms of evidence (which is the result our network reports).

Supplementary Figure S6 shows the results of this inconsistency analysis for those pairs of treatments where both forms of evidence (direct and indirect) were available, and there was significant disagreement (p values <0.05) between the direct and indirect evidence.

We can see that the final network result balances the direct and indirect evidence and does so by upweighting the more confident of the two estimates. In general, indirect posterior ORs were consistent with direct posterior ORs, so the above inconsistencies had little effect on the overall outcome of the analyses.

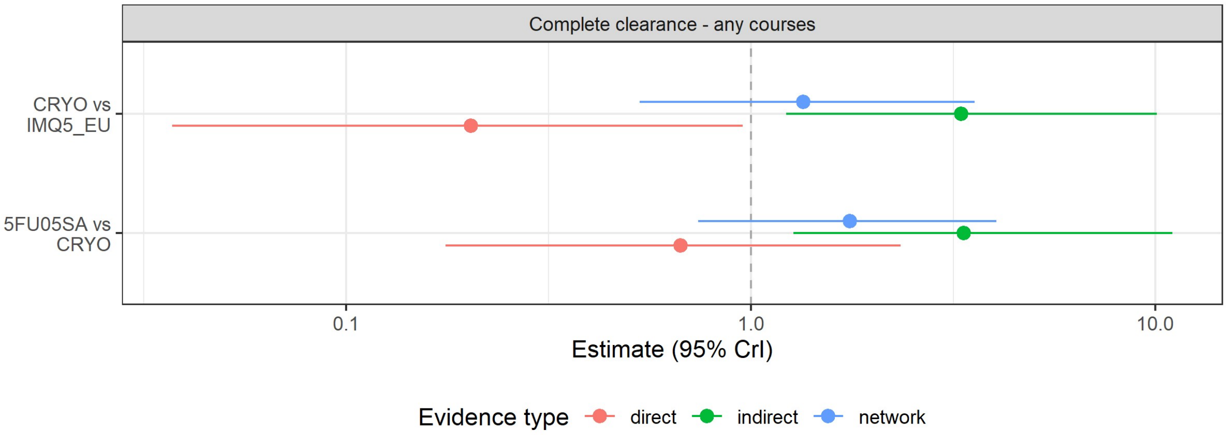


**Supplementary Figure S6.** Inconsistency analysis – complete clearance (any courses)

5FU05SA - 5 fluorouracil 0.5% + salicylic acid 10%; CrI - Credible interval; CRYO - Cryotherapy; IMQ5_EU - Imiquimod 5% EU posology.

# 5 PRISMA Checklist for the review and NMA

**PRISMA NMA Checklist of Items to Include When Reporting A Systematic Review Involving a Network Meta-analysis**

| Section/Topic | Item # | Checklist Item | Reported in? |
|---|---|---|---|
| **TITLE** | | | |
| Title | 1 | Identify the report as a systematic review incorporating a network meta-analysis (or related form of meta-analysis). | Manuscript title |
| | | | |
| **ABSTRACT** | | | |
| Structured summary | 2 | Provide a structured summary including, as applicable:<br>Background: main objectives<br>Methods: data sources; study eligibility criteria, participants, and interventions; study appraisal; and synthesis methods, such as network meta-analysis.<br>Results: number of studies and participants identified; summary estimates with corresponding confidence/credible intervals; treatment rankings may also be discussed. Authors may choose to summarize pairwise comparisons against a chosen treatment included in their analyses for brevity.<br>Discussion/Conclusions: limitations; conclusions and implications of findings.<br>Other: primary source of funding; systematic review registration number with registry name. | Abstract has been constructed in accordance with JCM requirements |
| | | | |
| **INTRODUCTION** | | | |
| Rationale | 3 | Describe the rationale for the review in the context of what is already known, including mention of why a network meta-analysis has been conducted. | Introduction |
| Objectives | 4 | Provide an explicit statement of questions being addressed, with reference to participants, interventions, comparisons, outcomes, and study design (PICOS). | Introduction |
| | | | |
| **METHODS** | | | |
| Protocol and registration | 5 | Indicate whether a review protocol exists and if and where it can be accessed (e.g., Web address); and, if available, provide registration information, including registration number. | Systematic review methods |
| Eligibility criteria | 6 | Specify study characteristics (e.g., PICOS, length of follow-up) and report characteristics (e.g., years considered, language, publication status) used as criteria for eligibility, giving rationale. Clearly describe eligible treatments included in the treatment network, and note whether any have been clustered or merged | Table 1 |

| Section/Topic | Item # | Checklist Item | Reported in? |
|---|---|---|---|
| | | into the same node (with justification). | |
| Information sources | 7 | Describe all information sources (e.g., databases with dates of coverage, contact with study authors to identify additional studies) in the search and date last searched. | Results of the literature searches and screening & Supplementary Table S1 |
| Search | 8 | Present full electronic search strategy for at least one database, including any limits used, such that it could be repeated. | Supplementary Figure S1 |
| Study selection | 9 | State the process for selecting studies (i.e., screening, eligibility, included in systematic review, and, if applicable, included in the meta-analysis). | Systematic review methods & Feasibility assessment methods |
| Data collection process | 10 | Describe method of data extraction from reports (e.g., piloted forms, independently, in duplicate) and any processes for obtaining and confirming data from investigators. | Systematic review methods |
| Data items | 11 | List and define all variables for which data were sought (e.g., PICOS, funding sources) and any assumptions and simplifications made. | Supplementary Section S1.2 |
| Geometry of the network | S1 | Describe methods used to explore the geometry of the treatment network under study and potential biases related to it. This should include how the evidence base has been graphically summarized for presentation, and what characteristics were compiled and used to describe the evidence base to readers. | Statistical analysis methods |
| Risk of bias within individual studies | 12 | Describe methods used for assessing risk of bias of individual studies (including specification of whether this was done at the study or outcome level), and how this information is to be used in any data synthesis. | Systematic review methods |
| Summary measures | 13 | State the principal summary measures (e.g., risk ratio, difference in means). Also describe the use of additional summary measures assessed, such as treatment rankings and surface under the cumulative ranking curve (SUCRA) values, as well as modified approaches used to present summary findings from meta-analyses. | Statistical analysis methods |
| Planned methods of analysis | 14 | Describe the methods of handling data and combining results of studies for each network meta-analysis. This should include, but not be limited to:<br>• Handling of multi-arm trials;<br>• Selection of variance structure;<br>• Selection of prior distributions in Bayesian analyses; and<br>• Assessment of model fit. | Statistical analysis methods |
| Assessment of Inconsistency | S2 | Describe the statistical methods used to evaluate the agreement of direct and indirect evidence in the treatment network(s) studied. Describe efforts taken to address its presence when found. | Statistical analysis methods |
| Risk of bias across studies | 15 | Specify any assessment of risk of bias that may affect the cumulative evidence (e.g., publication bias, selective reporting within studies). | Systematic review methods & Feasibility assessment methods |
| Additional analyses | 16 | Describe methods of additional analyses if done, indicating which were pre-specified. This may include, but not be limited to, the following: | Sensitivity analyses of complete clearance |

| Section/Topic | Item # | Checklist Item | Reported in? |
|---|---|---|---|
| | | • Sensitivity or subgroup analyses;<br>• Meta-regression analyses;<br>• Alternative formulations of the treatment network; and<br>• Use of alternative prior distributions for Bayesian analyses (if applicable). | |
| | | | |
| **RESULTS†** | | | |
| Study selection | 17 | Give numbers of studies screened, assessed for eligibility, and included in the review, with reasons for exclusions at each stage, ideally with a flow diagram. | Results of the literature searches and screening & Figure 1 |
| Presentation of network structure | S3 | Provide a network graph of the included studies to enable visualization of the geometry of the treatment network. | Figure 3 & Supplementary Figures S2–S5 |
| Summary of network geometry | S4 | Provide a brief overview of characteristics of the treatment network. This may include commentary on the abundance of trials and randomized patients for the different interventions and pairwise comparisons in the network, gaps of evidence in the treatment network, and potential biases reflected by the network structure. | Results of the feasibility assessment |
| Study characteristics | 18 | For each study, present characteristics for which data were extracted (e.g., study size, PICOS, follow-up period) and provide the citations. | Supplementary Table S5 |
| Risk of bias within studies | 19 | Present data on risk of bias of each study and, if available, any outcome level assessment. | Figure 2 |
| Results of individual studies | 20 | For all outcomes considered (benefits or harms), present, for each study: 1) simple summary data for each intervention group, and 2) effect estimates and confidence intervals. Modified approaches may be needed to deal with information from larger networks. | Tables 2–4 |
| Synthesis of results | 21 | Present results of each meta-analysis done, including confidence/credible intervals. In larger networks, authors may focus on comparisons versus a particular comparator (e.g. placebo or standard care), with full findings presented in an appendix. League tables and forest plots may be considered to summarize pairwise comparisons. If additional summary measures were explored (such as treatment rankings), these should also be presented. | Figures 3 & Supplementary Figures S2–S5 |
| Exploration for inconsistency | S5 | Describe results from investigations of inconsistency. This may include such information as measures of model fit to compare consistency and inconsistency models, P values from statistical tests, or summary of inconsistency estimates from different parts of the treatment network. | Supplementary Section S4.3 & Supplementary Figure S6 |
| Risk of bias across studies | 22 | Present results of any assessment of risk of bias across studies for the evidence base being studied. | Results of the literature searches and screening |
| Results of additional analyses | 23 | Give results of additional analyses, if done (e.g., sensitivity or subgroup analyses, meta-regression analyses, alternative network geometries studied, alternative choice of prior distributions for Bayesian analyses, and so forth). | Sensitivity analysis: Single course data only<br>Sensitivity analysis: studies |

| Section/Topic | Item # | Checklist Item | Reported in? |
|---|---|---|---|
| | | | assessing a treatment area of ≤25 cm2 only<br>Sensitivity analysis: Single placebo node<br>Sensitivity analysis: Studies assessing efficacy ≥8 weeks after treatment only |
| | | | |
| **DISCUSSION** | | | |
| Summary of evidence | 24 | Summarize the main findings, including the strength of evidence for each main outcome; consider their relevance to key groups (e.g., healthcare providers, users, and policy-makers). | Discussion |
| Limitations | 25 | Discuss limitations at study and outcome level (e.g., risk of bias), and at review level (e.g., incomplete retrieval of identified research, reporting bias). Comment on the validity of the assumptions, such as transitivity and consistency. Comment on any concerns regarding network geometry (e.g., avoidance of certain comparisons). | Limitations & Assumptions |
| Conclusions | 26 | Provide a general interpretation of the results in the context of other evidence, and implications for future research. | Conclusions |
| | | | |
| **FUNDING** | | | |
| Funding | 27 | Describe sources of funding for the systematic review and other support (e.g., supply of data); role of funders for the systematic review. This should also include information regarding whether funding has been received from manufacturers of treatments in the network and/or whether some of the authors are content experts with professional conflicts of interest that could affect use of treatments in the network. | See funding statement |

# References

1. Gupta AK, Paquet M, Villanueva E, Brintnell W. Interventions for actinic keratoses. Cochrane Database Syst Rev. 2012;12:CD004415.
2. de Berker D, McGregor JM, Mohd Mustapa MF, Exton LS, Hughes BR. British Association of Dermatologists' guidelines for the care of patients with actinic keratosis 2017. Br J Dermatol. 2017;176(1):20-43.
3. Reinehr CPH, Bakos RM. Actinic keratoses: review of clinical, dermoscopic, and therapeutic aspects. An Bras Dermatol. 2019;94(6):637-57.
4. Flohil S, Van Der Leest R, Dowlatshahi E, Hofman A, De Vries E, Nijsten T. Prevalence of actinic keratosis and its risk factors in the general population: The rotterdam study. J Invest Dermatol. 2013;133(8):1971-78.
5. Schaefer I, Augustin M, Spehr C, Reusch M, Kornek T. Prevalence and risk factors of actinic keratoses in Germany--analysis of multisource data. J Eur Acad Dermatol Venereol. 2014;28(3):309-13.
6. OPEN VIE. Tirbanibulin as a topical treatment for actinic keratosis: Global Value Dossier [not in the public domain]. Barcelona:  2021.
7. Werner RN, Stockfleth E, Connolly SM, Correia O, Erdmann R, Foley P*, et al.* Evidence- and consensus-based (S3) Guidelines for the Treatment of Actinic Keratosis - International League of Dermatological Societies in cooperation with the European Dermatology Forum - Short version. J Eur Acad Dermatol Venereol. 2015;29(11):2069-79.
8. Chetty P, Choi F, Mitchell T. Primary care review of actinic keratosis and its therapeutic options: a global perspective. Dermatol Ther (Heidelb). 2015;5(1):19-35.
9. Lucas R, McMichael T, Smith W, Armstrong B. Solar ultraviolet radiation: Global burden of disease from solar ultraviolet radiation.  In: Prüss-Üstün A, editor. Environmental burden of disease series ; no. 13.  Geneva. World Health Organization,. 2006.
10. Worldometer. Population of Europe [Webpage]. 2020. [cited October 2021]. Available from: https://www.worldometers.info/world-population/europe-population/.
11. Criscione VD, Weinstock MA, Naylor MF, Luque C, Eide MJ, Bingham SF*, et al.* Actinic keratoses: Natural history and risk of malignant transformation in the Veterans Affairs Topical Tretinoin Chemoprevention Trial. Cancer. 2009;115(11):2523-30.
12. Fernández-Figueras MT, Carrato C, Sáenz X, Puig L, Musulen E, Ferrándiz C*, et al.* Actinic keratosis with atypical basal cells (AK I) is the most common lesion associated with invasive squamous cell carcinoma of the skin. J Eur Acad Dermatol Venereol. 215;29(5):991-7.
13. Athanasakis K, Boubouchairopoulou N, Tarantilis F, Tsiantou V, Kontodimas S, Kyriopoulos J. Cost-effectiveness of Ingenol Mebutate Gel for the Treatment of Actinic Keratosis in Greece. Clin Ther. 2017;39(5):993-1002.
14. Tennvall GR, Norlin JM, Malmberg I, Erlendsson AM, Haedersdal M. Health related quality of life in patients with actinic keratosis--an observational study of patients treated in dermatology specialist care in Denmark. Health Qual Life Outcomes. 2015;13:111.
15. Gulko C. Cutaneous Squamous Cell Carcinoma: Expensive to Treat. MedPage Today,; 2020. [cited 01 October 2021]. Available from: https://www.medpagetoday.com/resource-centers/contemporary-approaches-non-melanoma-skin-cancer/cutaneous-squamous-cell-carcinoma-expensive-treat/2747.
16. British National Formulary (BNF). Photodamage [Webpage]. London & Manchester: National Institute for Health and Care Excellence (NICE),; 2021. [cited 01 October 2021]. Available from: https://bnf.nice.org.uk/treatment-summary/photodamage.html.
17. Stockfleth E. The importance of treating the field in actinic keratosis. J Eur Acad Dermatol Venereol. 2017;31 Suppl 2:8-11.
18. National Institute for Health and Care Excellence. Actinic keratosis: ingenol mebutate gel: evidence summary [ESN14]. Manchester: NICE; 2013. Available from: https://www.nice.org.uk/advice/esnm14/chapter/Overview.
19. Heppt MV, Leiter U, Steeb T, Amaral T, Bauer A, Becker JC*, et al.* S3 guideline for actinic keratosis and cutaneous squamous cell carcinoma - short version, part 1: diagnosis,

interventions for actinic keratoses, care structures and quality-of-care indicators. J Dtsch Dermatol Ges. 2020;18(3):275-94.
20. European Medicines Agency. Summary of Product Characteristics: Klisyri 10 mg/g ointment [Webpage]. Amsterdam: European Medicines Agency; 2021. Last updated August 2021. [cited January 2022]. Available from: https://www.ema.europa.eu/en/documents/product-information/klisyri-epar-product-information_en.pdf.
21. Vegter S, Tolley K. A network meta-analysis of the relative efficacy of treatments for actinic keratosis of the face or scalp in Europe. PLoS ONE. 2014;9(6):e96829.
22. Steeb T, Wessely A, Petzold A, Brinker TJ, Schmitz L, Leiter U*, et al.* Evaluation of Long-term Clearance Rates of Interventions for Actinic Keratosis: A Systematic Review and Network Meta-analysis. JAMA Dermatol. 2021;157(9):1066-77.
23. Werner RN, Sammain A, Erdmann R, Hartmann V, Stockfleth E, Nast A. The natural history of actinic keratosis: a systematic review. Br J Dermatol. 2013;169(3):502-18.
24. Higgins JPT, Thomas J, Chandler J, Cumpston M, Li T, Page MJ*, et al.*, editors. Cochrane Handbook for Systematic Reviews of Interventions 2nd edition (updated October 2019). www.cochrane-handbook.org: The Cochrane Collaboration; 2019.
25. Centre for Reviews and Dissemination. PROSPERO [online database]. York: CRD; 2011. Last updated 29 June 2015. Available from: http://www.crd.york.ac.uk/PROSPERO/.
26. European Medicines Agency. Risks of Picato for actinic keratosis outweigh benefits. Amsterdam: European Medicines Agency; 2020. [cited 30 September 2021]. Available from: https://www.ema.europa.eu/en/medicines/human/referrals/picato.
27. Jansen MHE, Kessels J, Merks I, Nelemans PJ, Kelleners-Smeets NWJ, Mosterd K*, et al.* A trial-based cost-effectiveness analysis of topical 5-fluorouracil vs. imiquimod vs. ingenol mebutate vs. methyl aminolaevulinate conventional photodynamic therapy for the treatment of actinic keratosis in the head and neck area performed in the Netherlands. Br J Dermatol. 2020;21:21.
28. Jansen MHE, Kessels J, Nelemans PJ, Kouloubis N, Arits A, van Pelt HPA*, et al.* Randomized trial of four treatment approaches for actinic keratosis. N Engl J Med. 2019;380(10):935-46.
29. Australian Government Department of Health and Ageing Pharmaceutical Benefits Advisory Committee (PBAC). Appendix 4: Heterogeneity of treatment effect across studies. In: Guidelines for preparing submissions to the Pharmaceutical Benefits Advisory Committee. Canberra, Australia. Pharmaceutical Benefits Advisory Committee (PBAC). 2016. Available from: https://pbac.pbs.gov.au/appendixes/appendix-4-heterogeneity-of-treatment-effect-across-studies.html#Table-a4-1.
30. Dias S, Welton NJ, Sutton AJ, Ades AE. Technical Support Document 2: A general linear modelling framework for pair-wise and network meta-analysis of randomised controlled trials. Sheffield: 2011 (updated September 2016). Available from: http://www.nicedsu.org.uk/TSD2%20General%20meta%20analysis%20corrected%202Sep2016v2.pdf.
31. Turner RM, Jackson D, Wei Y, Thompson SG, Higgins JP. Predictive distributions for between-study heterogeneity and simple methods for their application in Bayesian meta-analysis. Stat Med. 2015;34(6):984-98.
32. Decision Support Unit. NICE DSU Technical Support Document 4: Inconsistency in Networks of Evidence Based on Randomised Controlled Trials. Sheffield: 2014. Available from: http://nicedsu.org.uk/wp-content/uploads/2016/03/TSD4-Inconsistency.final_.15April2014.pdf.
33. Plummer M. JAGS: A Program for Analysis of Bayesian Graphical Models Using Gibbs Sampling. In: 3rd International Workshop on Distributed Statistical Computing (DSC 2003), Vienna, Austria; 2003.
34. R Core Team. R: a language and environment for statistical computing. Vienna, Austria: R Foundation for Statistical Computing; 2018. Available from: https://www.R-project.org/.
35. Plummer M. rjags: Bayesian graphical models using MCMC. R package version. 2016;4(6)
36. Python Software Foundation. Python Language Reference, version 3.7. Beaverton OR, : Python Software Foundation; 2018. Available from: http://www.python.org.

37. Hartikainen et al. PyStan Release v3.0.0rc1. Read the Docs; 2019. Available from: https://pystan.readthedocs.io/en/latest/.
38. Stan Development Team. Stan Modeling Language Users Guide and Reference Manual, 2.25. 2020. Available from: https://mc-stan.org.
39. Datapharm. Summary of Product Characteristics: Efudix 5% Cream. Leatherhead: Datapharm; 2020. [cited 4 April 2021]. Available from: https://www.medicines.org.uk/emc/product/9260/smpc.
40. Datapharm. Summary of Product Characteristics: Actikerall 5mg/g + 100mg/g Cutaneous Solution. Leatherhead: Datapharm; 2016. [cited 4 April 2021]. Available from: https://www.medicines.org.uk/emc/product/4621/smpc.
41. European Medicines Agency. Summary of Product Characteristics: Ameluz 78 mg/g gel. Amsterdam: European Medicines Agency; 2016. [cited January 2022]. Available from: https://www.ema.europa.eu/en/documents/product-information/ameluz-epar-product-information_en.pdf.
42. Datapharm. Summary of Product Characteristics: Metvix 160 mg/g cream. Leatherhead: Datapharm; 2021. [cited 4 April 2021]. Available from: https://www.medicines.org.uk/emc/product/6777/smpc.
43. Datapharm. Summary of Product Characteristics: Solaraze 3% Gel. Leatherhead: Datapharm; 2007. [cited 4 April 2021]. Available from: https://www.medicines.org.uk/emc/product/6385.
44. European Medicines Agency. Summary of Product Characteristics: Aldara 5% Cream. Amsterdam: European Medicines Agency; 2008. [cited January 2022]. Available from: https://www.ema.europa.eu/en/documents/product-information/aldara-epar-product-information_en.pdf.
45. European Medicines Agency. Summary of Product Characteristics: Zyclara 3.75% cream. Amsterdam: European Medicines Agency; 2017. [cited January 2022]. Available from: https://www.ema.europa.eu/en/documents/product-information/zyclara-epar-product-information_en.pdf.
46. Datapharm. Summary of Product Characteristics: Picato 150 mcg/g Gel. Leatherhead: Datapharm; 2019. [cited 4 April 2021]. Available from: https://www.medicines.org.uk/emc/product/2888/smpc.
47. Ezzedine K, Painchault C, Brignone M. Systematic literature review and network meta-analysis of the efficacy and acceptability of interventions in actinic keratoses. Acta Derm Venereol. 2021; 101(1): 1-8. Available from: https://www.medicaljournals.se/acta/content/abstract/10.2340/00015555-3690
48. Gupta AK, Paquet M. Network meta-analysis of the outcome 'participant complete clearance' in nonimmunosuppressed participants of eight interventions for actinic keratosis: a follow-up on a Cochrane review. Br J Dermatol. 2013;169(2):250-9.
49. Weinstein GD, McCullough JL, Ross P. Cell proliferation in normal epidermis. J Invest Dermatol. 1984;82(6):623-8.
50. Maeda K. New Method of Measurement of Epidermal Turnover in Humans. Cosmetics. 2017;4(4):47.
51. Chen K, Yap LM, Marks R, Shumack S. Short-course therapy with imiquimod 5% cream for solar keratoses: a randomized controlled trial. Australas J Dermatol. 2003;44(4):250-5.
52. Reinhold U, Dirschka T, Ostendorf R, Aschoff R, Berking C, Philipp-Dormston WG, *et al.* A randomized, double-blind, phase III, multicentre study to evaluate the safety and efficacy of BF-200 ALA (Ameluz( R) ) vs. placebo in the field-directed treatment of mild-to-moderate actinic keratosis with photodynamic therapy (PDT) when using the BF-RhodoLED( R) lamp. Br J Dermatol. 2016;175(4):696-705.
53. Athenex. Clinical Study Report: [KX01-AK-003] A Phase 3, Double-Blind, Vehicle-Controlled, Randomized, Parallel Group, Multicenter, Efficacy and Safety Study of KX2-391 Ointment 1% in Adult Subjects with Actinic Keratosis on the Face or Scalp. New Jersey, USA: Athenex Inc.; 09 August 2019a. 1-619.
54. Athenex. Clinical Study Report: [KX01-AK-004] A Phase 3, Double-Blind, Vehicle-Controlled, Randomized, Parallel Group, Multicenter, Efficacy and Safety Study of KX2-391

Ointment 1% in Adult Subjects with Actinic Keratosis on the Face or Scalp. New Jersey, USA: Athenex Inc.; 09 September 2019b. 1-620.
55. Szeimies RM, Gerritsen MJ, Gupta G, Ortonne JP, Serresi S, Bichel J*, et al.* Imiquimod 5% cream for the treatment of actinic keratosis: results from a phase III, randomized, double-blind, vehicle-controlled, clinical trial with histology. J Am Acad Dermatol. 2004;51(4):547-55.
56. Lebwohl M, Dinehart S, Whiting D, Lee PK, Tawfik N, Jorizzo J*, et al.* Imiquimod 5% cream for the treatment of actinic keratosis: results from two phase III, randomized, double-blind, parallel group, vehicle-controlled trials. J Am Acad Dermatol. 2004;50(5):714-21.
57. Korman N, Moy R, Ling M, Matheson R, Smith S, McKane S*, et al.* Dosing with 5% imiquimod cream 3 times per week for the treatment of actinic keratosis: results of two phase 3, randomized, double-blind, parallel-group, vehicle-controlled trials. Arch Dermatol. 2005;141(4):467-73.
58. Hanke CW, Beer KR, Stockfleth E, Wu J, Rosen T, Levy S. Imiquimod 2.5% and 3.75% for the treatment of actinic keratoses: results of two placebo-controlled studies of daily application to the face and balding scalp for two 3-week cycles. J Am Acad Dermatol. 2010;62(4):573-81.
59. Swanson N, Abramovits W, Berman B, Kulp J, Rigel DS, Levy S. Imiquimod 2.5% and 3.75% for the treatment of actinic keratoses: results of two placebo-controlled studies of daily application to the face and balding scalp for two 2-week cycles. J Am Acad Dermatol. 2010;62(4):582-90.
60. Jorizzo J, Dinehart S, Matheson R, Moore JK, Ling M, Fox TL*, et al.* Vehicle-controlled, double-blind, randomized study of imiquimod 5% cream applied 3 days per week in one or two courses of treatment for actinic keratoses on the head. J Am Acad Dermatol. 2007;57(2):265-8.
61. Alomar A, Bichel J, McRae S. Vehicle-controlled, randomized, double-blind study to assess safety and efficacy of imiquimod 5% cream applied once daily 3 days per week in one or two courses of treatment of actinic keratoses on the head. Br J Dermatol. 2007;157(1):133-41.
62. Stockfleth E, von Kiedrowski R, Dominicus R, Ryan J, Ellery A, Falques M*, et al.* Efficacy and safety of 5-fluorouracil 0.5%/salicylic acid 10% in the field-directed treatment of actinic keratosis: a phase III, randomized, double-blind, vehicle-controlled trial. Dermatol Ther. 2017;7(1):81-96.
63. Dohil MA. Efficacy, Safety, and Tolerability of 4% 5-Fluorouracil Cream in a Novel Patented Aqueous Cream Containing Peanut Oil Once Daily Compared With 5% 5-Fluorouracil Cream Twice Daily: Meeting the Challenge in the Treatment of Actinic Keratosis. J Drugs Dermatol. 2016;15(10):1218-24.
64. Pariser DM, Lowe NJ, Stewart DM, Jarratt MT, Lucky AW, Pariser RJ*, et al.* Photodynamic therapy with topical methyl aminolevulinate for actinic keratosis: results of a prospective randomized multicenter trial. J Am Acad Dermatol. 2003;48(2):227-32.
65. Peplin. A multicenter study to evaluate the safety and efficacy of PEP005 topical gel when used to treat actinic keratoses on the head (face or scalp). Identifier: NCT00700063. In: ClinicalTrials.gov [internet]. Bethesda: US National Library of Medicine: 2008. Available from https://ClinicalTrials.gov/show/NCT00700063.
66. Peplin. A multi-center study to evaluate the efficacy and safety of pep005 (ingenol mebutate) gel, when used to treat actinic keratoses on the head (face or scalp). Identifier: NCT00915551. In: ClinicalTrials.gov [internet]. Bethesda: US National Library of Medicine: 2009a. Available from https://ClinicalTrials.gov/show/NCT00915551.
67. Peplin. A multi-center study to evaluate the efficacy and safety of pep005 (ingenol mebutate) gel, when used to treat actinic keratoses on the head (face or scalp). Identifier: NCT00916006. In: ClinicalTrials.gov [internet]. Bethesda: US National Library of Medicine: 2009b. Available from https://ClinicalTrials.gov/show/NCT00916006.
68. Gage Development Company. Study comparing GDC 695 and diclofenac sodium gel, 3% in subjects with actinic keratoses. Identifier: NCT02952898. In: ClinicalTrials.gov [internet]. Bethesda: US National Library of Medicine: 2016. Available from https://ClinicalTrials.gov/show/NCT02952898.
69. Actavis Inc. An equivalence study of generic ingenol mebutate gel 0.015% and picato gel 0.015% in subjects with actinic keratosis. Identifier: NCT03200912. In: ClinicalTrials.gov

[internet]. Bethesda: US National Library of Medicine: 2016. Available from https://ClinicalTrials.gov/show/NCT03200912.
70. Dermapharm AG. Multicenter, randomised, double-blind clinical trial on the efficacy and safety of medicinal products containing diclofenac in patients with actinic keratosis. Identifier: EUCTR2014-001621-33-DE. In: EU Clinical Trials Register [internet]. London: European Medicines Agency: 2014. Available from https://www.clinicaltrialsregister.eu/ctr-search/trial/2014-001621-33/DE/.
71. Almirall S.A. Double-blind, randomized, vehicle- and comparator-controlled, multicenter trial to evaluate the efficacy and safety of LAS41007 in the treatment of actinic keratosis. Identifier: EUCTR2010-022244-20-DE. In: EU Clinical Trials Register [internet]. London: European Medicines Agency: 2010. Available from https://www.clinicaltrialsregister.eu/ctr-search/trial/2010-022244-20/DE/.
72. Pariser D, Loss R, Jarratt M, Abramovits W, Spencer J, Geronemus R*, et al.* Topical methyl-aminolevulinate photodynamic therapy using red light-emitting diode light for treatment of multiple actinic keratoses: a randomized, double-blind, placebo-controlled study. J Am Acad Dermatol. 2008;59(4):569-76.
73. Dirschka T, Radny P, Dominicus R, Mensing H, Bruning H, Jenne L*, et al.* Photodynamic therapy with BF-200 ALA for the treatment of actinic keratosis: results of a multicentre, randomized, observer-blind phase III study in comparison with a registered methyl-5-aminolaevulinate cream and placebo. Br J Dermatol. 2012;166(1):137-46.
74. Pariser DM, Houlihan A, Ferdon MB, Berg JE, Group P-AI. Randomized vehicle-controlled study of short drug incubation aminolevulinic acid photodynamic therapy for actinic keratoses of the face or scalp. Dermatol Surg. 2016;42(3):296-304.
75. Stockfleth E, Harwood CA, Serra-Guillen C, Larsson T, Osterdal ML, Skov T. Phase IV head-to-head randomized controlled trial comparing ingenol mebutate 0.015% gel with diclofenac sodium 3% gel for the treatment of actinic keratosis on the face or scalp. Br J Dermatol. 2018;178(2):433-42.
76. MEDA Pharma GmbH & Co. KG. Long-term effects of Aldara® 5% cream and Solaraze® 3% gel in the treatment of actinic keratoses on the face or scalp (LEIDA). Identifier: EUCTR2007-004884-24-DE. In: EU Clinical Trials Register [internet]. London: European Medicines Agency: 2015. Available from https://www.clinicaltrialsregister.eu/ctr-search/trial/2007-004884-24/DE.
77. Freeman M, Vinciullo C, Francis D, Spelman L, Nguyen R, Fergin P*, et al.* A comparison of photodynamic therapy using topical methyl aminolevulinate (Metvix) with single cycle cryotherapy in patients with actinic keratosis: a prospective, randomized study. J Dermatolog Treat. 2003;14(2):99-106.
78. Hauschild A, Stockfleth E, Popp G, Borrosch F, Bruning H, Dominicus R*, et al.* Optimization of photodynamic therapy with a novel self-adhesive 5-aminolaevulinic acid patch: results of two randomized controlled phase III studies. Br J Dermatol. 2009;160(5):1066-74.
79. Almirall Hermal GmbH. Study on the efficacy of Verrumal(R) compared to placebo and Solaraze(R) in the treatment of actinic keratosis grade I to II. Identifier: EUCTR2007-003889-18-DE. In: EU Clinical Trials Register [internet]. London: European Medicines Agency: 2007. Available from https://www.clinicaltrialsregister.eu/ctr-search/trial/2007-003889-18/DE/.
80. Taro Pharmaceuticals USA. Bioequivalence study of two imiquimod cream 5%. Identifier: NCT00828568. In: ClinicalTrials.gov [internet]. Bethesda: US National Library of Medicine: 2008. Available from https://ClinicalTrials.gov/show/NCT00828568.
81. Biofrontera Bioscience GmbH. A randomized placebo-controlled clinical trial of topical photodynamic therapy with a nanoemulsion formulation of 5-aminolevulinic acid for the treatment of actinic keratosis. Identifier: EUCTR2006-000314-20-DE. In: EU Clinical Trials Register [internet]. London: European Medicines Agency: 2006. Available from https://www.clinicaltrialsregister.eu/ctr-search/trial/2006-000314-20/DE/.
82. Szeimies RM, Radny P, Sebastian M, Borrosch F, Dirschka T, Krahn-Senftleben G*, et al.* Photodynamic therapy with BF-200 ALA for the treatment of actinic keratosis: results of a prospective, randomized, double-blind, placebo-controlled phase III study. Br J Dermatol. 2010;163(2):386-94.

83. Tanghetti E, Werschler P. Comparison of 5% 5-fluorouracil cream and 5% imiquimod cream in the management of actinic keratoses on the face and scalp. J Drugs Dermatol. 2007;6(2):144-7.
84. Arisi M, Zane C, Polonioli M, Tomasi C, Moggio E, Cozzi C*, et al.* Effects of MAL-PDT, ingenol mebutate and diclofenac plus hyaluronate gel monitored by high-frequency ultrasound and digital dermoscopy in actinic keratosis- a randomized trial. J Eur Acad Dermatol Venereol. 2020;34(6):1225-32.
85. Piacquadio DJ, Chen DM, Farber HF, Fowler JF, Jr., Glazer SD, Goodman JJ*, et al.* Photodynamic therapy with aminolevulinic acid topical solution and visible blue light in the treatment of multiple actinic keratoses of the face and scalp: investigator-blinded, phase 3, multicenter trials. Arch Dermatol. 2004;140(1):41-6.
86. Foley P, Merlin K, Cumming S, Campbell J, Crouch R, Harrison S*, et al.* A comparison of cryotherapy and imiquimod for treatment of actinic keratoses: lesion clearance, safety, and skin quality outcomes. J Drugs Dermatol. 2011;10(12):1432-8.
87. Samorano LP, Torezan LA, Sanches JA. Evaluation of the tolerability and safety of a 0.015% ingenol mebutate gel compared to 5% 5-fluorouracil cream for the treatment of facial actinic keratosis: a prospective randomized trial. J Eur Acad Dermatol Venereol. 2015;29(9):1822-7.
88. Kose O, Koc E, Erbil AH, Caliskan E, Kurumlu Z. Comparison of the efficacy and tolerability of 3% diclofenac sodium gel and 5% imiquimod cream in the treatment of actinic keratosis. J Dermatolog Treat. 2008;19(3):159-63.
89. Simon JC, Dominicus R, Karl L, Rodriguez R, Willers C, Dirschka T. A prospective randomized exploratory study comparing the efficacy of once-daily topical 0.5% 5-fluorouracil in combination with 10.0% salicylic acid (5-FU/SA) vs. cryosurgery for the treatment of hyperkeratotic actinic keratosis. J Eur Acad Dermatol Venereol. 2015;29(5):881-9.
90. MEDA Pharma GmbH & Co. KG. Long-term effects of Aldara® 5% cream and Solaraze® 3% gel in the treatment of actinic keratoses on the face or scalp with respect to the risk of progression to in-situ and invasive squamous cell carcinoma (LEIDA 2). Identifier: EUCTR2010-022054-16-DE. In: EU Clinical Trials Register [internet]. London: European Medicines Agency: 2010. Available from https://www.clinicaltrialsregister.eu/ctr-search/trial/2010-022054-16/DE/.
91. Zane C, Facchinetti E, Rossi MT, Specchia C, Calzavara-Pinton PG. A randomized clinical trial of photodynamic therapy with methyl aminolaevulinate vs. diclofenac 3% plus hyaluronic acid gel for the treatment of multiple actinic keratoses of the face and scalp. Br J Dermatol. 2014;170(5):1143-50.
92. Serra-Guillen C, Nagore E, Hueso L, Traves V, Messeguer F, Sanmartin O*, et al.* A randomized pilot comparative study of topical methyl aminolevulinate photodynamic therapy versus imiquimod 5% versus sequential application of both therapies in immunocompetent patients with actinic keratosis: clinical and histologic outcomes. J Am Acad Dermatol. 2012;66(4):e131-7.
93. Berman B, Nestor MS, Newburger J, Park H, Swenson N. Treatment of facial actinic keratoses with aminolevulinic acid photodynamic therapy (ALA-PDT) or ingenol mebutate 0.015% gel with and without prior treatment with ALA-PDT. J Drugs Dermatol. 2014;13(11):1353-6.
94. Dermapharm AG. Double-blind, randomized, clinical trial to compare the efficacy and safety of diclofenc 3% gel vs. solaraze 3% gel vs. vehicle for the treatment of patients with actinic keratosis. Identifier: EUCTR2011-003317-41-DE. In: EU Clinical Trials Register [internet]. London: European Medicines Agency: 2011. Available from https://www.clinicaltrialsregister.eu/ctr-search/trial/2011-003317-41/DE/.
95. McGuinness L, Higgins J. Risk-of-bias VISualization (robvis): An R package and Shiny web app for visualizing risk-of-bias assessments. Res Syn Meth. 2020:1-7.
96. US Food and Drug Administration. Aldara® (imiquimod) Cream, 5%. Silver Springs: US Food and Drug Administration; 2010. [cited 4 April 2021]. Available from: https://www.accessdata.fda.gov/drugsatfda_docs/label/2010/020723s022lbl.pdf.

97. US Food and Drug Administration. Highlights of Prescribing Information: KLISYRI (tirbanibulin) ointment, for topical use 2020. [cited 04.02 2021]. Available from: https://www.accessdata.fda.gov/drugsatfda_docs/label/2020/213189s000lbl.pdf.
98. Steeb T, Wessely A, Schmitz L, Heppt F, Kirchberger MC, Berking C*, et al.* Interventions for Actinic Keratosis in Nonscalp and Nonface Localizations: Results from a Systematic Review with Network Meta-Analysis. J Invest Dermatol. 2021;141(2):345-54 e8.
99. Wu Y, Tang N, Cai L, Li Q. Relative efficacy of 5-fluorouracil compared with other treatments among patients with actinic keratosis: A network meta-analysis. Dermatol Ther. 2019;32(3):e12822.
100. The Pharma Letter. Denmark's LEO Pharma initiates Picato phase-out. London: The Pharma Letter; 2020. [cited 30 September 2021]. Available from: https://www.thepharmaletter.com/article/denmark-s-leo-pharma-initiates-picato-phase-out.
101. Shergill B, Zokaie S, Carr AJ. Non-adherence to topical treatments for actinic keratosis. Patient Prefer Adher. 2013;8:35-41.
102. Erntoft S, Norlin JM, Pollard C, Diepgen TL. Patient-reported adherence and persistence to topical treatments for actinic keratosis: a longitudinal diary study. Br J Dermatol. 2016;175(5):1094-96.
103. Scottish Medicines Consortium (SMC). Detailed advice: tirbanibulin 10mg/g ointment (Klisyri®). Glasgow: SMC; 13 December 2021. Available from: https://www.scottishmedicines.org.uk/media/6538/tirbanibulin-klisyri-final-november-2021-for-website.pdf.
